# Nanoscale Organolanthanum Clusters: Nuclearity‐Directing Role of Cyclopentadienyl and Halogenido Ligands

**DOI:** 10.1002/chem.202001482

**Published:** 2020-07-27

**Authors:** Dennis A. Buschmann, H. Martin Dietrich, David Schneider, Verena M. Birkelbach, Christoph Stuhl, Karl W. Törnroos, Cäcilia Maichle‐Mössmer, Reiner Anwander

**Affiliations:** ^1^ Institut für Anorganische Chemie Eberhard Karls Universität Tübingen Auf der Morgenstelle 18 72076 Tübingen Germany; ^2^ Department of Chemistry University of Bergen Allégaten 41 5007 Bergen Norway

**Keywords:** clusters, cyclopentadienyl, halogenido, lanthanum, self-assembly

## Abstract

Tetramethylaluminato/halogenido(X) ligand exchange reactions in half‐sandwich complexes [Cp^R^La(AlMe_4_)_2_] are feasible in non‐coordinating solvents and provide access to large coordination clusters of the type [Cp^R^LaX_2_]_*x*_. Incomplete exchange reactions generate the hexalanthanum clusters [Cp^R^_6_La_6_X_8_(AlMe_4_)_4_] (Cp^R^=Cp*=C_5_Me_5_, X=I; Cp^R^=Cp′=C_5_H_4_SiMe_3_, X=Br, I). Treatment of [Cp*La(AlMe_4_)_2_] with two equivalents Me_3_SiI gave the nonalanthanum cluster [Cp*LaI_2_]_9_, while the exhaustive reaction of [Cp′La(AlMe_4_)_2_] with the halogenido transfer reagents Me_3_GeX and Me_3_SiX (X=I, Br, Cl) produced a series of monocyclopentadienyl rare‐earth‐metal clusters with distinct nuclearity. Depending on the halogenido ion size the homometallic clusters [Cp′LaCl_2_]_10_ and [Cp′LaX_2_]_12_ (X=Br, I) could be isolated, whereas different crystallization techniques led to the aggregation of clusters of distinct structural motifs, including the desilylated cyclopentadienyl‐bridged cluster [(μ‐Cp)_2_Cp′_8_La_8_I_14_] and the heteroaluminato derivative [Cp′_10_La_10_Br_18_(AlBr_2_Me_2_)_2_]. The use of the Cp′ ancillary ligand facilitates cluster characterization by means of NMR spectroscopy.

## Graphical abstract

**Half‐sandwich complexes** Cp^R^La(AlMe_4_)_2_ engage in selective alkyl/halogenido(X) ligand exchange reactions in non‐coordinating solvents. By scrutinizing anion‐size effects and crystallization conditions distinct coordination clusters of the type [Cp^R^LaX_2_]_*x*_ (X=I, Br, Cl) are accomplished, including nona‐, deca‐, and dodecalanthanum entities.

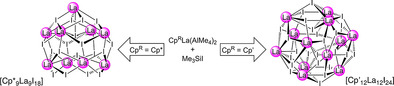

## Introduction

High‐nuclearity rare‐earth‐metal (Ln) coordination clusters not only impress by sheer beauty but have gained increasingly in significance for magnetic and optical applications.^1^, ^2^ The so far largest Ln^III^ clusters have been achieved by self‐assembly processes under hydrothermal or solvothermal conditions involving ligand (“carboxylato”)‐ and pH‐controlled, anion‐templated hydrolyses.^3^ The obtained nanoscale rare‐earth‐metal hydroxide clusters can be sub‐divided into Ln‐exclusive (e.g., {Gd_140_},^4^ {Nd_104_},^5^ {Dy_76_},^6^ {Gd_60_}^7^) and heterometallic 4f‐3d derivatives (e.g., {Dy_96_Ni_64_},^8^ {La_60_Ni_76_},^9^ {Gd_102_Ni_36_}^10^), featuring overall complicated compositions such as [Gd_140_(μ_3_‐OH)_100_(CH_3_COO)_80_(LH_3_)_40_(H_2_O)_200_](NO_3_)_80_(H_2_O)_*x*_ (*x* ≈ 80; LH_6_=myo‐inosito)^4^ or [Dy_96_Ni_64_(μ_3_‐OH)_156_(IDA)_66_(DMPA)_12_(CH_3_COO)_48_(NO_3_)_24_(H_2_O)_64_]Cl_24_ (IDA=iminodiacetate, DMPA=2,2‐dimethylol propionic acid).^8^ Self‐assembly processes in non‐aqueous media as a rule produce significantly lower nuclearities as shown for, e.g., the selenide complexes [(py)_16_Ce_17_NaSe_18_(SePh)_16_]^11^ and [(py)_24_Pr_28_F_68_(SePh)_16_],^12^ which display effective near‐IR emitters. The syntheses of the latter fluorido‐rich clusters from Ln(SePh_3_) and NH_4_F in pyridine clearly emphasize the mandatory switch to distinct reaction protocols.^12^

In 1998, the highly symmetric chlorido‐bridged [Cp_12_Sm_12_Cl_24_] emerged as a benchmark system in organolanthanide cluster chemistry.^13^, ^14^ This dodecanuclear cluster was obtained by desolvating [CpSmCl_2_(thf)_3_] under reflux and repeatedly extracting it with toluene at 80 °C.^15^ Ever since, the nuclearity of {Sm_12_} has remained unmatched for cyclopentadienyl‐based derivatives (and organometallics in general), while the routinely observed nuclearity of the involved ring and cluster motifs seems stationary at {Ln_6_}.^16^ Crucially, while metallocene complexes of the general formula Cp_2_LnX tend to form dimeric and higher‐ring structures (highly polarized Ln−X bonding, X=small electron‐withdrawing ligand),^17^, ^18^, ^19^ donor‐free half‐sandwich complexes CpLnX_2_ are prone to coordination cluster formation.^14^, ^16^ Beside [Cp_12_Sm_12_Cl_24_],^13^ the only other reported donor‐free CpLnX_2_ derivatives comprise ring‐like [Cp*_4_Sc_4_I_8_]^20^ and [Cp*_3_Dy_3_I_6_] (Cp*=C_5_Me_5_).^21^ Related mixed‐valent [Cp*_6_Yb_4_I_4_]^22^ and [Cp*_5_Sm_5_I_9_],^23^ Cp‐enriched congeners [Cp*_6_Yb_5_F_9_]^24^ and [Cp*_6_Sm_5_Cl_9_],^21^ as well as heterobimetallic [Cp_7_Dy_7_I_14_](μ‐I)[Cp_2_V]^25^ form cluster motifs. Viable synthesis protocols for the half‐sandwich coordination clusters include salt metathesis,16b, 16e, 16f, 16g hydrogenolysis,16d, 16h thermolysis/desolvation,^13^, ^26^ and Ln redox transformations.^21^, ^22^, ^23^, ^24^, ^25^, ^27^, ^28^ In the realm of Ziegler–Natta catalysts and respective model compounds, we have embarked on alkylaluminato/halogenido ligand exchange and donor‐promoted alkylaluminate cleavage of discrete half‐sandwich complexes [Cp*Ln(AlMe_4_)_2_]^29^ and gained access to {La_6_} and {Gd_8_} entities of the type [Cp*_6_La_6_Cl_8_(AlMe_4_)_4_]^30^ and [(Cp*_8_Gd_8_Me_4_(AlMe_4_)_4_(CH_2_O*t*Bu)_8_].^31^ Spurred by the ease and efficiency of the former partial alkylaluminato/halogenido ligand exchange and the dearth of data on higher‐nuclearity organolanthanide clusters, we now targeted new types of donor‐free CpLnX_2_ derivatives via complete alkyl/halogenido exchange.

## Results and Discussion

### C_5_Me_5_ (Cp*)‐supported half‐sandwich La^III^ iodide clusters

To adequately assess any partial/complete alkyl/halogenido exchange in terms of structural implications, we initially selected the iodination reaction of known [Cp*La(AlMe_4_)_2_] (**1 a**)^29^ with Me_3_SiI. Thereby, the large La^III^ center and the large I^−^ anion were anticipated to promote the formation of maximum‐nuclearity clusters.

Accordingly, complex **1 a** was treated with various amounts of Me_3_SiI. Already the equimolar reaction in unstirred *n*‐hexane solutions led to the crystallization of the hexanuclear cluster [Cp*_6_La_6_I_8_(AlMe_4_)_4_] (**2**) at ambient temperature within hours. Compound **2** incorporates one solvent molecule per cluster in the crystal lattice, which could not be removed in oil pump vacuum, and crystallized in the triclinic space group *P* $\bar{1}$ . Complex **2** has the connectivity [La_6_Cp*_6_{(μ‐Me)_3_AlMe}_4_(μ_3_‐I)_2_(μ_2_‐I)_6_] (Figure S23) and is isostructural to the previously reported chloride complex [Cp*_6_La_6_Cl_8_(AlMe_4_)_4_].^30^ Thereby, two “Cp*_3_La_3_X_4_” subunits are linked through the four tetramethylaluminato ligands in a μ_2_‐η^1^:η^2^ fashion. Each lanthanum atom is eight‐coordinated by three iodido, two methyl, and one Cp* ligand. While the La−Cp* ancillary ligand distances as well as the aluminato coordination behavior are only marginally affected by the Cl^−^/I^−^ halogenido exchange, the La−Cl/I distances differ markedly by nature (La−Cl: 2.8049(9), 2.8439(9) Å (μ_2_); 3.0060(7)–3.0708(8) Å (μ_3_) versus La−I: 3.2096(4)–3.2323(5) Å (μ_2_); 3.2327(4)–3.4366(4) Å (μ_3_)).

**Scheme 1:**
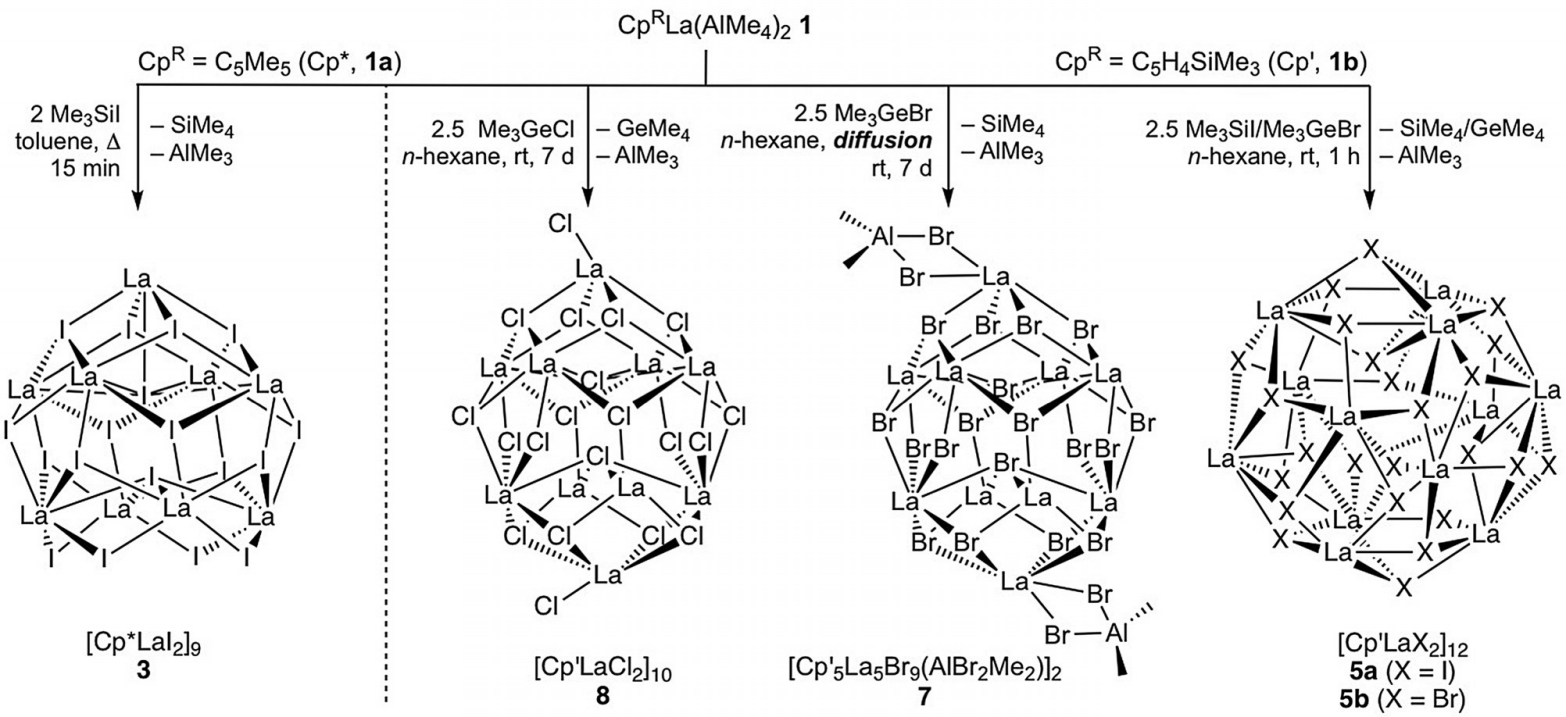
Reactivity of half‐sandwich complexes **1** toward halogenido transfer reagents applying different synthesis strategies (Cp′ and Cp* ligands are omitted for clarity).

**Figure 1:**
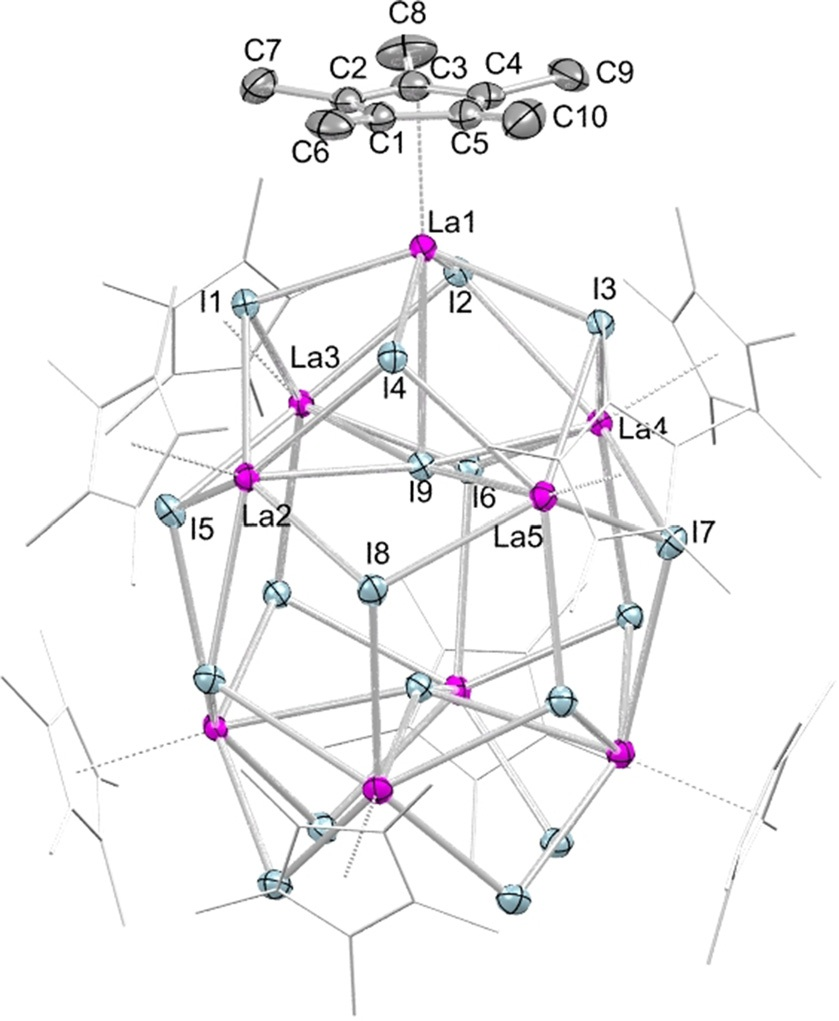
Crystal structure of **3** with atomic displacement parameters set at the 50 *%* probability level. Hydrogen atoms are omitted for clarity. The Cp* ligands (except for one) are represented by a wireframe model for improved visualization. For selected interatomic distances and angles, see the Supporting Information.

Intriguingly, treatment of **1 a** with two equivalents of Me_3_SiI afforded complete alkyl/halogenido exchange as envisaged (Scheme 1), and even more interestingly the hoped‐for cluster enlargement. Nonametallic [Cp*_9_La_9_I_18_] (**3**) is the first of its kind and its crystal structure is depicted in Figure 1. Crystalline **3** contains two independent clusters and 14 molecules of toluene in the unit cell. The large amount of solvent remained partly in the crystal lattice even when oil pump vacuum was applied. The nine lanthanum centers constitute a pseudo‐tridecahedron with each metal center shielded by a Cp* ligand. The five lanthanum centers of the square pyramidal subunit are additionally coordinated by four μ_3_‐bridging iodido anions and one central μ_5_‐I. Eight out of a total of 12 μ_3_‐bridging iodido anions connect to the four lanthanum centers of the square planar subunit. These four lanthanum centers are μ_2_‐bridged with iodido anions to build a crown‐like motif, and are further sharing a central μ_4_‐bridging iodido moiety. Complex **3** readily dissolves in THF to generate monomeric [Cp*LaI_2_(thf)_3_] (Figure S25).

The number of structurally characterized organometallic complexes containing iodido‐bridged lanthanum atoms are limited. For comparison, the La−(μ_2_‐I) distances of 3.2050(7)–3.2561(7) Å are slightly shorter than those in [La(μ_2_‐I){N(SiMe_3_)_2_}_2_(thf)]_2_ 32 (3.3096(11), 3.3051(11) Å) or [(COT)La(μ_2_‐I)(thf)_2_]_2_ 33 (3.3832(2), 3.3157(2) Å). A series of salt‐like mixed iodido/ethanido lanthanum‐based solid‐state materials including o‐La_5_I_9_(C_2_)34a and La_10_I_15_(C_2_)_2_ 34b were shown to feature La−(μ_3_‐I) (3.225(3)–3.544(2) Å) and La−(μ_4_‐I) moieties (3.1722(7)–3.3795(4) Å), which, however, appear considerably shorter than the distances of 3.1857(7)–3.6720(7) Å (μ_3_) and 3.5187(7)–3.5447(7) Å (μ_4_) in **3**. Surprisingly, organolanthanum complexes with μ_5_‐bridging iodido ligands seem even rarer. However, the La−(μ_5_‐I) distances of 3.5335(7)–3.6885(6) Å in **3** are comparable to the Sm−(μ_5_‐I) distances in octa‐ and pentametallic complexes [{[μ‐Ph_2_C(C_4_H_3_N)_2_]Sm}_5_(μ_5_‐I)]^−^[{[μ‐Ph_2_C(C_4_H_3_N)_2_]Sm(thf)}_3_(μ_3_‐I)]^+^ and [{[μ‐MePhC(C_4_H_3_N)_2_]Sm}_5_(μ_5_‐I)]^−^[K(thf)_6_]^+^, respectively, where the samarium atoms are pentagonally arranged around a coplanar iodido ion (Sm−(μ_5_‐I), 3.434(1)–3.634(1); 3.517(1)–3.5566(6) Å).^35^ For further comparison, a similar square pyramidal {Ln_5_} structural motif was also detected in donor‐free mixed‐valent complex [Cp*_5_Sm_5_(μ_2_‐I)_4_(μ_3_‐I)_4_(μ_5_‐I)] (Sm−(μ_5_‐I): 3.281(1)–3.377(1) Å),^23^ while the separated ion‐pair [Cp_6_Yb_6_Cl_13_]^−^[Cp_3_Yb_3_Cl_5_(thf)_3_]^+^ features another “nonametallic” half‐sandwich arrangement.^13^

### C_5_H_4_SiMe_3_ (Cp′)‐supported half‐sandwich La^III^ iodide clusters

Having established the proof of concept for efficient cluster enlargement in Cp* derivatives [Cp*LnX_2_]_*x*_, the next step was to investigate into the effect of the cyclopentadienyl ligand. Here, we decided upon the trimethylsilyl cyclopentadienyl (Cp′) ligand, offering two decisive advantages compared to Cp* derivatives: overall decreased steric demand (enforcing cluster enlargement) and mediation of better solubility (facilitating NMR‐spectroscopic characterization). The half‐sandwich lanthanum bis(tetramethylaluminato) precursor complex [Cp′La(AlMe_4_)_2_] (**1 b**) was straightforwardly synthesized from homoleptic La(AlMe_4_)_3_ and KCp′ following routine protocols.^36^ Rather unexpectedly, the crystal structure of **1 b** shows a symmetric tetramethylaluminato coordination with two planar La(μ‐Me)_2_Al units (in contrast to one planar and one bent one as routinely observed).^36^ As a consequence, steric saturation of the La^III^ centers in **1 b** is achieved by interaction with one aluminato methyl group of another molecule resulting in a μ_2_‐η^1^:η^2^ aluminato coordination mode. These weak intermolecular interactions (La⋅⋅⋅C 3.250 Å) imply a dimeric arrangement in the solid state (Figure S21).

**Figure 2:**
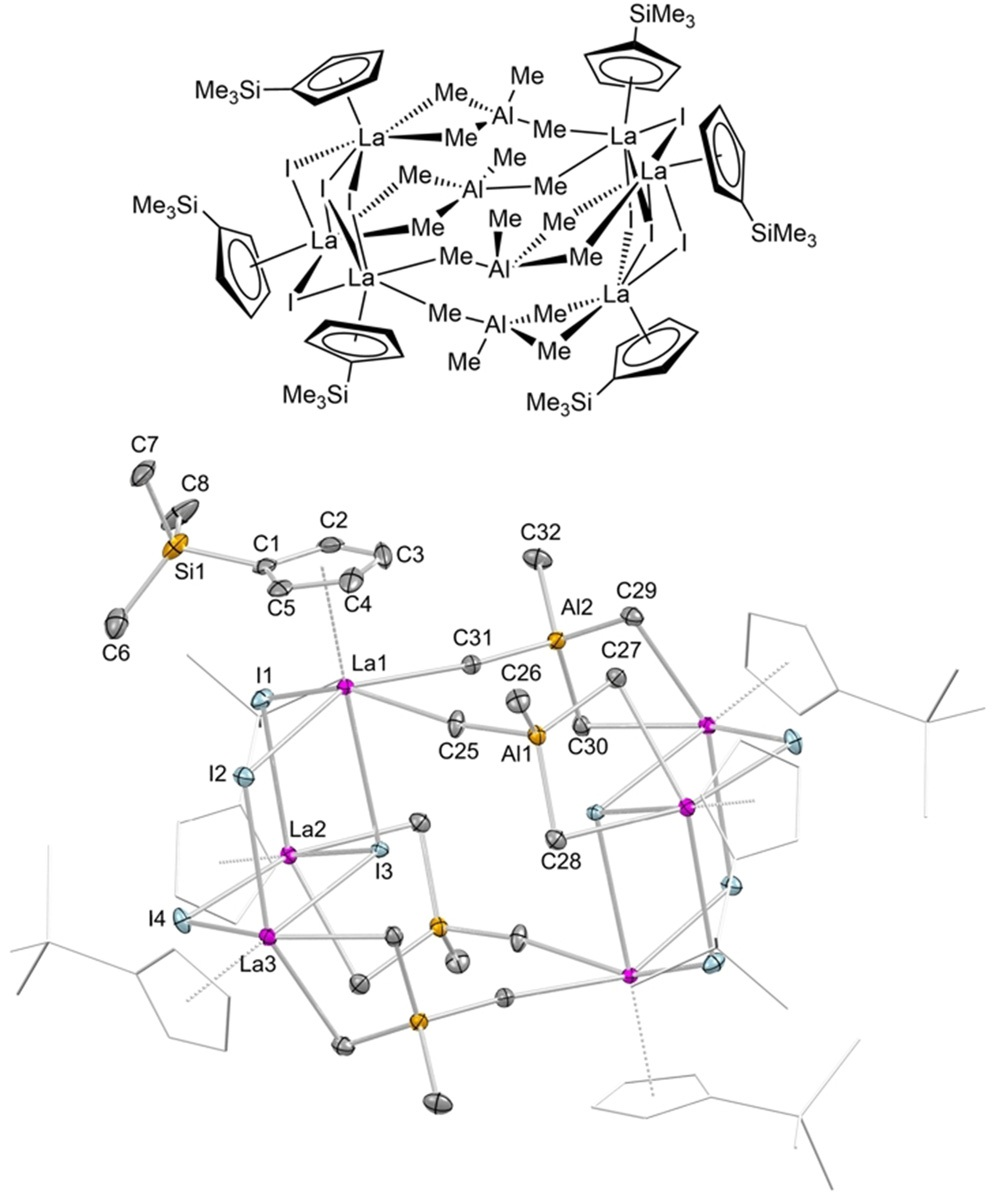
Molecular drawing (top) and crystal structure (bottom) of **4** **a** with atomic displacement parameters set at the 50 % probability level. Hydrogen atoms are omitted for clarity. The Cp′ ligands (except for one) are represented by a wireframe model for improved visualization. For selected interatomic distances and angles, see the Supporting Information.

Surprisingly, treatment of **1 b** with two equivalents of Me_3_SiI at ambient temperature in *n*‐hexane did not result in the envisaged complete alkyl/halogenido exchange, but the tetramethylaluminato‐bridged heterobimetallic hexalanthanum cluster [Cp′_6_La_6_I_8_(AlMe_4_)_4_] (**4 a**). Again surprisingly, compound **4 a** is isostructural to the Cp* derivatives [Cp*_6_La_6_Cl_8_(AlMe_4_)_4_] and **2** (Figures 2 and S23).^30^ Overall, the arrangement of the six lanthanum metal centers is reminiscent of two cuboid structures with cut‐off corners. Complexes [Cp^R^_6_La_6_X_8_(AlMe_4_)_4_] show similar metrical parameters concerning the lanthanum metal centers and the ancillary Cp^R^ moieties ([Cp*_6_La_6_Cl_8_(AlMe_4_)_4_]: 2.728(3)–2.819(4) Å; **2**: 2.748(4)–2.797(4); **4 a**: 2.711(6)–2.793(5) Å). A striking difference of the La_3_X_4_ subunits is revealed by the bond angles. More precisely, the La−Cl−La angles of 100.55(2)–113.19(4)° are more obtuse than La−I−La angles in **2** (100.126(2)–108.169(2)°) and **4 a** (99.962(2)–106.822(2)°), resulting in a maximum difference of 6.37°.

**Figure 3:**
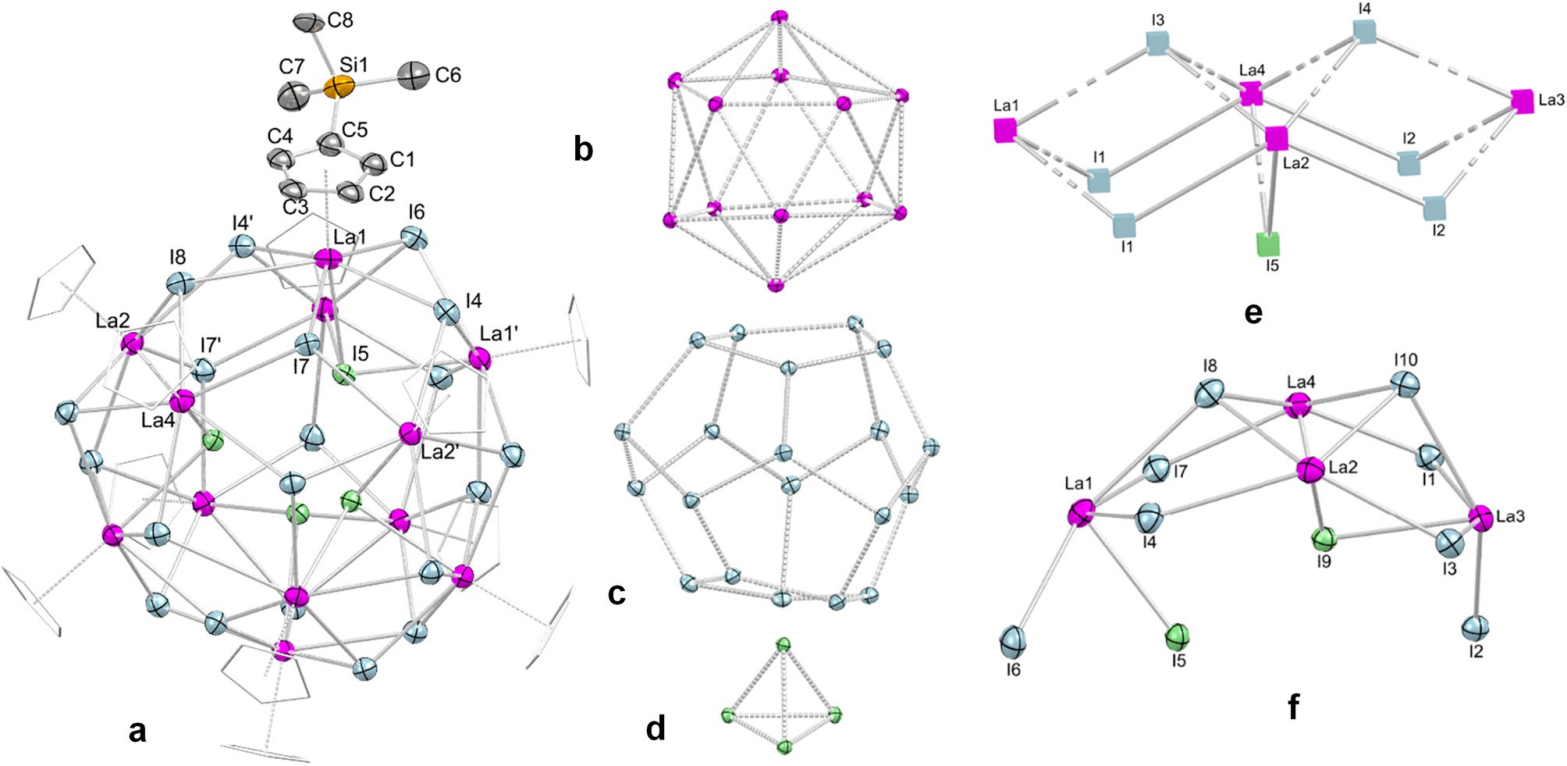
Crystal structure of **5** **a** with atomic displacement parameters set at the 50 *%* probability level. a) The entire molecule with hydrogen atoms omitted for clarity and Cp′ ligands (except for one) represented by a wireframe model for improved visualization. (b), (c), and (d) represent the La_12_, I_20_, and I_4_ polyhedra; comparison of the asymmetric unit (f) with the solid‐state‐structure of LaI_3_ ((e), adapted from Zachariasen).^38^ For selected interatomic distances and angles, see the Supporting Information.

Crucially, complete AlMe_4_/halogenido exchange could be accomplished by the addition of a slight excess of Me_3_SiI (2.5 equiv.) to **1 b** under vigorous stirring (Scheme 1), as confirmed by the isolation of the dodecalanthanum cluster [Cp′LaI_2_]_12_ (**5 a**, 48 % yield). XRD analysis of **5 a** revealed a La_12_ icosahedron, with each of the 20 faces capped by one iodido ligand, as depicted in Figure 3 (space group *R*3*c*). The iodido ligands arrange in a dodecahedron with one additional I_4_ tetrahedron located inside the cluster core. Complex **5 a** is isostructural to the until‐now unique dodecasamarium cluster [CpSmCl_2_]_12_, reported by Kretschmer et al. in 1998 (space group *I*4_1_/*acd*).^13^ For comparison, the Sm−Cl bond lengths at the cluster surface in [CpSmCl_2_]_12_ are in the range of 2.70(1) to 3.10(2) Å (av. 2.99 Å) and average 2.95 Å inside the cluster core. In contrast, the respective La−I bond lengths in the cluster periphery of **5 a** average 3.3625 Å, being therefore considerably longer than inside the cluster core (av. La−I 3.2808 Å). Taking into account the ionic radii (Sm^3+^+ Cl^−^=2.77 Å; La^3+^+ I^−^=3.19 Å),^37^ the average interatomic distances in {Sm_12_} and {La_12_} (**5 a**) are noticeably larger. However, the overall bonding situation in these clusters is almost identical, differing marginally by only 0.021 Å. The asymmetric unit of **5 a** shows a distinct La_4_I_7_ structural motif, which can be considered a distorted cutout of the LaI_3_ solid‐state structure,^38^ indicating the general stability of the cluster (Figure S28).

The differences in the solid‐state structures of **5 a** (formally 9‐coordinate La centers) and LaI_3_ (8‐coordinate La centers) are evidenced by the bent arrangement of the asymmetric unit in **5 a**. More precisely, the La−I distances of 3.211(2), 3.267(2) and 3.287(2) Å (La1−I4/I7, La4−I7) in **5 a** are slightly shorter than in LaI_3_ (3.342 Å), while the La2−I1 bond length of 3.585(2) Å in **2** is considerably longer compared to LaI_3_ (3.396 Å). The bending of the asymmetric unit of **5 a** is further evidenced by marked differences in the interatomic distances of the central iodido ligands to La1 and La3 (La⋅⋅⋅I9: **5 a**, 5.152(2), 3.303(2) Å), which is significantly shorter than in LaI_3_ (5.638 Å). This results in a strongly bent arrangement of the La_4_I_7_ fragment in accord with the obtuse I−La−I angles in **5 a** (I4−La1−I7=122.95(5)°, I1−La3−I3=146.00(5)°), while in lanthanum iodide the respective angle is 80.97°. This arrangement is clearly affected by the shielding and the steric effect of the ancillary Cp′ ligands. Comparison may be also drawn to the similarly sized metalloid aluminum cluster [Al_50_Cp*_12_] (diameter: 14.9 Å, **5 a**: 14.6 Å), the topology of the 60 methyl groups of which bearing a close resemblance to that of fullerene C_60_.^39^ Indeed, Kretschmer et al. had also pointed out that the truncated icosahedron adopted by the 60 carbon atoms of 12 Cp ligand in [CpSmCl_2_]_12_ is “somewhat analogous” to fullerene C_60_.^13^

In contrast to the hardly soluble (in non‐donor solvents) Cp*‐derived clusters **2** and **3**,^30^ the new Cp′‐supported clusters **4 a** and **5 a** dissolve readily in benzene. However, the ^1^H NMR spectrum of methylaluminate/iodide cluster **4 a** in [D_6_]benzene at ambient temperature (Figure S5) revealed its fragmentation in solution, which is indicated by the appearance of additional resonances for **1 b** and **5 a**. Moreover, the triplet resonances at 6.78 and 6.66 ppm (^3^ *J*_H,H_=2.5 Hz) and the multiplets at 6.74 and 6.63 ppm indicate non‐equivalent Cp′ ligands, which is corroborated by signals at 0.41 and 0.37 ppm for the SiMe_3_ groups. The singlet at −0.03 ppm can be assigned to the La‐[AlMe_4_] moieties of **4 a**, indicating a rapid exchange of bridging and terminal methyl groups. The ^1^H NMR spectrum of dodecalanthanum cluster [Cp′LaI_2_]_12_ (**5 a**) in [D_6_]benzene shows one signal set for the Cp′ ligands, indicating dynamic processes of **4 a** in solution. The Cp′ hydrogen atoms at the positions 3/4 and 2/5 resonate at 7.20 ppm and 7.00 ppm, respectively, and the trimethylsilyl group at 0.61 ppm, significantly shifted to lower field in comparison to the corresponding signals of precursor **1 b** (*δ* Cp′*H*, 6.40 ppm, 6.22 ppm, δ C*H_3_*, 0.14 ppm). Moreover, a second set of signals with the same 6:1 integral ratio of singlet and triplets revealed the formation of another similar species in solution. Therefore, a three‐step process for the formation of cluster **5 a** seems plausible: initial complete [AlMe_4_]/I ligand exchange, followed by aggregation to Cp′_4_La_4_I_8_ fragments, and subsequent self‐assembly of three such La_4_ units to afford the large Cp′_12_La_12_I_24_ entity.

**Figure 4:**
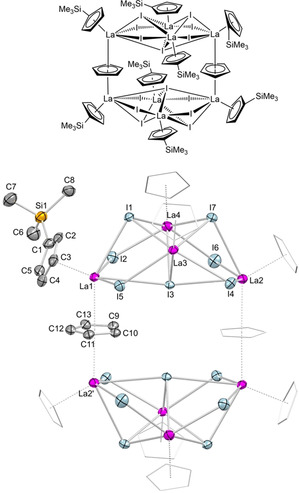
Molecular drawing (top) and crystal structure (bottom) of **6** with atomic displacement parameters set at the 50 % probability level. Hydrogen atoms are omitted for clarity. The Cp′ ligands (except for one) are represented by a wireframe model and without SiMe_3_ groups for improved visualization. For selected interatomic distances and angles, see the Supporting Information.

Supportive of such a mechanism of formation is the isolation and structural characterization of the mixed‐Cp octalanthanum species [(μ‐Cp)_2_Cp′_8_La_8_I_14_] (**6**). Applying slow diffusion of Me_3_SiI (2.5 equiv., dissolved in *n*‐hexane) into an *n*‐hexane solution of **1 b** (instead of vigorous stirring) and subsequent crystallization at −40 °C afforded compound **6** in low yield (Figure 4 and Figure S29). An XRD analysis revealed that cluster **6** is composed of two Cp′_4_La_4_I_7_ subunits with two eight‐ and nine‐coordinate lanthanum centers each (4× μ_2_‐I, 2× μ_3_‐I, 1× μ_4_‐I), thus resembling the asymmetric unit of cluster **5 a** (Figure 3). The nine‐coordinate lanthanum centers at the vertices of the La1La2La1′La2′ planar rectangular arrangement are μ_2_:η^5^,η^5^‐interconnected by unsubstituted cyclopentadienyl ligands in an inverse‐sandwich‐type fashion. The steric demand of the bridging Cp ligands effects the Cp′_4_La_4_I_7_ subunits, resulting in an overall high symmetry in comparison to the asymmetric unit of **5 a**.

In complex **5 a**, the outmost La−I bonds of the heavily distorted asymmetric unit are markedly elongated (La2−I4, 3.585(2) Å) to adapt to the stable La_12_ structure, while the respective bonds in **6** are in the range of 3.186(1) Å (La4−I6) to 3.292(2) Å (La2−I6). Moreover, the subunit of **6** shows a reduced interatomic distance between the outer lanthanum metal center and the central iodido ligand (La1−I3=3.734(1) Å; **5 a**: La1⋅⋅⋅I9=5.152(2) Å), which might be due to the strain induced by the bridging Cp ligands.

^1^H NMR spectroscopic studies corroborate the displacement of the trimethylsilyl groups from the respective Cp′ ligands in **6** (formation of Si_2_Me_6_ evidenced for three independent samples, Figure S11), subsequently enabling the bridging of the La_4_I_7_ fragments. It must be noted that the “Wanderlust” of trimethylsilyl groups has been recently emphasized in rare‐earth metal triple‐decker sandwich complexes of the type Ln_2_(COT′′)_3_ (COT′′=bis(trimethylsilyl) cyclooctatetraenyl).^40^ The ease of SiMe_3_ dissociation in HCp′ is also reflected in the synthesis of the precursor of cluster [CpSmCl_2_]_12_ by dehalosilylation employing SmCl_3_.^13^ The ^1^H NMR spectrum of **6** shows the presence of side products, while the protons at the 2/5 positions and 3/4 positions of the Cp′ ligand resonate at 7.19 and 7.00 ppm, respectively, and overlap with the respective signals of **5 a** (minor product). The signals in the range of 1.64 to 0.92 ppm are likely generated by a solution side product, and the Me_3_Si groups resonate at 0.61 ppm. It must be noted that the Cp′ resonances are shifted to higher field compared to precursor **1 b**. Overall, the formation of the lanthanum iodide clusters **5 a** and **6** is mainly depending on the crystallization technique, indicating a strong kinetic control of the alkylaluminato/halogenido ligand exchange reaction. Complex **5 a** readily dissolves in THF to generate monomeric [Cp′LaI_2_(thf)_3_] (Figure S22). Complexes **4 a**, **5 a** and **6** were further analyzed by SEM and EDX to check product purity via elemental ratios (Figures S41–S48, Tables S3–S14).

### C_5_H_4_SiMe_3_ (Cp′)‐supported half‐sandwich La^III^ bromide and chloride clusters

Changing the halogenido transfer reagent to Me_3_GeBr while applying otherwise identical reaction protocols, the respective bromido‐bridged clusters [Cp′_6_La_6_Br_8_(AlMe_4_)_4_] (**4 b**) and [Cp′LaBr_2_]_12_ (**5 b)** could be isolated. Accordingly, treatment of complex **1 b** with 1.3 equiv. of Me_3_GeBr afforded the heterobimetallic hexalanthanum cluster **4 b** (Figure S30), which is isostructural to [Cp*_6_La_6_Cl_8_(AlMe_4_)_4_],^30^ **2**, and **4 a**. Compound **4 b** shows degradation behavior similar to **4 a**, as indicated by NMR spectroscopy (Figures S12 and S13). The dodecalanthanum cluster **5 b** could be selectively obtained by treatment of **1 b** with 2.5 equiv. Me_3_GeBr in a vigorously stirred *n*‐hexane solution at ambient temperature (Scheme 1, 47 % yield). The structural characterization of **5 b** by XRD analysis (Figure S31) revealed the same structural motif as for the respective iodido‐bridged cluster **5 a**, with the La−Br distances following the same trend. The ^1^H NMR spectrum of **5 b** shows the expected set of signals for the Cp′ ligand, indicating a highly symmetric coordination. In comparison to **5 a** the signals are shifted to higher field.

**Figure 5:**
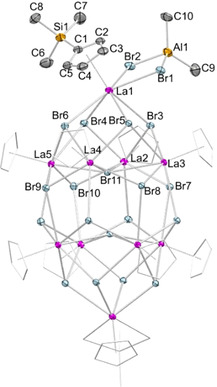
Crystal structure of **7** with atomic displacement parameters set at the 50 % probability level. Hydrogen atoms are omitted for clarity. The Cp′ ligands (except for one) are represented by a wireframe model for improved visualization. For selected interatomic distances and angles, see the Supporting Information.

Interestingly, a divergent reactivity was observed when applying the diffusion protocol. Thereby, the bromido transfer gave the decalanthanum cluster [Cp′_10_La_10_Br_18_(AlBr_2_Me_2_)_2_] (**7**) featuring two heteroaluminato ligands (Scheme 1). The XRD analysis of **7** revealed an ellipsoidal structural motif involving eight lanthanum atoms, being terminated by two additional peripheral lanthanum metal centers (Figure 5 and Figure S32). The lanthanum centers at these apical positions coordinate to a η^5^‐Cp′ ligand, a AlBr_2_Me_2_ moiety, and cap a La_4_Br_4_ crown. The four lanthanum centers of the latter crown interact further with a central bromido ligand and another eight bromido ligands, which bridge to the other four lanthanum centers of the second La_4_Br_4_ crown. Overall, the eight inner lanthanum centers are connected to a total of 16×μ_3_‐I and 2×μ_4_‐I, as well as 8×η^5^‐Cp′. The terminal heteroaluminato ligands [(μ_2_‐Br)_2_AlMe_2_] in complex **7** coordinate symmetrically in a η^2^ fashion to the lanthanum metal centers (La1−Br1=3.0945(4) Å, La1−Br2=3.0595(5) Å), involving La⋅⋅⋅Al distances of av. 4.0745 Å which are significantly longer than in complex **1 b** (av. La⋅⋅⋅Al=3.2678 Å). Similar alkyl/chlorido heteroaluminato moieties were previously reported for metallocene complexes of the type [Cp*_2_Ln(μ‐Cl)_2_AlR_2_] (Ln=Y, Sm; R=Me, Et, *i*Bu).^41^ The La−Br bond lengths of the apical 11‐coordinate lanthanum to the La_4_Br_4_ crown are in the range of 3.1450(4) to 3.3167(4) Å, and hence longer than in lanthanum bromide LaBr_3_ (9‐coordinate La^III^, 3.101 to 3.158 Å).^42^

For further comparison, the La−(μ_4_‐Br) distances in **7** are similar, ranging from 3.2244(4) to 3.2758(4) Å. The ^1^H NMR spectrum of compound **7** indicates the presence of dodecalanthanum cluster **5 b** along with another minor co‐product. The separation of **5 b** and **7**, however, is hampered by similar crystal morphology and solubility (Figure S16). The trimethylsilyl groups of the Cp′ ligands of compound **7** resonate in the range of 0.64 to 0.42 ppm in accordance with non‐equivalent positions. The heteroaluminato ligands [AlMe_2_Br_2_] show one broad singlet at 0.33 ppm. EDX analyses for compounds **4 b**, **5 b**, and **7** were impeded by the overlap of the element signals of Al and Br, making a meaningful quantification of these elements difficult. Nevertheless, the atom ratios of lanthanum are within the expected range, further supporting the formation and purity of the respective clusters (Figures S49–S57, Tables S15–S26).

Using Me_3_GeCl as the halogenido transfer reagent, the exclusive formation of one species was observed, regardless of the synthesis methods applied. Treatment of **1 b** with Me_3_GeCl resulted in the formation of the decalanthanum cluster [Cp′LaCl_2_]_10_ (**8**), featuring a similar structural motif as bromide cluster **7**. Instead of the terminal AlMe_2_Br_2_ ligands in complex **7**, the lanthanum metal centers in the apical positions of **8** bear terminal chlorido ligands. The connectivity of **8** was elucidated by XRD analysis (Figure S33). The ^1^H NMR spectrum of **8** shows the expected set of signals for the Cp′ ligand as well as signals of a solution side product. In comparison to complexes **5 a** and **5 b**, the signals are shifted to lower field. The formation of **8** is further confirmed by an EDX analysis, which revealed the expected elemental ratios (Figures S58–S60, Tables S27–S30).

## Conclusions

Half‐sandwich complexes [Cp^R^La(AlMe_4_)_2_] engage in selective alkyl/halogenido(X) ligand exchange reactions in non‐coordinating solvents under mild conditions. The fully exchanged donor solvent‐free [Cp^R^LaX_2_] self‐assemble to form distinct nanoscale coordination clusters. The size of the homometallic clusters depends on the steric demand of the cyclopentadienyl and halogenido ligands as revealed for [Cp*_9_La_9_I_18_], [Cp′_10_La_10_Cl_20_]_,_ and [Cp′_12_La_12_X_24_] (X=Br, I). Moreover, the cluster formation/crystallization procedure is shown to affect the cluster composition, and more crucially, the use of the trimethylsilyl‐substituted cyclopentadienyl ligand (Cp′) implies high cluster solubility and hence characterization by means of solution NMR spectroscopy. The isolation of such nanoscale clusters proves that the fascinating icosahedral arrangement of samarium centers detected ca. 20 years ago in [Cp_12_Sm_12_Cl_24_]^13^ is not a one‐off occurrence in rare‐earth metal chemistry. It can be anticipated that the present nanochemistry approach, which utilizes highly reactive organometallics and mild halogen‐transfer reagents such as Me_3_SiI or Me_3_GeX (X=Br, Cl), will decisively promote the field of nanoscale rare‐earth‐metal cluster research. The findings might also stimulate similar research with paramagnetic rare‐earth metals opening new avenues for the design of luminescent and magnetic clusters and materials.^2^

## Experimental Section

### Crystallographic data

Deposition Numbers 1992037, 1992038, 1992039, 1992040, 1992041, 1992042, 1992043, 1992044, 1992045, 1992046, 1992047, and 1992048 contain the supplementary crystallographic data for this paper. These data are provided free of charge by the joint Cambridge Crystallographic Data Centre and Fachinformationszentrum Karlsruhe Access Structures service.

## Conflict of interest

The authors declare no conflict of interest.

## Supporting information

As a service to our authors and readers, this journal provides supporting information supplied by the authors. Such materials are peer reviewed and may be re‐organized for online delivery, but are not copy‐edited or typeset. Technical support issues arising from supporting information (other than missing files) should be addressed to the authors.

Supplementary

## References

[1a] X.-Y. Zheng , X.-J. Kong , Z. Zheng , L.-S. Long , L.-S. Zheng , Acc. Chem. Res. 2018, 51, 517–525; 29393625 10.1021/acs.accounts.7b00579

[1b] X.-Y. Zheng , J. Xie , X.-J. Kong , L.-S. Long , L.-S. Zheng , Coord. Chem. Rev. 2019, 378, 222–236.

[2a] F. Habib , M. Murugesu , Chem. Soc. Rev. 2013, 42, 3278–3288; 23334210 10.1039/c2cs35361j

[2b] D. N. Woodruff , R. E. P. Winpenny , R. A. Layfield , Chem. Rev. 2013, 113, 5110–5148; 23550940 10.1021/cr400018q

[2c] B. M. Day , F.-S. Guo , R. A. Layfield , Acc. Chem. Res. 2018, 51, 1880–1889. 30091896 10.1021/acs.accounts.8b00270

[3] G. A. Kumar , R. E. Riman , J. G. Brennan , Coord. Chem. Rev. 2014, 273–274, 111–124.

[4] X.-Y. Zheng , J.-H. Jiang , G.-L. Zhuang , D.-P. Liu , H.-G. Liao , X.-J. Kong , L.-S. Long , L.-S. Zheng , J. Am. Chem. Soc. 2017, 139, 18178–18181. 29200280 10.1021/jacs.7b11112

[5] J.-B. Peng , X.-J. Kong , Q.-C. Zhang , M. Orendác , J. Prokleska , Y.-P. Ren , L.-S. Long , Z. Zheng , L.-S. Zheng , J. Am. Chem. Soc. 2014, 136, 17938–17941. 25495563 10.1021/ja5107749

[6] X.-Y. Li , H.-F. Su , Q.-W. Li , R. Feng , H.-Y. Bai , H.-Y. Chen , J. Xu , X.-H. Bu , Angew. Chem. Int. Ed. 2019, 58, 10184–10188; 10.1002/anie.201903817 31090998

[7] L. Qin , G.-J. Zhuo , Y.-Z. Yu , H. Nojiri , C. Schöder , R. E. P. Winpenny , Y.-Z. Zheng , J. Am. Chem. Soc. 2017, 139, 16405–16411. 29037028 10.1021/jacs.7b09996

[8] W.-P. Chen , P.-Q. Liao , Y. Yu , Z. Zheng , X.-M. Chen , Y.-Z. Zheng , Angew. Chem. Int. Ed. 2016, 55, 9375–9379; 10.1002/anie.201603907 27345594

[9] X.-J. Kong , L.-S. Long , R.-B. Huang , L.-S. Zheng , T. D. Harris , Z. Zheng , Chem. Commun. 2009, 4354–4356. 10.1039/b822609a 19597590

[10] W.-P. Chen , P.-Q. Liao , P.-B. Jin , L. Zhang , B.-K. Ling , S.-C. Wang , Y.-T. Chan , X.-M. Chen , Y.-Z. Zheng , J. Am. Chem. Soc. 2020, 142, 4663–4670. 32033517 10.1021/jacs.9b11543

[11] B. F. Moore , G. A. Kumar , M.-C. Tan , J. Kohl , R. E. Riman , M. G. Brik , T. J. Emge , J. G. Brennan , J. Am. Chem. Soc. 2011, 133, 373–378. 21142152 10.1021/ja1069322

[12] M. Romanelli , G. A. Kumar , T. J. Emge , R. E. Riman , J. G. Brennan , Angew. Chem. Int. Ed. 2008, 47, 6049–6051; 10.1002/anie.200801530 18604794

[13] W. P. Kretschmer , J. H. Teuben , S. I. Troyanov , Angew. Chem. Int. Ed. 1998, 37, 88–90;

[14] R. Anwander , Angew. Chem. Int. Ed. 1998, 37, 599–602; 10.1002/(SICI)1521-3773(19980316)37:5<599::AID-ANIE599>3.0.CO;2-1 29711085

[15] [Cp_12_Sm_12_Cl_24_] was obtained in 35 % yield and any analytical and spectroscopic data were not provided.

[16] For examples, see;

[16a] J. Sieler , A. Simon , K. Peters , R. Taube , M. Geitner , J. Organomet. Chem. 1989, 362, 297–303;

[16b] W. J. Evans , G. W. Rabe , M. A. Ansari , J. W. Ziller , Angew. Chem. Int. Ed. Engl. 1994, 33, 2110–2111;

[16c] S. P. Constantine , G. M. De Lima , P. B. Hitchcock , J. M. Keates , G. A. Lawless , Chem. Commun. 1996, 2421–2422;

[16d] Z. Hou , Y. Zhang , O. Tardif , Y. Wakatsuki , J. Am. Chem. Soc. 2001, 123, 9216–9217; 11552852 10.1021/ja010555+

[16e] F. Bonnet , M. Visseaux , D. Barbier-Baudry , A. Hafid , E. Vigier , M. Kubicki , Inorg. Chem. 2004, 43, 3682–3690; 15180423 10.1021/ic035444l

[16f] M. D. Walter , F. Weber , G. Wolmershäuser , H. Sitzmann , Angew. Chem. Int. Ed. 2006, 45, 1903–1905; 10.1002/anie.200503986 16493718

[16g] V. Lorenz , A. Edelmann , S. Blaurock , F. Freise , F. T. Edelmann , Organometallics 2007, 26, 4708–4710;

[16h] J. Cheng , K. Saliu , G. Y. Kiel , M. J. Ferguson , R. McDonald , J. Takats , Angew. Chem. Int. Ed. 2008, 47, 4910–4913; 10.1002/anie.200705977 18496799

[17] W. J. Evans , S. E. Foster , J. Organomet. Chem. 1992, 433, 79–94.

[18] N. S. Radu , F. J. Hollander , T. D. Tilley , A. L. Rheingold , Chem. Commun. 1996, 2459–2460.

[19] K. N. Raymond , C. W. Eigenbrot, Jr. , Acc. Chem. Res. 1980, 13, 276–283.

[20] K. A. Tupper , T. D. Tilley , J. Organomet. Chem. 2005, 690, 1689–1698.

[21] W. J. Evans , T. M. Champagne , B. L. Davis , N. T. Allen , G. W. Nyce , M. A. Johnston , Y.-C. Lin , A. Khvostov , J. W. Ziller , J. Coord. Chem. 2006, 59, 1069–1087.

[22] C. J. Burns , D. J. Berg , R. A. Andersen , J. Chem. Soc. Chem. Commun. 1987, 272–273.

[23] A. M. Bienfait , B. M. Wolf , K. W. Törnroos , R. Anwander , Organometallics 2016, 35, 3743–3750.

[24] P. L. Watson , T. H. Tulip , I. Williams , Organometallics 1990, 9, 1999–2009.

[25] M. E. Burin , M. V. Smirnova , G. K. Fukin , E. V. Baranov , M. N. Bochkarev , Eur. J. Inorg. Chem. 2006, 351–356.

[26] C. O. Hollfelder , L. N. Jende , H. M. Dietrich , K. Eichele , C. Maichle-Mössmer , R. Anwander , Chem. Eur. J. 2019, 25, 7298–7302. 30945775 10.1002/chem.201901269

[27] M. T. Dumas , G. P. Chen , J. Y. Hu , M. A. Nascimento , J. M. Rawson , J. W. Ziller , F. Furche , W. J. Evans , J. Organomet. Chem. 2017, 849, 38–47.

[28] C. Schoo , S. Bestgen , A. Egeberg , J. Seibert , S. N. Konchenko , C. Feldmann , P. W. Roesky , Angew. Chem. Int. Ed. 2019, 58, 4386–4389; 10.1002/anie.201813370 30614173

[29] H. M. Dietrich , C. Zapilko , K. W. Törnroos , R. Anwander , Organometallics 2005, 24, 5767–5771.

[30] H. M. Dietrich , O. Schuster , K. W. Törnroos , R. Anwander , Angew. Chem. Int. Ed. 2006, 45, 4858–4863; 10.1002/anie.200600905 16832809

[31] C. O. Hollfelder , M. Meermann-Zimmermann , G. Spiridopoulos , D. Werner , K. W. Törnroos , C. Maichle-Mössmer , R. Anwander , Molecules 2019, 24, 3703. 10.3390/molecules24203703 PMC6832758 31618971

[32] J. Collin , N. Giuseppone , N. Jaber , A. Domingos , L. Maria , I. Santos , J. Organomet. Chem. 2001, 628, 271–274.

[33] C. Meermann , K. Ohno , K. W. Törnroos , K. Mashima , R. Anwander , Eur. J. Inorg. Chem. 2009, 76–85.

[34a] H. Mattausch , C. Hoch , A. Simon , Z. Anorg. Allg. Chem. 2008, 634, 641–645;

[34b] H. Mattausch , C. Hoch , A. Simon , Z. Anorg. Allg. Chem. 2005, 631, 1423–1429.

[35a] T. Dubé , S. Conoci , S. Gambarotta , G. P. A. Yap , G. Vasapollo , Angew. Chem. Int. Ed. 1999, 38, 3657–3659; 10.1002/(sici)1521-3773(19991216)38:24<3657::aid-anie3657>3.0.co;2-a 10649314

[35b] T. Dubé , S. Conoci , S. Gambarotta , G. P. A. Yap , Organometallics 2000, 19, 1182–1185.

[36a] M. Zimmermann , N. Å. Frøystein , A. Fischbach , P. Sirsch , H. M. Dietrich , K. W. Törnroos , E. Herdtweck , R. Anwander , Chem. Eur. J. 2007, 13, 8784–8800; 17654457 10.1002/chem.200700534

[36b] G. Occhipinti , C. Meermann , H. M. Dietrich , R. Litlabø , F. Auras , K. W. Törnroos , C. Maichle-Mössmer , V. R. Jensen , R. Anwander , J. Am. Chem. Soc. 2011, 133, 6323–6337. 21466201 10.1021/ja2001049

[37] R. Shannon , Acta Crystallogr. Sect. A 1976, 32, 751–767.

[38] W. H. Zachariasen , Acta Crystallogr. 1948, 1, 265–268.

[39] J. Vollet , J. R. Hartig , H. Schnöckel , Angew. Chem. Int. Ed. 2004, 43, 3186–3189; 10.1002/anie.200453754 15199573

[40] V. Lorenz , P. Liebing , A. Bathelier , F. Engelhardt , L. Maron , L. Hilfert , S. Busse , F. T. Edelmann , Chem. Commun. 2018, 54, 10280–10283. 10.1039/c8cc05317k 30152513

[41] W. J. Evans , T. M. Champagne , D. G. Giarikos , J. W. Ziller , Organometallics 2005, 24, 570–579.

[42] K. Krämer , T. Schleid , M. Schulze , W. Urland , G. Meyer , Z. Anorg. Allg. Chem. 1989, 575, 61–70.

