# Supplementary material for: Nanoscale Organolanthanum Clusters: Nuclearity‐Directing Role of Cyclopentadienyl and Halogenido Ligands
Source: Chemistry. 2020 Jul 27;26(47):10834–40. doi: 10.1002/chem.202001482 (PMC7496188; doi:10.1002/chem.202001482)
Supplement: Supplementary file 1 — Supplementary [file CHEM-26-10834-s001.pdf]

# Chemistry–A European Journal

Supporting Information


## Nanoscale Organolanthanum Clusters: Nuclearity-Directing Role of Cyclopentadienyl and Halogenido Ligands


Dennis A. Buschmann,[a] H. Martin Dietrich,[a] David Schneider,[a] Verena M. Birkelbach,[a] Christoph Stuhl,[a] Karl W. Törnroos,[b] Cäcilia Maichle-Mössmer,[a] and Reiner Anwander*[a]

# Table of Contents


| | |
|---|---|
| Experimental Section | S3 |
| NMR Spectroscopy | S8 |
| Crystallography | S18 |
| IR Spectroscopy | S36 |
| EDX Measurements | S40 |
| References | S60 |

# Experimental Section

**General Considerations.** All manipulations were performed under rigorous exclusion of air and moisture using standard Schlenk and glovebox techniques (MBraun MB200B; <0.1 ppm $O_2$, <0.1 ppm $H_2O$, argon atmosphere). The solvents *n*-hexane and toluene were purified using Grubbs-type columns (MBraun SPS, solvent purification system). [$D_6$]benzene (99.6%, Sigma-Aldrich) and [$D_8$]toluene (99.6%, Sigma-Aldrich) were dried over Na/K-alloy for at least 24 h and subsequently filtrated. All solvents were stored inside a glovebox. Pentamethylcyclopentadiene (98%) and trimethylsilyl cyclopentadiene (HCp', 97%) was obtained from abcr and used as received. Potassium trimethylsilyl cyclopentadienide was prepared according to standard procedures by treatment of the proligand with potassium.[2] Iodotrimethylsilane (97%, stabilized with copper), bromotrimethylsilane (98%), bromotrimethylgermane (98%), chlorotrimethylsilane (99%) and chlorotrimethylgermane (98%) were purchased from abcr and used without further purification. Homoleptic [La($AlMe_4$)$_3$][2] and half-sandwich complex [Cp*La($AlMe_4$)$_2$] (**1a**)[3] were synthesized according to literature procedures. NMR spectra of air and moisture sensitive compounds were recorded by using J. Young valve NMR tubes at ambient temperature on a Bruker AVII+400 ($^1$H: 400.11 MHz; $^{13}$C: 100.61 MHz) spectrometer. NMR chemical shifts are referenced to internal solvent resonances and reported in parts per million relative to tetramethylsilane. Coupling constants are given in Hertz. Elemental analyses were performed on an Elementar Vario Micro Cube. IR spectra were recorded on a NICOLET 6700 FTIR spectrometer with a DRIFT cell (KBr window, Kubelka-Munk conversion). EDX spectra were recorded on a HITACHI SU8030 scanning electron microscope.

**Cp'La($AlMe_4$)$_2$ (1b).** To a suspension of potassium trimethylsilyl cyclopentadiene ($KC_5H_4SiMe_3$ = KCp') (89.95 mg, 0.51 mmol) in toluene (5 mL) a solution of [La($AlMe_4$)$_3$] (200.14 mg, 0.5 mmol) in *n*-hexane (5 mL) was added under vigorous stirring. After stirring the reaction mixture for 4 h at ambient temperature, the suspension was centrifuged, the supernatant filtered, and the solvent was removed *in vacuo* to give **1b** as an off-white oil. Crystalline **1b** was obtained from a saturated *n*-hexane solution at -40 °C (216 mg, 0.48 mmol, 92%). $^1$H NMR (400 MHz, [$D_6$]benzene, 26 °C): δ = 6.41 (t, $^3J_{H,H}$ = 2.5 Hz, 2H, 3/4 Cp*H*), 6.23 (t, $^3J_{H,H}$ = 2.5 Hz, 2H, 2/5 Cp*H*), 0.15 (s, 9H, Si*Me*$_3$), -0.27 (s, 24H, Al*Me*$_4$) ppm. $^{13}$C{$^1$H} NMR (101 MHz, [$D_6$]benzene, 26 °C): δ = 129.4 (*Cp*), 124.8 (*Cp*), 121.5 (*Cp*), 2.2 (bs, Al*Me*$_4$), 0.3 (Si*Me*$_3$) ppm. IR (DRIFT): $\tilde{\upsilon}$ = 3080 (vw), 2950 (m), 2918 (s), 2890 (s), 2848 (m), 2817 (w), 2780 (w), 2021 (vw), 1763 (vw), 1705 (vw), 1671 (vw), 1610 (vw), 1442 (w), 1416 (w), 1365 (w), 1310 (vw), 1248 (s), 1200 (s), 1184 (s), 1171 (s), 1043 (vs), 901 (m), 838 (vs), 803 (vs), 755 (m), 706 (vs), 639 (m), 626

(m), 589 (m), 573 (m), 511 (m), 468 (vw), 419 (m), 405 (vw) $cm^{-1}$. Elemental analysis of **1b** calculated for $C_{16}H_{37}Al_2LaSi$ (450.41 g/mol): C 42.67%, H 8.28%; found: C 43.14%, 8.24%.

**[Cp'LaI$_2$(thf)$_3$].** [Cp'La(AlMe$_4$)$_2$] (**1b**, 11.26 mg, 0.025 mmol) was dissolved in toluene (2 mL), $Me_3SiI$ (10.01 mg, 0.05 mmol) was added, and the vial was shaken vigorously. After 5 min, 5 ml THF was added to the solution, and the vial was stored at -40 °C for 16 h. Afterwards, the solvent was removed *in vacuo*. Crystalline [Cp'LaI$_2$(thf)$_3$] was obtained from a saturated *n*-hexane/THF solution at -40 °C (15.37 mg, 0.021 mmol, 82%). Residual adduct AlMe$_3$(thf) was removed by triturating the crystalline material with *n*-hexane. $^1$H NMR (400 MHz, [D$_8$]thf, 26 °C): δ = 6.64 (t, $^3J_{H,H}$ = 2.5 Hz, 2H, 3/4 Cp*H*), 6.42 (t, $^3J_{H,H}$ = 2.5 Hz, 2H, 2/5 Cp*H*), 3.61 (m, 6H, $C_4H_8O$), 1.77 (m, 6H, $C_4H_8O$), 0.29 (s, 9H, Si*Me$_3$*) ppm. $^{13}$C{$^1$H} NMR (101 MHz, [D$_8$]thf, 26 °C): δ = 126.4 (*Cp*), 122.0 (*Cp*), 120.3 (*Cp*), 68.2 ($C_4H_8O$), 26.4 ($C_4H_8O$), 1.2 (Si*Me$_3$*) ppm. IR (DRIFT): ṽ = 3071 (vw), 2950 (w), 2925 (w), 2889 (w), 1683 (vw), 1651 (vw), 1594 (vw), 1456 (w), 1442 (w), 1404 (vw), 1364 (w), 1310 (vw), 1245 (m), 1173 (m), 1040 (m), 1012 (m), 901 (m), 835 (vs), 793 (vs), 759 (m), 752 (m), 704 (w), 693 (w), 638 (w), 627 (w), 419 (vs) $cm^{-1}$.

**[Cp*$_6$La$_6$I$_8$(AlMe$_4$)$_4$] (2).** A solution of SiMe$_3$I (116 mg, 0.58 mmol) in *n*-hexane (2 mL) was added to a solution of [Cp*La(AlMe$_4$)$_2$] (200 mg, 0.45 mmol) in *n*-hexane (2 mL). The reaction was stirred overnight at ambient temperatures. The precipitate was allowed to settle, washed with *n*-hexane (3 x 1 mL), and dried *in vacuo* to afford **2** as white powder (200 mg, 0.065 mmol, 87%). Crystals could be obtained by performing the reaction under the same conditions without stirring. $^1$H NMR (500 MHz, [D$_6$]benzene, 26 °C): δ = 2.33 (broad irregular signal, 75 H, Cp*), -0.09 (s, 12H, *Me*Al) ppm; due to the poor solubility of **2** any meaningful NMR spectra could not be obtained. IR (DRIFT): ṽ = 2954 (s), 2915 (s), 2857 (m), 1487 (w), 1454 (m), 1436 (m), 1419 (w), 1388 (vw), 1378 (w), 1195 (m), 1037 (m), 1026 (m), 768 (w), 698 (vs), 616 (s), 583 (m), 555 (w), 523 (w) $cm^{-1}$. Elemental analysis of **2** calculated for $C_{76}H_{138}Al_4I_8La_6$ x $C_6H_{14}$ (3094.71 g/mol): C 31.83, H 4.95; found: C 32.26, H 4.76.

**[Cp*LaI$_2$]$_9$ (3).** [Cp*La(AlMe$_4$)$_2$] (**1a**, 322 mg, 0.718 mmol) was dissolved in 5 ml toluene, and $Me_3SiI$ (287 mg, 1.436 mmol) was added under pivoting and afterwards the solution was heated to the boiling point for 15 min. Upon storing at ambient temperature, crystallization occurred. After 8 h, 181 mg of **3** could be harvested as colorless crystals by decanting the mother liquor and drying in oil pump vacuum. After 36 h, another 54 mg of **3** were harvested, adding up to an overall yield of 58%. IR (DRIFT): 2972 (s), 2940 (s), 2898 (vs), 2855 (s), 1494 (m), 1451 (s), 1432 (s), 1377 (s), 1023 (s), 728 (s), 694 (m), 594 (w), 464 (w) $cm^{-1}$. Elemental analysis of **3** calculated for $C_{90}H_{135}I_{18}La_9\cdot(C_7H_8)_3$ (5027.874 g/mol): C 26.52,

H 3.19; found C 26.37, H 2.87. The cluster is insufficiently soluble for proper NMR spectroscopy in [D$_6$]benzene and dissolves in [D$_8$]thf under formation of [Cp*LaI$_2$(thf)$_3$].

**[Cp'$_6$La$_6$I$_8$(AlMe$_4$)$_4$] (4a).** [Cp'La(AlMe$_4$)$_2$] (**1b**, 56.3 mg, 0.125 mmol) was dissolved in *n*-hexane (3 mL), Me$_3$SiI (50.0 mg, 0.25 mmol) was added, and the vial was shaken vigorously. Within several days, **4a** formed as colorless crystals (23.1 mg, 0.008 mmol, 38%). $^1$H NMR (400 MHz, [D$_6$]benzene, 26 °C): δ = 6.78 (t, $^3J_{H,H}$ = 2.5 Hz, 1H, 3/4 Cp*H*), 6.74 (m, 2H, Cp*H*), 6.66 (t, $^3J_{H,H}$ = 2.5 Hz, 1H, 2/5 Cp*H*), 6.63 (m, 2H, Cp*H*), 0.41 (s, 10H, Si*Me$_3$*), 0.37 (s, 5H, Si*Me$_3$*), -0.04 (s, 3H, Al*Me$_4$*) ppm. $^{13}$C{$^1$H} NMR (101 MHz, [D$_6$]benzene, 26 °C): δ = 125.3 (*Cp*), 123.5 (*Cp*), 122.6 (*Cp*) 122.3 (*Cp*), 2.2 (Al*Me$_4$*), 1.0 (Si*Me$_3$*) ppm. IR (DRIFT): ṽ = 3077 (vw), 2952 (w), 2924 (w), 2913 (w), 2847 (vw), 1443 (w), 1403 (vw), 1365 (w), 1310 (vw), 1248 (s), 1201 (w), 1189 (w), 1171 (m), 1042 (s), 1028 (m), 901 (m), 837 (vs), 799 (vs), 756 (m), 699 (s), 637 (w), 626 (m), 590 (w), 586 (w), 553 (vw), 530 (vw), 420 (w), 416 (w) cm$^{-1}$. Elemental analysis of **4a** calculated for $C_{64}H_{126}I_8La_8Si_6Al_4$ (3020.76 g/mol): C 25.45%, H 4.20%; found: C 27.43%, H 4.47%. The significantly increased carbon content can be attributed to the presence of unreacted **1b**.

**[Cp'$_6$La$_6$Br$_8$(AlMe$_4$)$_4$] (4b).** [Cp'La(AlMe$_4$)$_2$] (**1b**, 45.04 mg, 0.1 mmol) was dissolved in *n*-hexane (3 mL), Me$_3$SiBr (19.9 mg, 0.13 mmol) was added, and the reaction mixture was shaken vigorously. Within several days, **3b** formed as colorless crystals (43.5 mg, 0.016 mmol, 96%). $^1$H NMR (400 MHz, [D$_6$]benzene, 26 °C): δ = 6.49 (t, $^3J_{H,H}$ = 2.5 Hz, 2H, 3/4 Cp*H*), 6.19 (t, $^3J_{H,H}$ = 2.5 Hz, 2H, 2/5 Cp*H*), 0.61 (s, 6H, Si*Me$_3$*), 0.57 (s, 9H, Si*Me$_3$*), 0.43 (s, 3H, Si*Me$_3$*), 0.17 (s, 9H, Si*Me$_3$*), -0.22 (bs, 6H, Al*Me$_4$*), -0.24 (bs, 3H, Al*Me$_4$*) ppm. $^{13}$C{$^1$H} NMR (101 MHz, [D$_6$]benzene, 26 °C): δ = 123.2 (*Cp*), 119.7 (*Cp*), 2.2 (Al*Me$_4$*), 0.9 (Si*Me$_3$*), 0.2 (Si*Me$_3$*) ppm. IR (DRIFT): ṽ = 3071 (vw), 2951 (w), 2914 (w), 2894 (w), 2846 (vw), 1443 (vw), 1404 (vw), 1365 (vw), 1309 (vw), 1248 (m), 1188 (w), 1173 (m), 1042 (m), 902 (m), 839 (vs), 798 (vs), 756 (m), 701 (s), 638 (w), 626 (m), 591 (w), 550 (w), 528 (vw), 421 (m) cm$^{-1}$. Elemental analysis of **4b** calculated for $C_{70}H_{140}Br_8La_6Si_6$ (2730.99 g/mol): C 30.79%, H 5.17%; found: C 30.87%, H 5.06%.

**[Cp'LaI$_2$]$_{12}$ (5a).** [Cp'La(AlMe$_4$)$_2$] (**1b**, 56.3 mg, 0.13 mmol) was dissolved in *n*-hexane (15 mL), and Me$_3$SiI (62.5 mg, 0.31 mmol) was added under vigorous stirring. After stirring the reaction mixture for 4 h at ambient temperature, the suspension was filtered. Within several hours at ambient temperature, **5** formed as colorless crystals (31.70 mg, 0.01 mmol, 48%). $^1$H NMR (400 MHz, [D$_6$]benzene, 26 °C): δ = 7.20 (t, $^3J_{H,H}$ = 2.5 Hz, 2H, 3/4 Cp*H*), 7.00 (t, $^3J_{H,H}$ = 2.5 Hz, 2H, 2/5 Cp*H*), 0.61 (s, 9H, Si*Me$_3$*) ppm. $^{13}$C{$^1$H} NMR (101 MHz, [D$_6$]benzene, 26 °C): δ = 131.7 (*Cp*), 125.5 (*Cp*), 123.8 (*Cp*), 1.8 (Si*Me$_3$*) ppm. IR (DRIFT): ṽ = 3079 (vw), 2950 (w), 2893 (vw), 2720 (vw), 1937 (vw), 1742 (vw), 1687 (vw), 1649

(vw), 1590 (vw), 1441 (w), 1405 (w), 1365 (w), 1309 (vw), 1248 (vs), 1170 (m), 1042 (s), 901 (s), 845 (vs), 837 (vs), 830 (vs), 802 (vs), 795 (vs), 755 (m), 692 (vw), 639 (w), 626 (m), 426 (vs), 420 (s) cm$^{-1}$. Elemental analysis of **5** calculated for $C_{96}H_{156}I_{24}La_{12}Si_{12}$ (6359.80 g/mol): C 18.13%, H 2.47%; found: C 18.26%, H 2.76%.

**[Cp'LaBr$_2$]$_{12}$ (5b).** [Cp'La(AlMe$_4$)$_2$] (**1b**, 56.3 mg, 0.13 mmol) was dissolved in *n*-hexane (15 mL), and $Me_3GeBr$ (61.8 mg, 0.31 mmol) was added under vigorous stirring. After stirring the reaction mixture for 4 h at ambient temperature, the suspension was filtered. Within several days at ambient temperature, **5b** and **5b'** (*n*-hexane in crystal lattice) formed as colorless crystals (25.35 mg, 0.01 mmol, 47%). $^1$H NMR (400 MHz, [D$_6$]benzene, 26 °C): δ = 7.08 (t, $^3J_{H,H}$ = 2.5 Hz, 2H, 3/4 Cp*H*), 6.97 (t, $^3J_{H,H}$ = 2.5 Hz, 2H, 2/5 Cp*H*), 0.63 (s, 9H, Si*Me$_3$*) ppm. $^{13}$C{$^1$H} NMR (101 MHz, [D$_6$]benzene, 26 °C): δ = 131.0 (*Cp*), 125.2 (*Cp*), 123.7 (*Cp*), 1.2 (Si*Me$_3$*) ppm. IR (DRIFT): ṽ = 3092 (vw), 3079 (vw), 2951 (w), 2893 (w), 1743 (vw), 1689 (vw), 1650 (vw), 1593 (vw), 1443 (w), 1403 (w), 1365 (w), 1311 (w), 1246 (m), 1214 (vw), 1192 (vw), 1172 (m), 1061 (w), 1042 (m), 902 (w), 853 (m), 836 (m), 801 (m), 756 (w), 692 (w), 640 (w), 626 (w), 422 (m), 415 (vs) cm$^{-1}$. Elemental analysis of **5b** calculated for $C_{96}H_{156}Br_{24}La_{12}Si_{12}$ (5232.04 g/mol): C 22.04%, H 3.01%; found: C 22.92%, H 2.96%. The high carbon value can be attributed to residual *n*-hexane in the crystal lattice.

**[(*μ*-Cp)$_2$Cp'$_8$La$_8$I$_{14}$] (6).** In a 20-mL vial, [Cp'La(AlMe$_4$)$_2$] (**1b**, 56.43 mg, 0.13mmol) was dissolved in *n*-hexane (10 mL). In a 5-mL vial, $Me_3SiI$ (62.53 mg, 0.31 mmol) was dissolved in *n*-hexane (1 mL). The smaller vial was placed inside the bigger one with the [Cp'La(AlMe$_4$)$_2$] solution, and the large vial was sealed afterwards. Diffusion of the $Me_3SiI$ into the [Cp'La(AlMe$_4$)$_2$] solution and storing the vial at -40 °C for 1 h led to the formation of crystalline **6**. $^1$H NMR (400 MHz, [D$_6$]benzene, 26 °C): δ = 7.19 (t, $^3J_{H,H}$ = 2.5 Hz, 3/4 Cp*H*), 7.00 (t, $^3J_{H,H}$ = 2.5 Hz, 2/5 Cp*H*), 6.92 (t, $^3J_{H,H}$ = 2.5 Hz, *μ*-Cp*H*), 6.88 (t, $^3J_{H,H}$ = 2.5 Hz, *μ*-Cp*H*), 6.68 (t, $^3J_{H,H}$ = 2.5 Hz, *μ*-Cp*H*), 6.62 (t, $^3J_{H,H}$ = 2.5 Hz, *μ*-Cp*H*), 0.61 (s, Si*Me$_3$*) ppm. $^{13}$C{$^1$H} NMR (101 MHz, [D$_6$]benzene, 26 °C): δ = 120.4 (*Cp*), 116.9 (*μ-Cp*), 1.8 (Si*Me$_3$*) ppm. Any meaningful IR spectra and elemental analysis could not be performed due to the presence of co-products.

**[Cp'$_5$La$_5$Br$_9$(AlBr$_2$Me$_2$)]$_2$ (7).** In a 20-mL vial, [Cp'La(AlMe$_4$)$_2$] (56.43 mg, 0.13mmol) was dissolved in *n*-hexane (10 mL). In a 5-mL vial, $Me_3GeBr$ (61.75 mg, 0.31 mmol) was dissolved in *n*-hexane (1 mL). The smaller vial was placed inside the larger one with the [Cp'La(AlMe$_4$)$_2$] solution, and the large vial was sealed afterwards. Storing the vial at ambient temperature led to the formation of crystalline **7** after several days. $^1$H NMR (400 MHz, [D$_6$]benzene, 26 °C): δ = 7.20 (t, $^3J_{H,H}$ = 2.5 Hz, 2H, Cp*H*), 7.07 (t,

$^3J_{H,H}$ = 2.5 Hz, 5H, CpH), 6.94 (t, $^3J_{H,H}$ = 2.5 Hz, 3H, Cp*H*), 6.87 (t, $^3J_{H,H}$ = 2.5 Hz, 2H, Cp*H*), 6.84 (t, $^3J_{H,H}$ = 2.5 Hz, 2H, Cp*H*), 6.72 (t, $^3J_{H,H}$ = 2.5 Hz, 2H, Cp*H*), 6.64 (t, $^3J_{H,H}$ = 2.5 Hz, 2H, Cp*H*), 0.63 (s, 9H, Si*Me₃*), 0.59 (s, 9H, Si*Me₃*), 0.50 (s, 9H, Si*Me₃*), 0.42 (s, 9H, Si*Me₃*), 0.33 (bs, 6H, Al*Me₂*) ppm. $^{13}C\{^1H\}$ NMR (101 MHz, [D$_6$]benzene, 26 °C): δ = 132.0 (*Cp*), 125.9 (*Cp*), 123.9 (*Cp*), 122.8 (*Cp*), 120.4 (*Cp*), 118.9 (*Cp*), 118.3 (*Cp*), 1.1 (Si*Me₃*), 0.8 (Si*Me₃*), 0.5 (Si*Me₃*) ppm. IR spectroscopy and elemental analysis on **7** were not performed due to the presence of co-products.

**[Cp'LaCl$_2$]$_{10}$ (8).** [Cp'La(AlMe$_4$)$_2$] (**1b**, 44.3 mg, 0.1 mmol) was dissolved in *n*-hexane (15 mL) and Me$_3$GeCl (37.7 mg, 0.25 mmol) was added under vigorous stirring. After stirring the reaction mixture for 7 d at ambient temperature, the suspension was filtered, and the solvent was removed *in vacuo* to give **7** as a colorless solid. Crystalline **8** was obtained from a saturated toluene solution at -40 °C (11.58 mg, 0.003 mmol, 33%). $^1H$ NMR (400 MHz, [D$_6$]benzene, 26 °C): δ = 7.07 (t, $^3J_{H,H}$ = 2.5 Hz, 2H, 3/4 Cp*H*), 7.01 (t, $^3J_{H,H}$ = 2.5 Hz, 2H, 2/5 Cp*H*), 0.55 (s, 9H, Si*Me₃*) ppm. $^{13}C\{^1H\}$ NMR (101 MHz, [D$_6$]benzene, 26 °C): 131.8 (*Cp*), 125.7 (*Cp*), 123.6 (*Cp*), 120.4 (*Cp*), 0.9 (Si*Me₃*), 0.5 (Si*Me₃*) ppm. IR (DRIFT): ṽ = 2953 (vw), 2894 (vw), 1444 (vw), 1406 (vw), 1365 (vw), 1312 (vw), 1249 (m), 1192 (vw), 1174 (w), 1044 (m), 1014 (vw), 904 (m), 848 (vs), 802 (s), 797 (s), 757 (w), 697 (w), 638 (vw), 628 (vw), 580 (vw), 422 (m), 417 (w) cm$^{-1}$. Elemental analysis of **8** calculated for $C_{80}H_{130}Cl_{20}La_{10}Si_{10}$ (3470.82 g/mol): C 27.68%, H 3.78%; found: C 27.59%, H 4.19%.

# NMR Spectroscopy

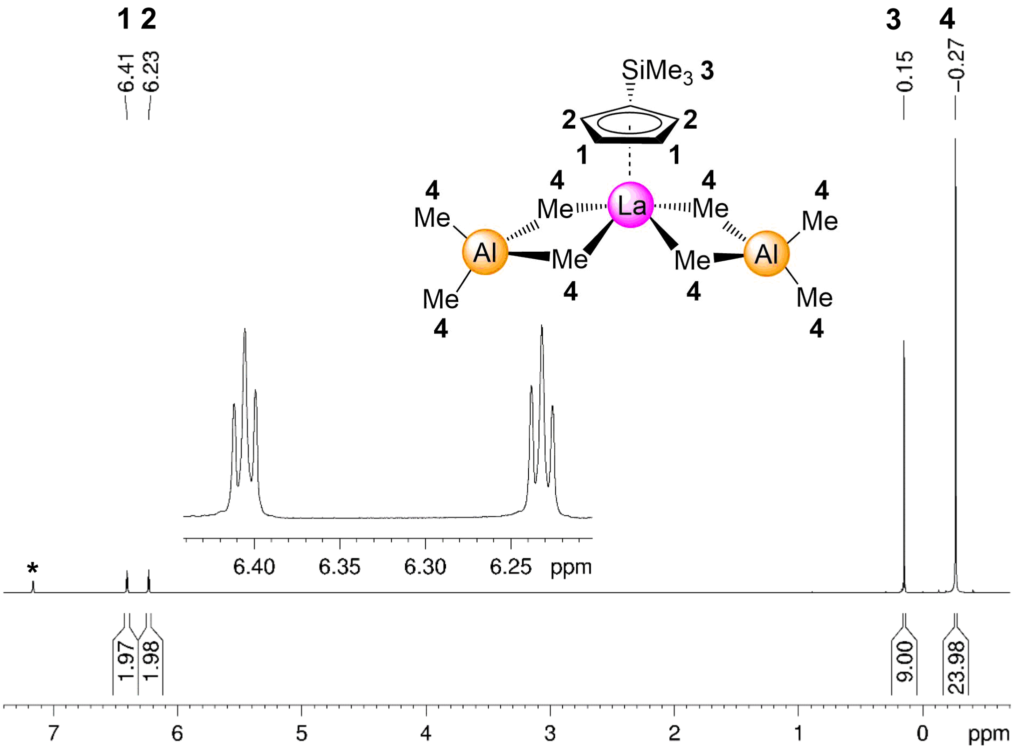


**Figure S1.** $^{1}H$ NMR spectrum (400 MHz) of **1b** in [D$_6$]benzene at 26 °C. The solvent residual signal is marked with an asterisk.

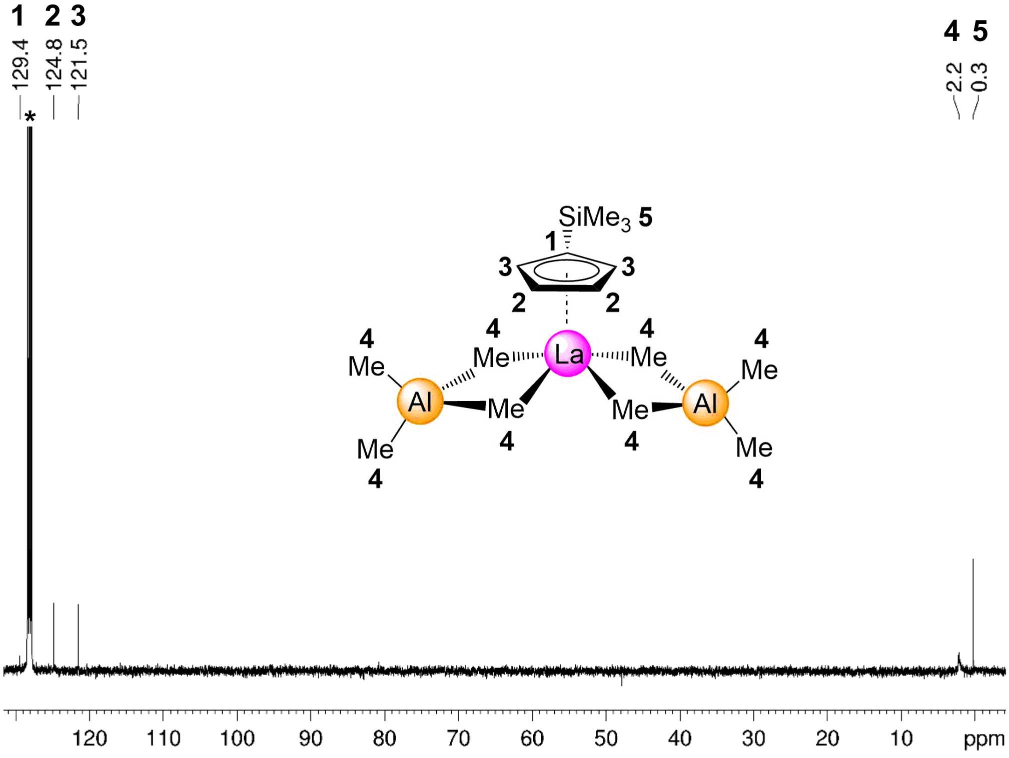


**Figure S2.** $^{13}C\{^{1}H\}$ NMR spectrum (101 MHz) of **1b** in [D$_6$]benzene at 26 °C. The solvent residual signal is marked with an asterisk.

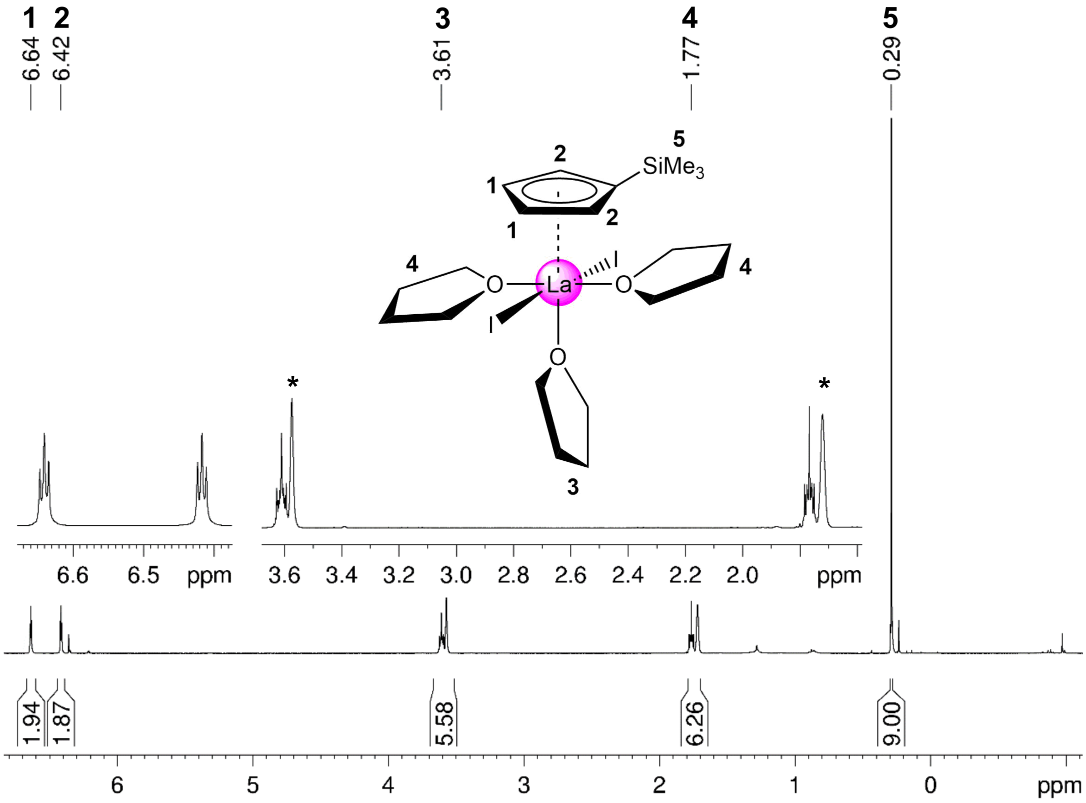


**Figure S3.** $^{1}H$ NMR spectrum (400 MHz) of [Cp'LaI$_2$(thf)$_3$] in [D$_8$]thf at 26 °C. The solvent residual signal is marked with an asterisk.

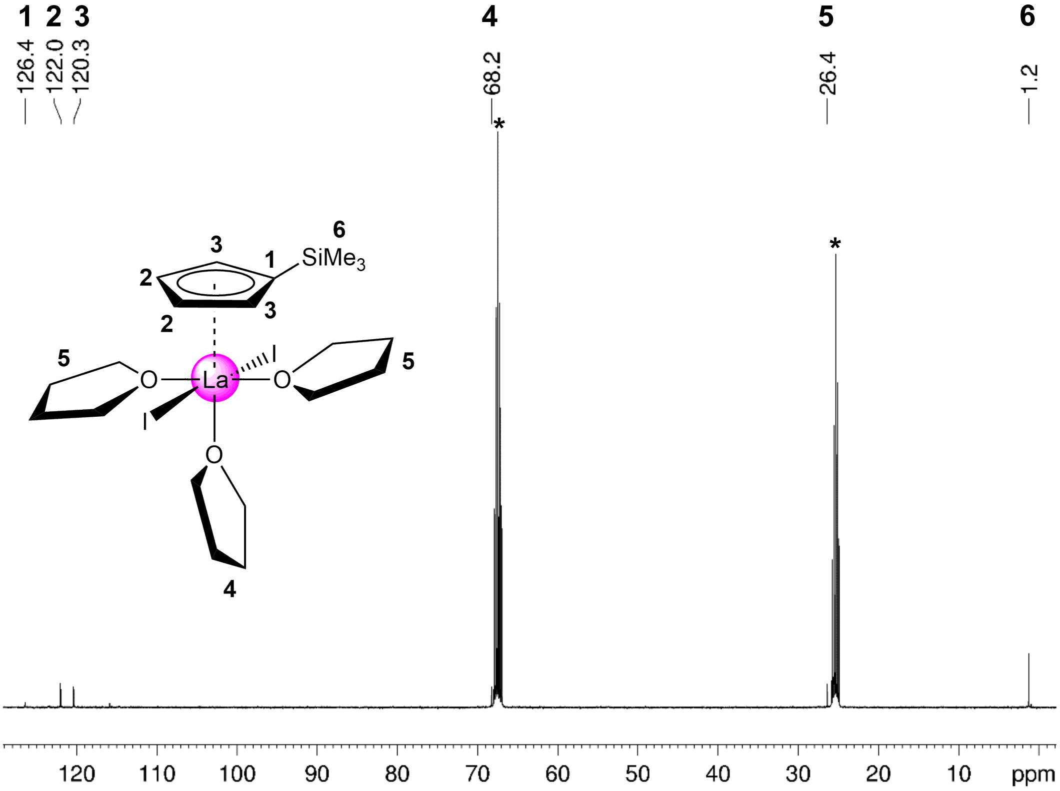


**Figure S4.** $^{13}C\{^{1}H\}$ NMR spectrum (101 MHz) of [Cp'LaI$_2$(thf)$_3$ in [D$_8$]thf at 26 °C. The solvent residual signal is marked with an asterisk.

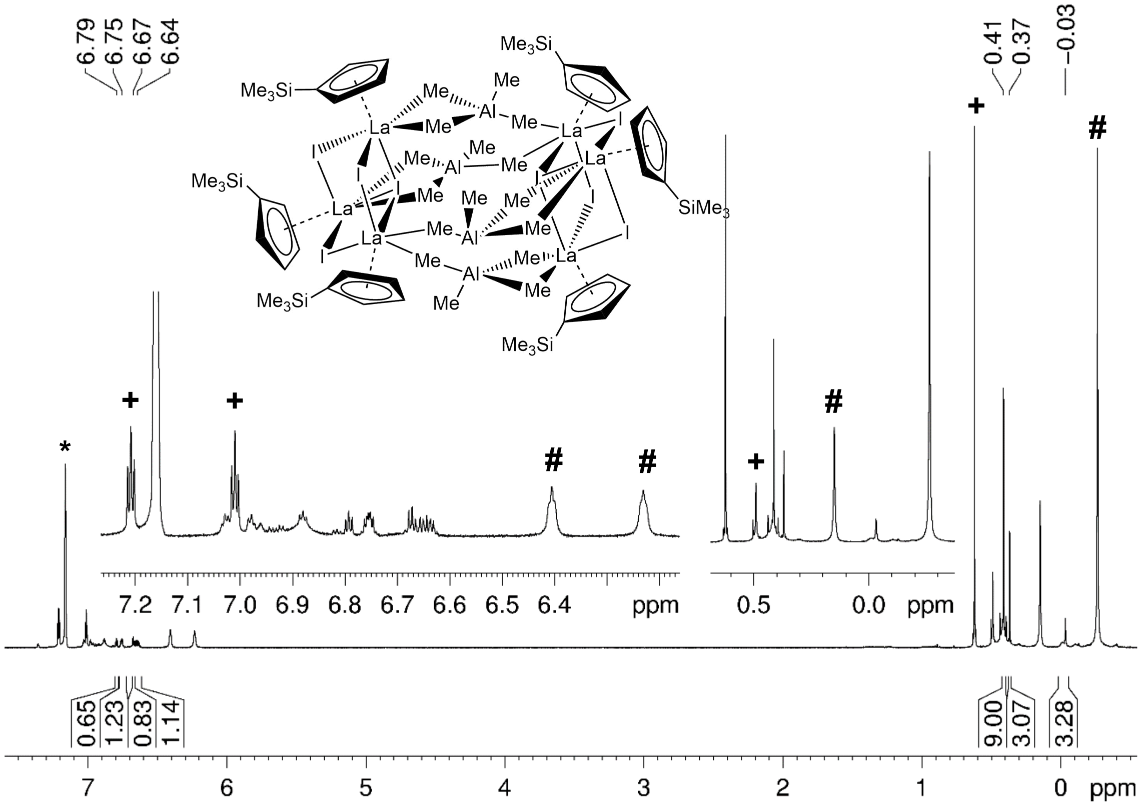

**Figure S5.** $^{1}H$ NMR spectrum (400 MHz) of **4a** in [D$_6$]benzene at 26 °C. The solvent residual signal is marked with an asterisk (+: **5a**; #: **1b**).

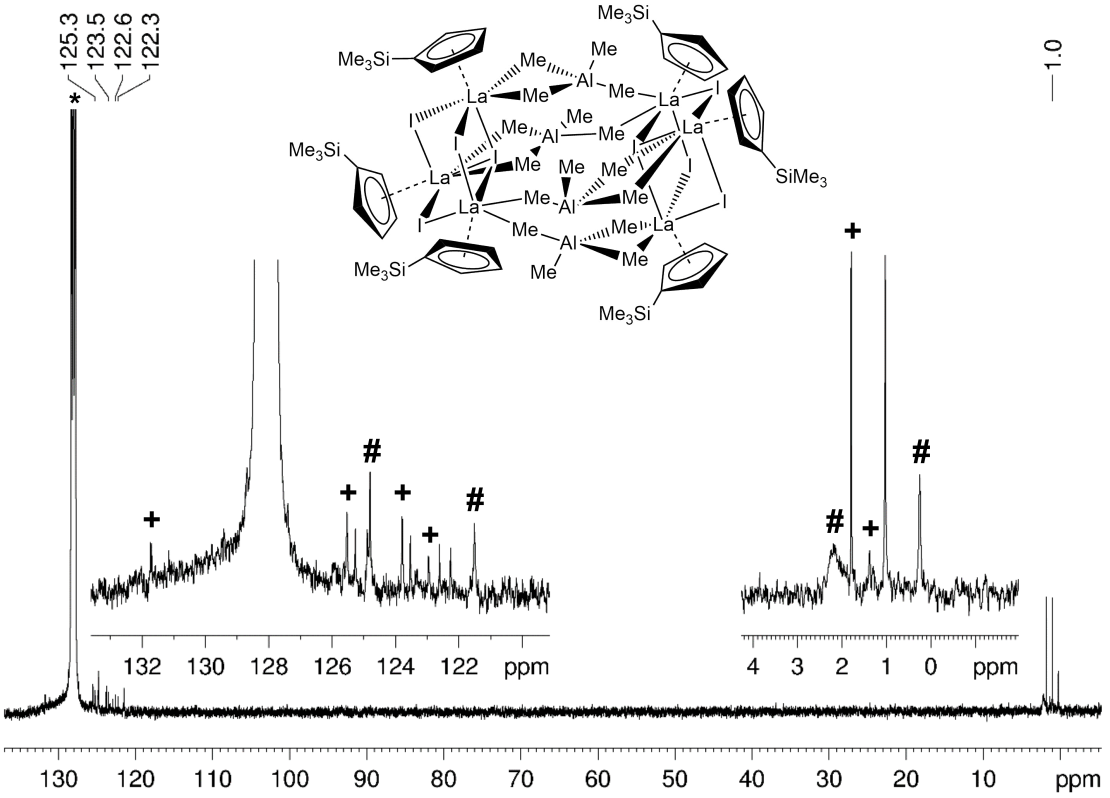

**Figure S6.** $^{13}C\{^{1}H\}$ NMR spectrum (101 MHz) of **4a** in [D$_6$]benzene at 26 °C. The solvent residual signal is marked with an asterisk (+: **5a**; #: **1b**).

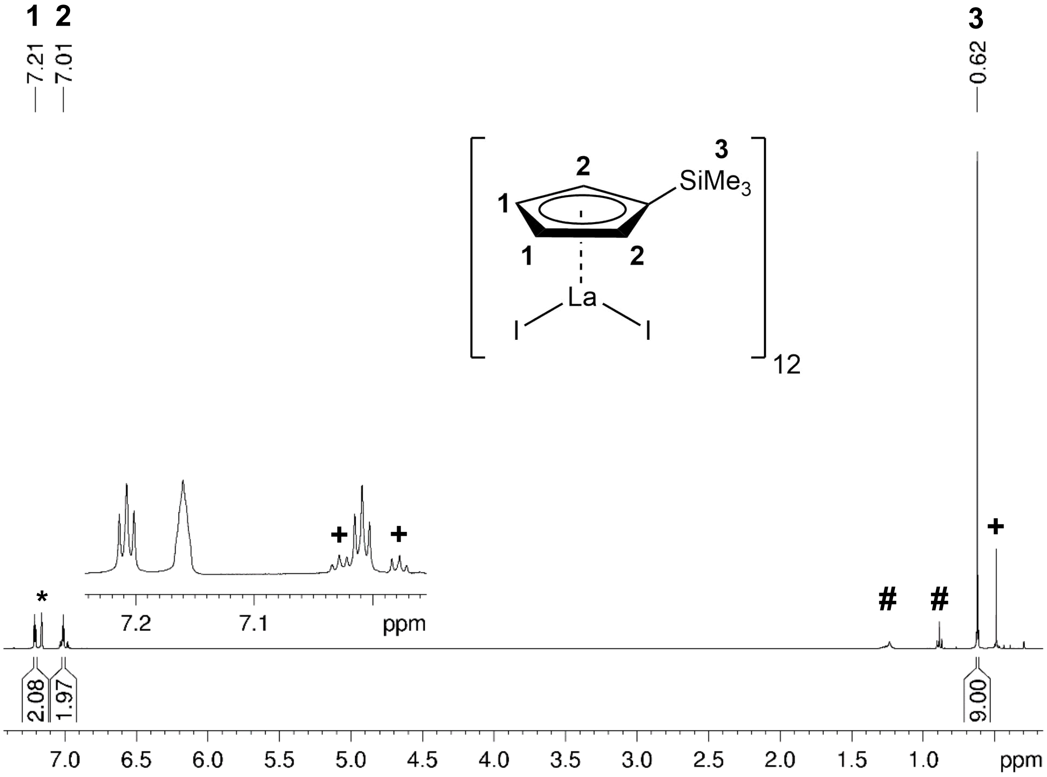


**Figure S7.** $^{1}H$ NMR spectrum (400 MHz) of **5a** in [D6]benzene at 26 °C. The solvent residual signal is marked with an asterisk (+: side product; #: *n*-hexane).

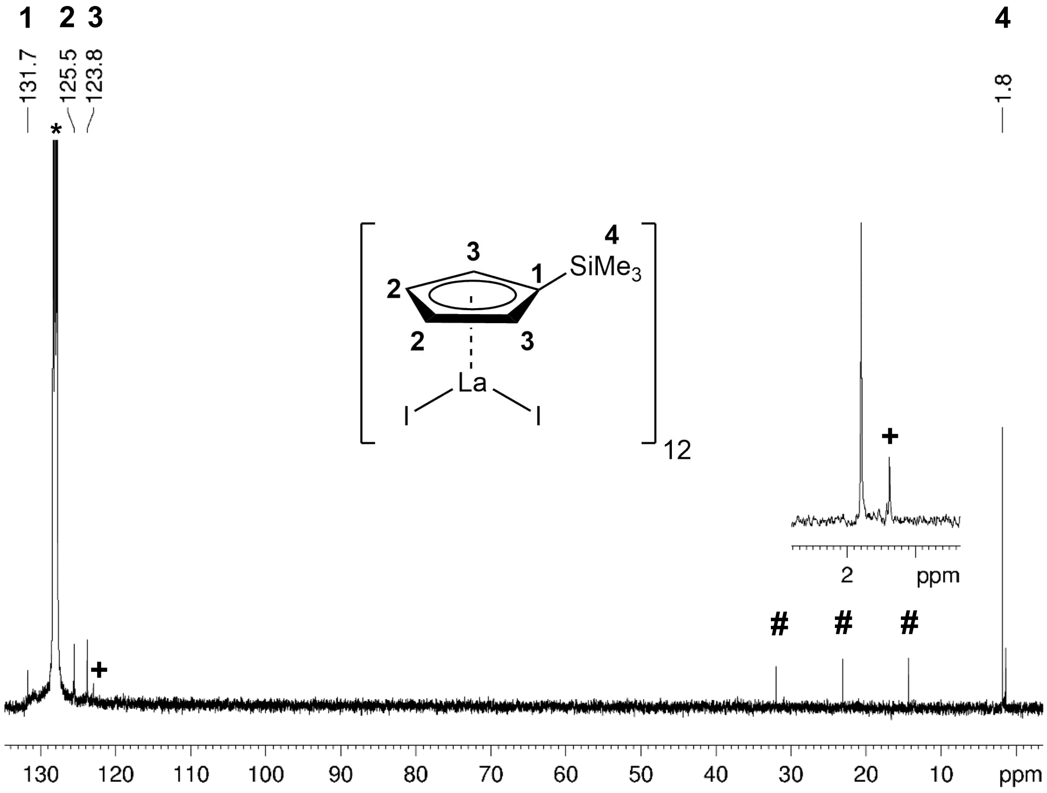


**Figure S8.** $^{13}C\{^{1}H\}$ NMR spectrum (101 MHz) of **5a** in [D6]benzene at 26 °C. The solvent residual signal is marked with an asterisk (+: side product; #: *n*-hexane).

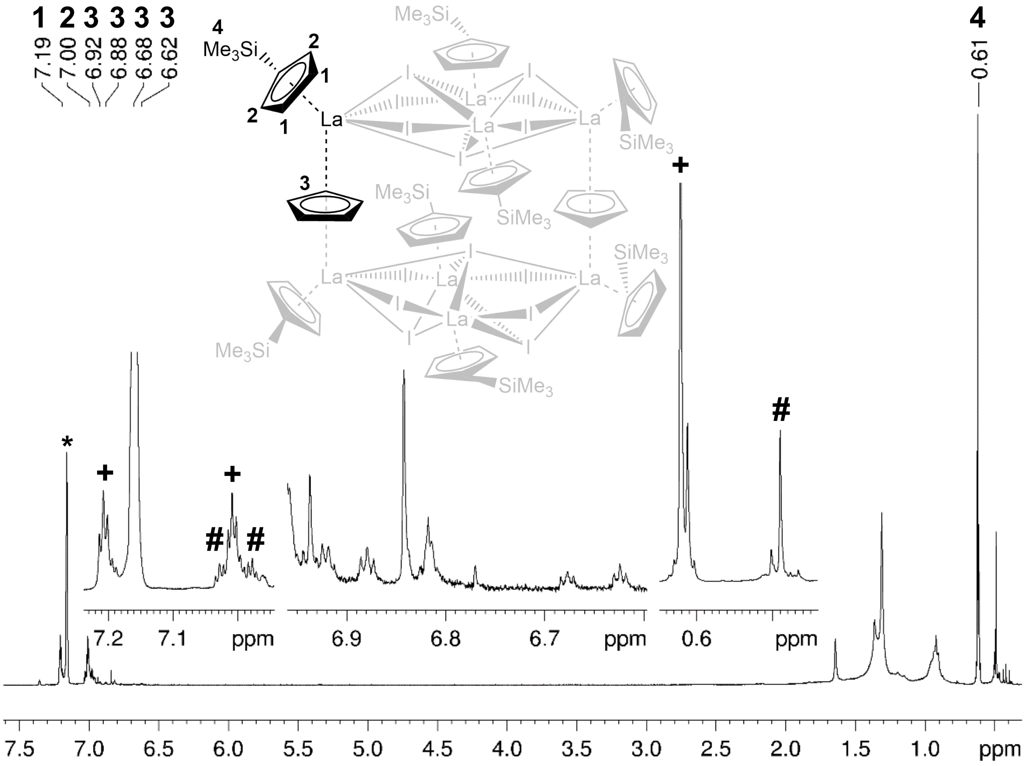


**Figure S9**. $^{1}$H NMR spectrum (400 MHz) of **6** in [D$_6$]benzene at 26 °C. The solvent residual signal is marked with an asterisk (+: **5a**; #: side product).

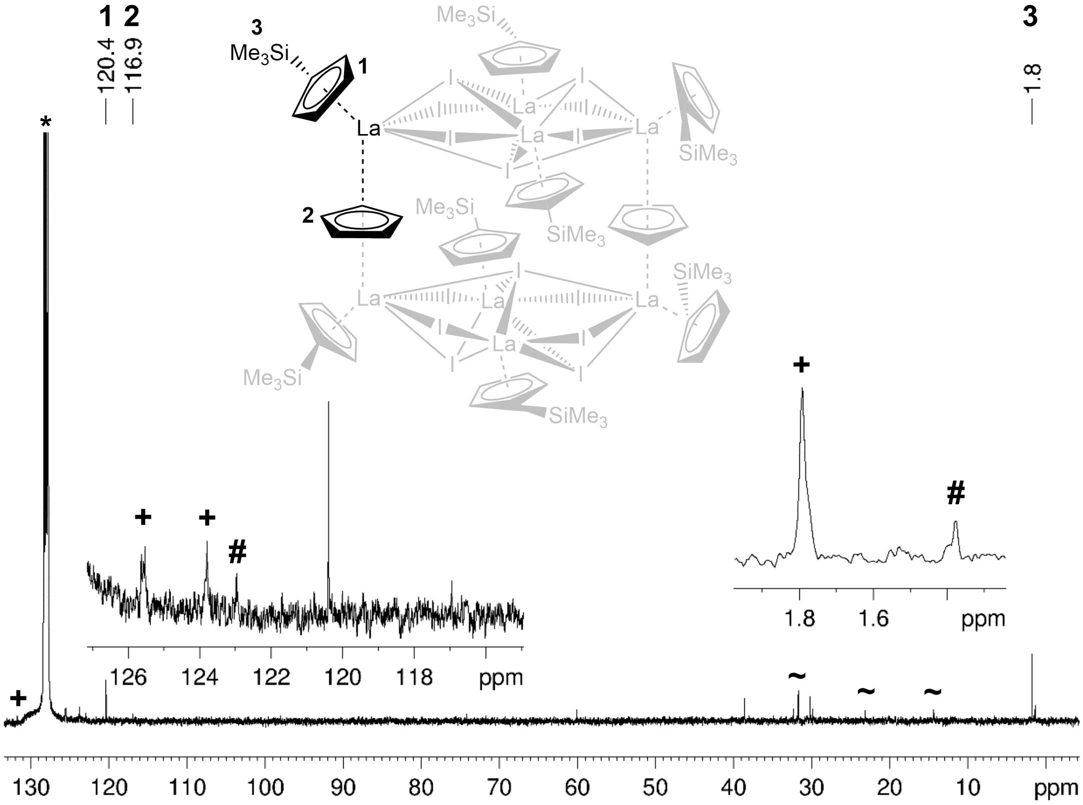


**Figure S10.** $^{13}$C{$^{1}$H} NMR spectrum (101 MHz) of **6** in [D$_6$]benzene at 26 °C. The solvent residual signal is marked with an asterisk (+: **5a**; #: side product; ~: *n*-hexane).

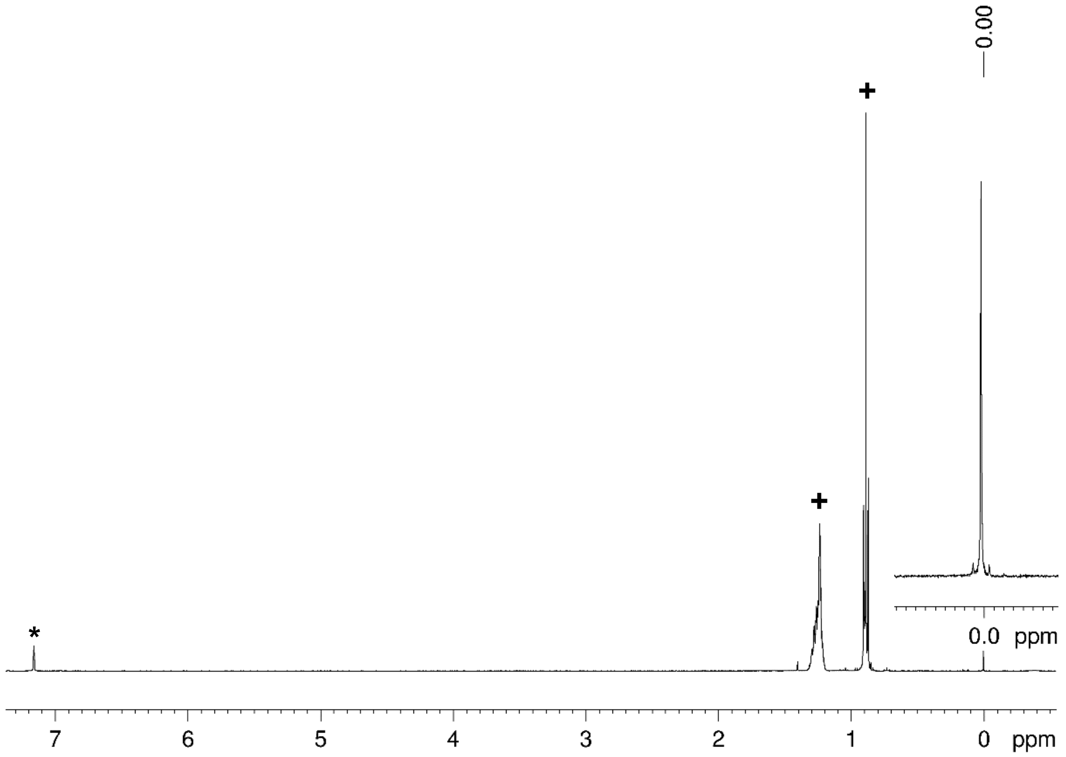


**Figure S11.** $^1$H NMR spectrum (400 MHz) of the residual solution of **6** in [D$_6$]benzene at 26 °C, showing the presence of $Me_6Si_2$ (0.00 ppm) in solution. The solvent residual signal is marked with an asterisk (+: *n*-hexane).

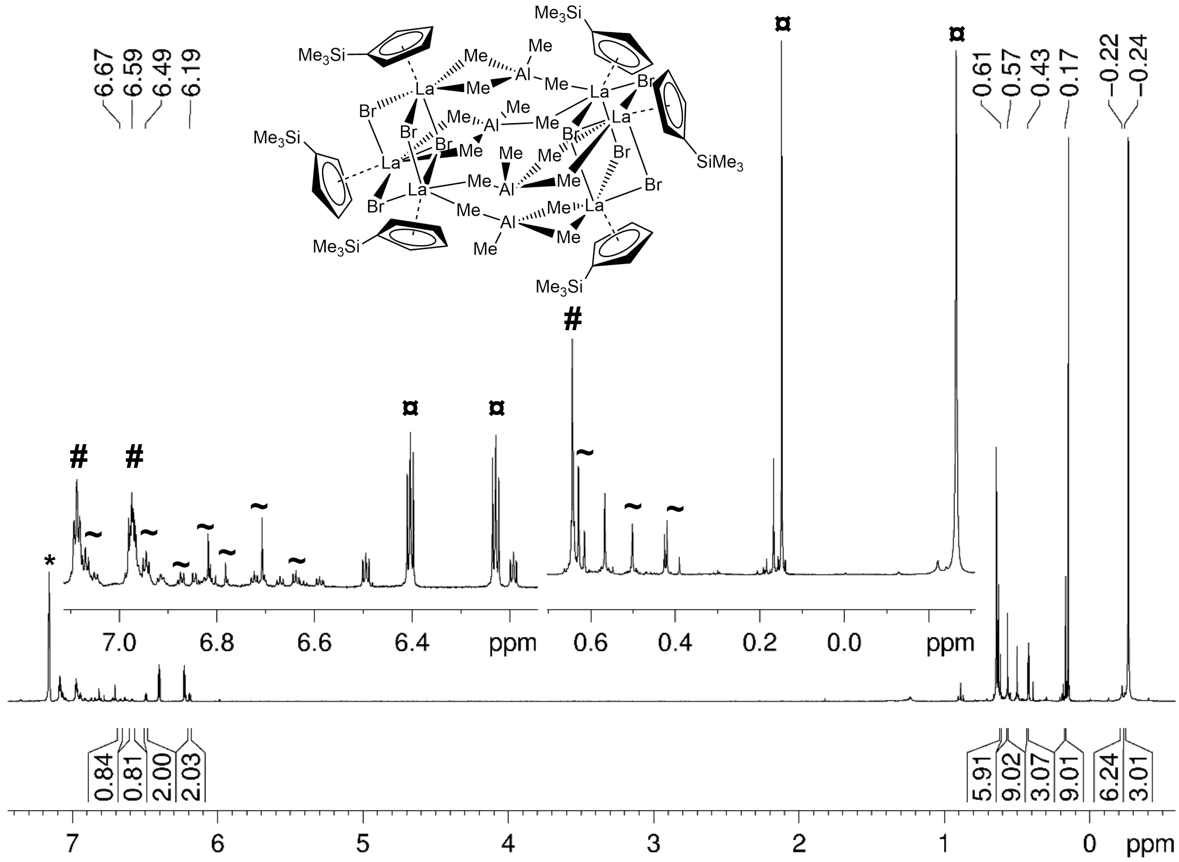


**Figure S12.** $^{1}H$ NMR spectrum (400 MHz) of **4b** in [D$_6$]benzene at 26 °C. The solvent residual signal is marked with an asterisk (+: *n*-hexane, #: **5b**, ~: **7**, ¤: **1b**).

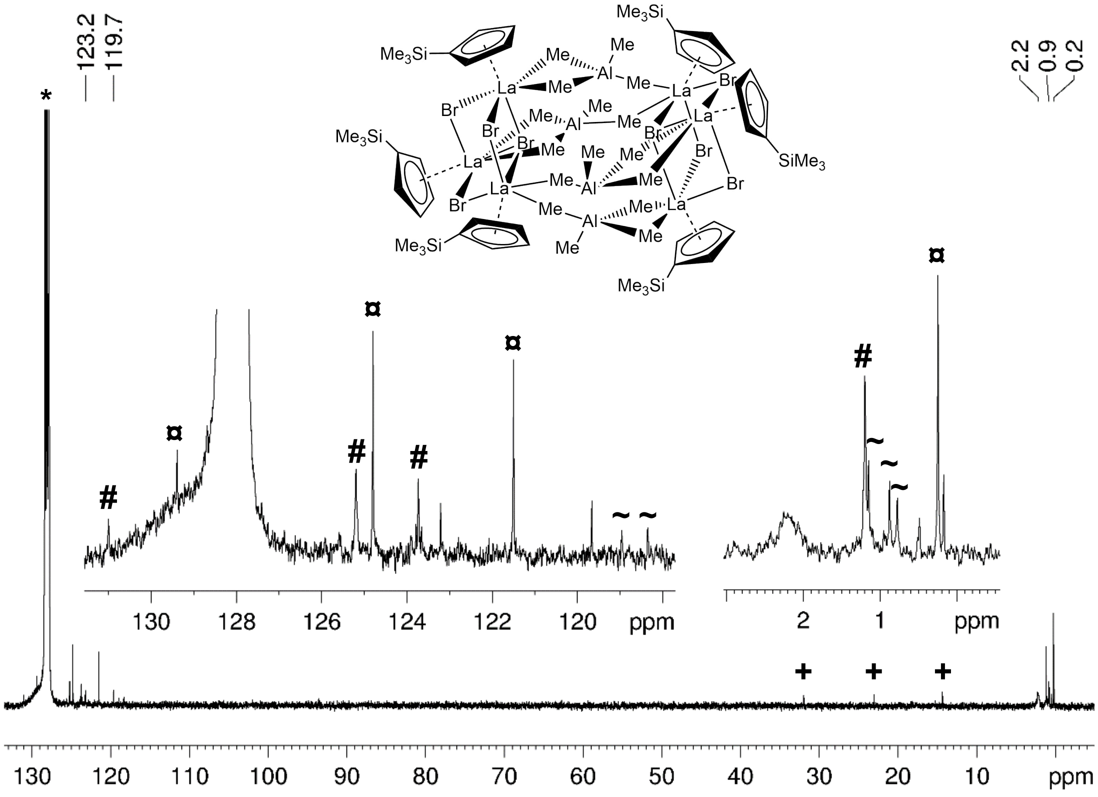


**Figure S13.** $^{13}C\{^{1}H\}$ NMR spectrum (101 MHz) of **4b** in [D$_6$]benzene at 26 °C. The solvent residual signal is marked with an asterisk (+: *n*-hexane, #: **5b**, ~: **7**, ¤: **1b**).

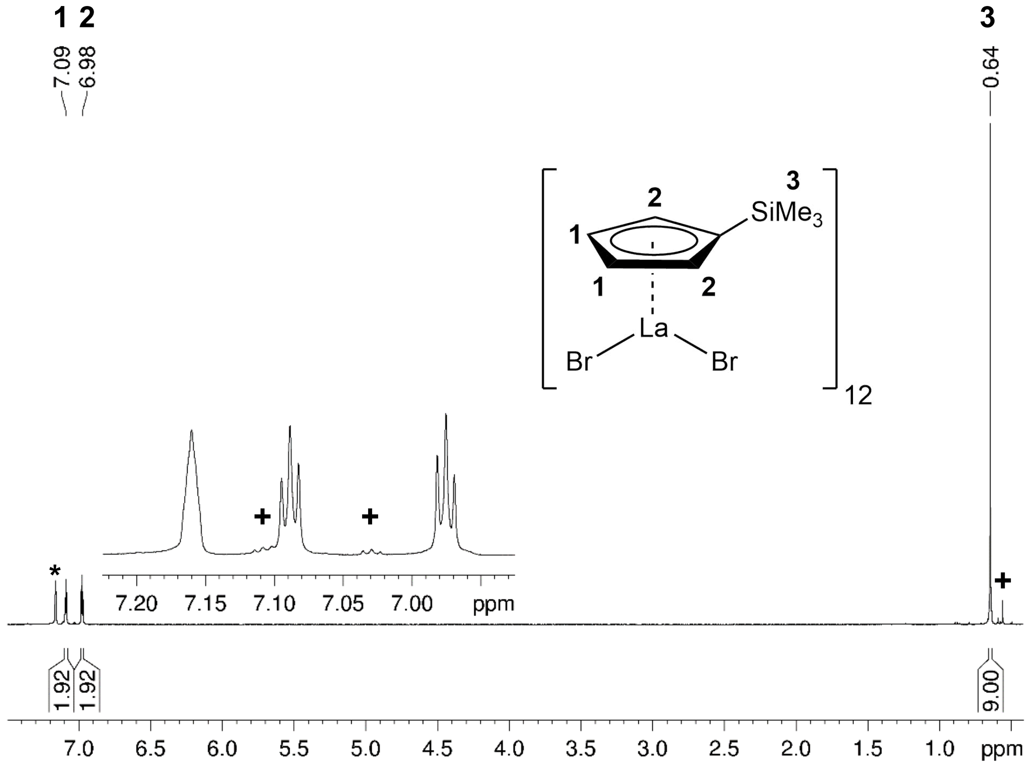


**Figure S14.** $^{1}$H NMR spectrum (400 MHz) of **5b** in [D$_6$]benzene at 26 °C. The solvent residual signal is marked with an asterisk (+: side product).

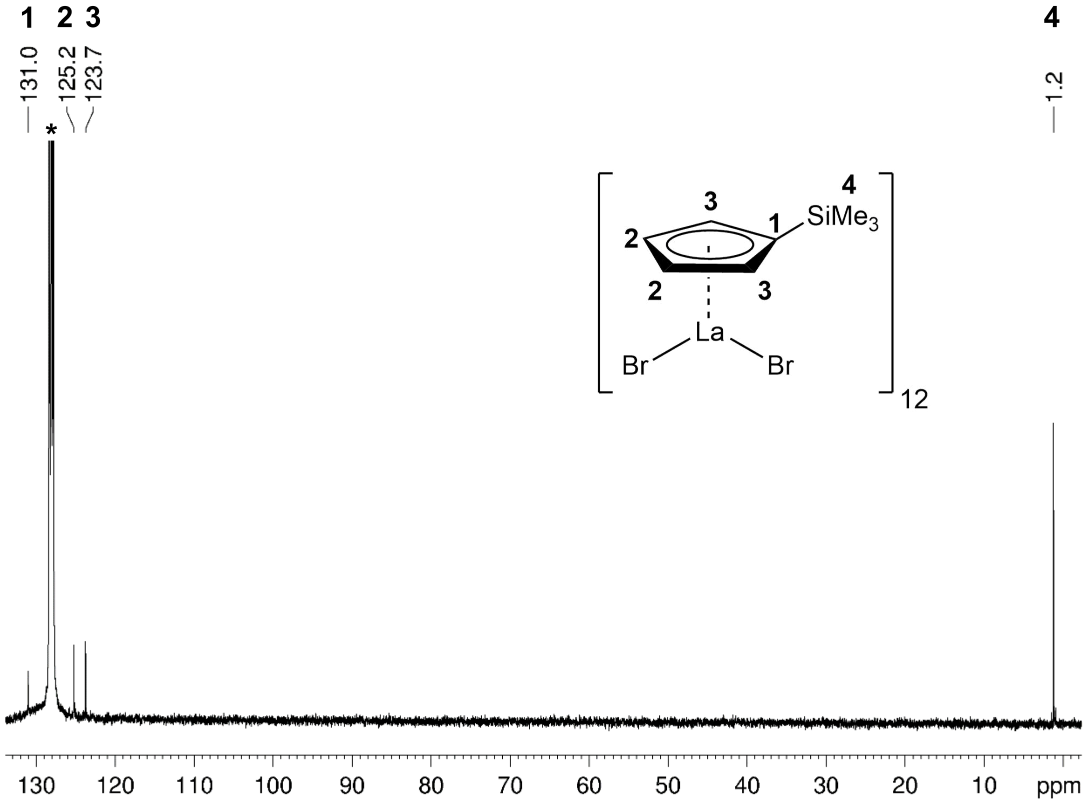


**Figure S15.** $^{13}$C{$^{1}$H} NMR spectrum (101 MHz) of **5b** in [D$_6$]benzene at 26 °C. The solvent residual signal is marked with an asterisk.

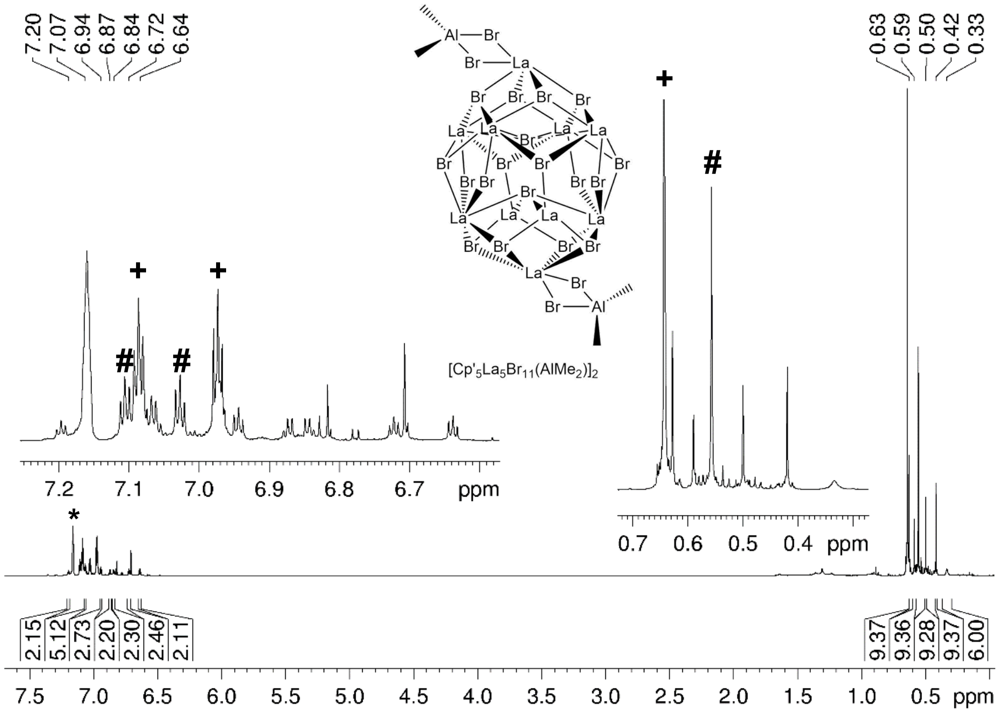


**Figure S16.** $^{1}H$ NMR spectrum (400 MHz) of **7** in [D$_6$]benzene at 26 °C. The solvent residual signal is marked with an asterisk (+: **5b**; #: side product).

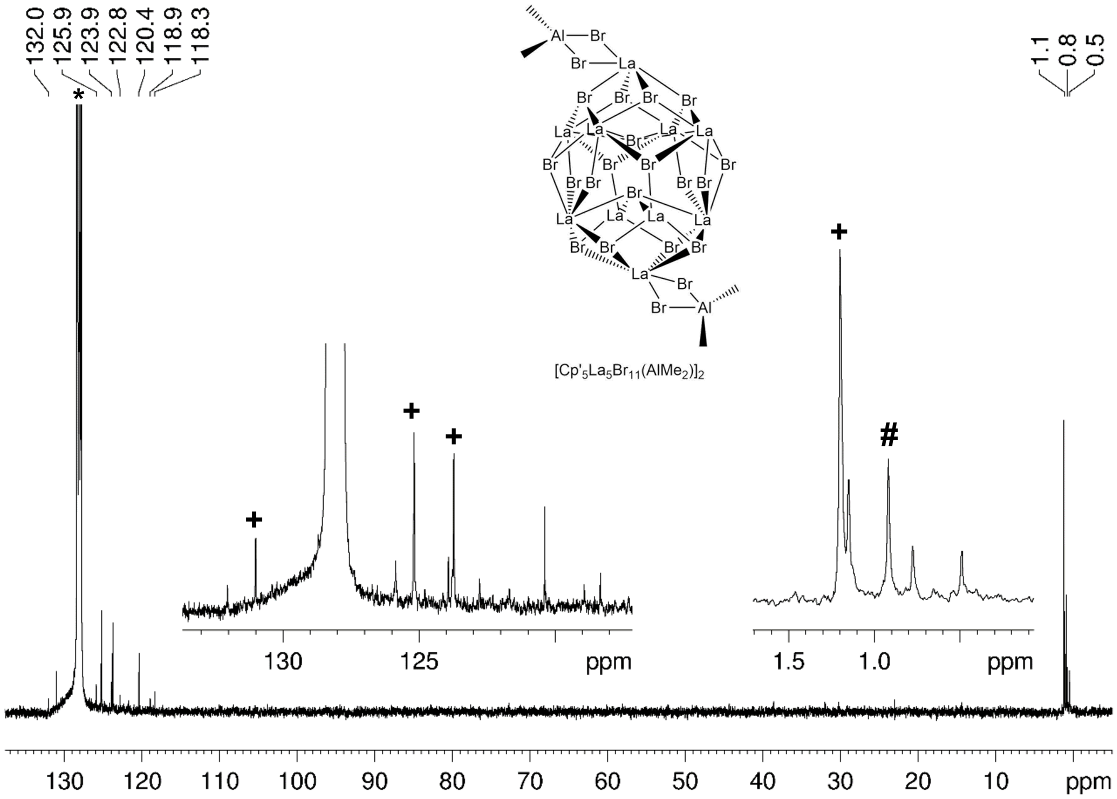


**Figure S17.** $^{13}C\{^{1}H\}$ NMR spectrum (101 MHz) of **7** in [D$_6$]benzene at 26 °C. The solvent residual signal is marked with an asterisk (+: **5b**; #: side product).

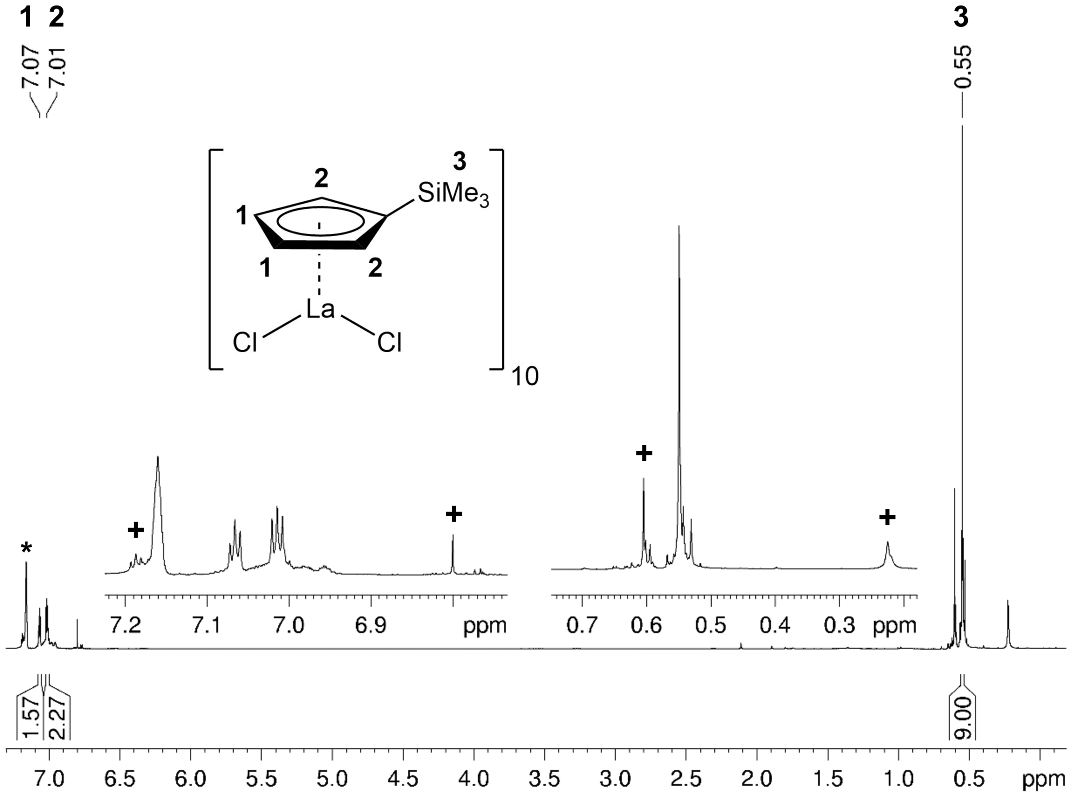


**Figure S18.** $^{1}H$ NMR spectrum (400 MHz) of **8** in [D$_6$]benzene at 26 °C. The solvent residual signal is marked with an asterisk (+: side product).

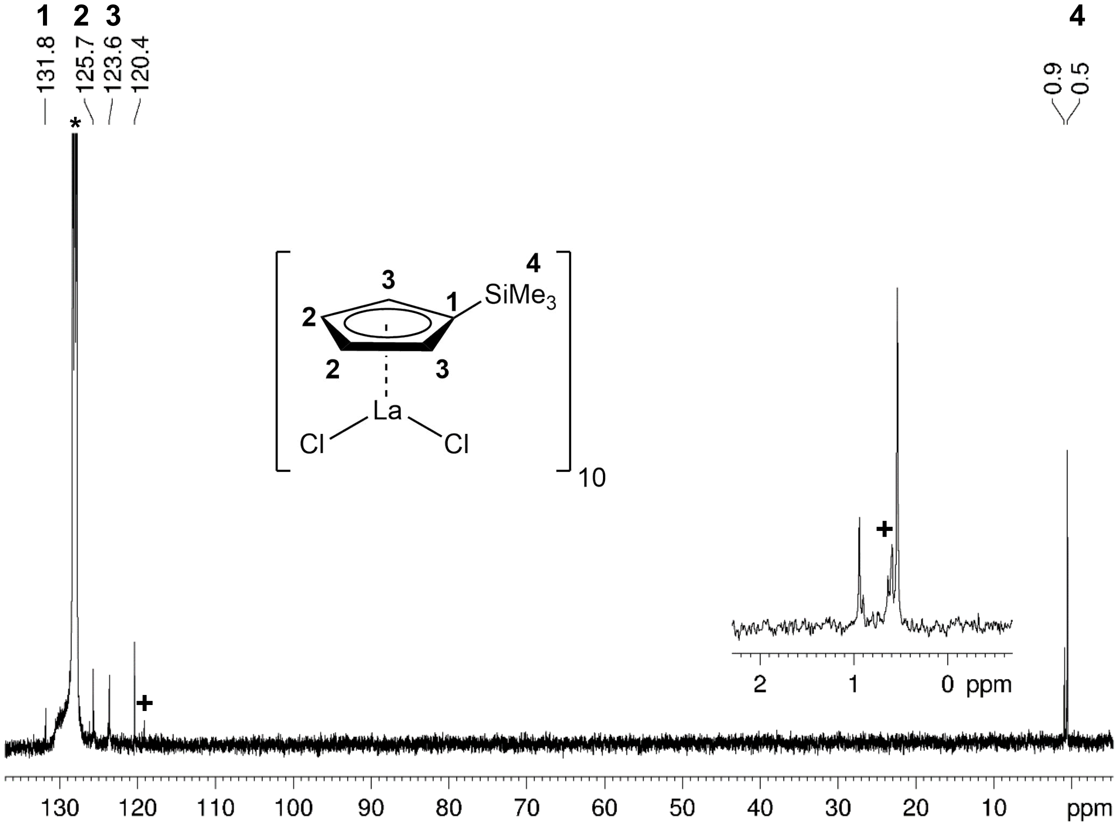


**Figure S19.** $^{13}C\{^{1}H\}$ NMR spectrum (101 MHz) of **8** in [D$_6$]benzene at 26 °C. The solvent residual signal is marked with an asterisk (+: side product).

# Crystallography

**X-Ray Crystallography and Crystal Structure Determinations.** Single-crystals of **1b**, [Cp*La$I_2$(thf)$_3$], [Cp'La$I_2$(thf)$_3$], **4b**, and **8** were grown by standard techniques using saturated solutions of *n*-hexane (**1b**, **4b**), *n*-hexane/THF ([Cp*La$I_2$(thf)$_3$], [Cp'La$I_2$(thf)$_3$]) and toluene (**8**). Due to the insufficient crystal quality of **8**, data could be collected only to a resolution limit of 1.02 Å. Therefore, only a connectivity of **8** can be given. Crystals of **3**, **4a**, **5a**, and **5b** (**5b'**) were grown by aggregation from diluted *n*-hexane (**4a**, **5a**, **5b** (**5b'**)) and toluene (**3**) solutions. Crystals of **6** and **7** were grown by diffusion and subsequent aggregation. Suitable crystals for X-ray structure analyses were selected inside a glovebox, coated with Parabar 10312 (previously known as Paraton N, Hampton Research) and fixed on a nylon/loop glass fiber. All X-ray data except for compound **3** (STOE IPDS II) were collected on a Bruker APEX II DUO instrument equipped with an IμS microfocus sealed tube and QUAZAR optics for MoKα (λ = 0.71073 Å) radiation. The data collection was determined using COSMO[4] employing ω-scans. Raw data were processed using APEX[5] and SAINT,[6] corrections for absorption effects were applied by using SADABS.[7] The structures were solved by direct methods and refined against all data by full-matrix least-squares methods on $F^2$ using SHELXTL[8] and SHELXLE.[9] All graphics were generated employing CCDC Mercury 3.10.1.[10] Further details regarding the refinement and crystallographic data are listed in Table S1 and in the CIF files. CCDC depositions 1992037-1992048 contain all the supplementary crystallographic data for this paper. These data can be obtained free of charge from The Cambridge Crystallographic Data Centre via www.ccdc.cam.ac.uk/data_request/cif.

**Table S1.** Crystallographic data for compounds **1b**, [Cp'LaI$_2$(thf)$_3$], and **2**

| | **1b** | [Cp'LaI$_2$(thf)$_3$] | **2** |
|---|---|---|---|
| CCDC | 1992038 | 1992037 | 1992045 |
| formula | $C_{16}H_{37}Al_2LaSi$ | $C_{20}H_{37}I_2LaSiO_3$ | $C_{88}H_{166}Al_4I_8La_6$ |
| $M_r$ [g/mol] | 450.41 | 746.29 | 3180.78 |
| color/shape | colorless/block | colorless/column | colorless/plate |
| crystal dimensions [mm] | 0.305 x 0.250 x 0.194 | 0.150 x 0.118 x 0.065 | 0.181 x 0.125 x 0.086 |
| crystal system | triclinic | orthorhombic | triclinic |
| space group | $P\bar{1}$ | $P2_12_12_1$ | $P\bar{1}$ |
| a [Å] | 9.7493(7) | 8.219(3) | 15.1879(2) |
| b [Å] | 9.7900(8) | 14.452(5) | 15.4891(2) |
| c [Å] | 27.449(2) | 22.375(8) | 15.6522(2) |
| α [°] | 99.550(2) | 90 | 103.083(3) |
| β [°] | 90.686(2) | 90 | 118.426(2) |
| γ [°] | 119.474(2) | 90 | 104.865(3) |
| V [Å$^3$] | 2235.8(3) | 2657.7 | 2854.7(6) |
| Z | 4 | 4 | 1 |
| T [K] | 101(2) | 173(2) | 100(2) |
| λ [Å] | 0.71073 | 0.71073 | 0.71073 |
| $\rho_{calcd}$ [g/cm$^3$] | 1.338 | 1.865 | 1.850 |
| $\mu$ [mm$^{-1}$] | 2.035 | 3.992 | 4.423 |
| F (000) | 920 | 1432 | 1512 |
| θ range [°] | 2.271 – 29.189 | 2.302 – 30.578 | 1.491 – 30.077 |
| unique reflections | 12091 | 8124 | 16728 |
| observed reflections (I > 2σ) | 11532 | 7672 | 14086 |
| R1/*w*R2 (I > 2σ) | 0.0287/0.0661 | 0.0266/0.0620 | 0.0329/0.0798 |
| R1/*w*R2 (all data) | 0.0305/0.0667 | 0.0290/0.0632 | 0.0417/0.0861 |
| GOF | 1.116 | 0.965 | 1.064 |

**Table S1 (continued).** Crystallographic data for compounds **3**, [Cp*$LaI_2(thf)_3$], and **4a**

| | **3** | [Cp*$LaI_2(thf)_3$] | **4a** |
|---|---|---|---|
| CCDC | 1992039 | 1992043 | 1992044 |
| formula | $C_{90}H_{135}I_{18}La_9 \cdot 2.5\ C_6H_6 \cdot 0.5\ C_7H_8$ | $C_{22}H_{39}I_2LaO_3$ | $C_{64}H_{126}Al_4I_8La_6Si_6$ |
| $M_r$ [g/mol] | 4992.70 | 744.24 | 3020.76 |
| color/shape | colorless/column | colorless/plate | colorless/needle |
| crystal dimensions [mm] | 0.300 x 0.150 x 0.100 | 0.169 x 0.164 x 0.042 | 0.130 x 0.058 x 0.055 |
| crystal system | monoclinic | trigonal | orthorhombic |
| space group | $P2_1$ | $R\bar{3}$ | *Pbca* |
| a [Å] | 17.5518(3) | 42.437(2) | 24.1461(2) |
| b [Å] | 32.3785(6) | 42.437 | 11.8415(6) |
| c [Å] | 27.0466(6) | 9.155(3) | 35.1341(2) |
| α [°] | 90 | 90 | 90 |
| β [°] | 90.225(2) | 90 | 90 |
| γ [°] | 90 | 120 | 90 |
| V [Å³] | 15370.5(5) | 14278(1) | 10045.8(9) |
| Z | 4 | 18 | 4 |
| T [K] | 100(2) | 160(2) | 100(2) |
| λ [Å] | 0.71073 | 0.71073 | 0.71073 |
| $\rho_{calcd}$ [g/cm³] | 2.158 | 1.558 | 1.997 |
| $\mu$ [mm$^{-1}$] | 6.092 | 3.307 | 5.090 |
| F (000) | 9088 | 6444 | 5648 |
| θ range [°] | 2.586 – 29.573 | 0.960 – 26.661 | 2.001 – 28.126 |
| unique reflections | 68147 | 6688 | 12242 |
| observed reflections (I > 2σ) | 59962 | 3943 | 10216 |
| R1/*w*R2 (I > 2σ) | 0.0523/0.1196 | 0.0372/0.0664 | 0.0347/0.0653 |
| R1/*w*R2 (all data) | 0.0652/0.1266 | 0.0802/0.0766 | 0.0476/0.0696 |
| GOF | 1.038 | 0.763 | 1.147 |

**Table S1 (continued).** Crystallographic data for compounds **5a**, **6**, and **4b**

| | **5a** | **6** | **4b** |
|---|---|---|---|
| CCDC | 1992047 | 1992042 | 1992046 |
| formula | $C_{96}H_{156}I_{24}La_{12}Si_{12}$ | $C_{74}H_{114}I_{14}La_8Si_8$ | $C_{70}H_{140}Br_8La_6Si_6$ |
| $M_r$ [g/mol] | 6359.80 | 4116.25 | 2731.02 |
| color/shape | colorless/block | colorless/block | colorless/block |
| crystal dimensions [mm] | 0.150 x 0.149 x 0.109 | 0.323 x 0.264 x 0.224 | 0.418 x 0.141 x 0.115 |
| crystal system | trigonal | triclinic | triclinic |
| space group | $R3c$ | $P\bar{1}$ | $P\bar{1}$ |
| a [Å] | 25.024(5) | 13.287(5) | 12.869(2) |
| b [Å] | 25.024(5) | 15.242(6) | 17.812(3) |
| c [Å] | 59.440(2) | 16.924(7) | 24.039(4) |
| α [°] | 90 | 70.638(9) | 87.062(3) |
| β [°] | 90 | 78.664(13) | 82.387(3) |
| γ [°] | 120 | 69.889(9) | 79.387(3) |
| V [$Å^3$] | 32234(14) | 3023(2) | 5366.1(14) |
| Z | 6 | 1 | 2 |
| T [K] | 150(2) | 273(2) | 99(2) |
| λ [Å] | 0.71073 | 0.71073 | 0.71073 |
| $\rho_{calcd}$ [g/$cm^3$] | 1.966 | 2.261 | 1.690 |
| $\mu$ [$mm^{-1}$] | 5.868 | 6.444 | 5.444 |
| F (000) | 17136 | 1868 | 2636 |
| θ range [°] | 4.113 – 28.280 | 1.281 – 28.282 | 1.425 – 27.103 |
| unique reflections | 17746 | 14955 | 23669 |
| observed reflections (I > 2σ) | 14444 | 12561 | 19976 |
| R1/*w*R2 (I > 2σ) | 0.0425/0.0955 | 0.0534/0.1384 | 0.0670/0.1534 |
| R1/*w*R2 (all data) | 0.0707/0.1191 | 0.0649/0.1522 | 0.0879/0.1690 |
| GOF | 1.142 | 1.032 | 1.046 |

**Table S1 (continued).** Crystallographic data for compounds **5b**, **5b'**, and **7**

| | **5b** | **5b'** | **7** |
|---|---|---|---|
| CCDC | 1992040 | 1992048 | 1992041 |
| formula | $C_{96}H_{156}Br_{24}La_{12}Si_{12}$ | $C_{108}H_{184}Br_{24}La_{12}Si_{12}$ | $C_{90}H_{156}Al_2Br_{22}La_{10}Si_{10}$ |
| $M_r$ [g/mol] | 5232.04 | 5404.38 | 4720.12 |
| color/shape | colorless/cube | colorless/block | colorless/block |
| crystal dimensions [mm] | 0.354 x 0.261 x 0.204 | 0.252 x 0.170 x 0.126 | 0.194 x 0.161 x 0.143 |
| crystal system | monoclinic | monoclinic | triclinic |
| space group | $C2/c$ | $P2_1/c$ | $P\bar{1}$ |
| a [Å] | 29.5915(18) | 22.9195(17) | 18.6798(8) |
| b [Å] | 18.9561(12) | 29.214(2) | 19.3010(8) |
| c [Å] | 27.7599(17) | 25.8590(19) | 22.8183(10) |
| α [°] | 90 | 90 | 82.0620(10) |
| β [°] | 100.1190(10) | 101.3160(10) | 88.7740(10) |
| γ [°] | 90 | 90 | 62.3840(10) |
| V [Å³] | 15329.4(16) | 16978(2) | 7211.3(5) |
| Z | 4 | 4 | 2 |
| T [K] | 100(2) | 100(2) | 173(2) |
| λ [Å] | 0.71073 | 0.71073 | 0.71073 |
| $\rho_{calcd}$ [g/cm³] | 2.267 | 2.114 | 2.174 |
| $\mu$ [mm$^{-1}$] | 9.647 | 8.714 | 9.116 |
| F (000) | 9696 | 10096 | 4404 |
| θ range [°] | 1.870 – 28.282 | 1.682 – 28.340 | 1.203 – 29.547 |
| unique reflections | 19039 | 42322 | 40138 |
| observed reflections (I > 2σ) | 17083 | 405987 | 33091 |
| R1/*w*R2 (I > 2σ) | 0.0260/0.0543 | 0.0282/0.0490 | 0.0284/0.0597 |
| R1/*w*R2 (all data) | 0.0320/0.0560 | 0.0478/0.0539 | 0.0415/0.0644 |
| GOF | 1.093 | 1.004 | 1.011 |

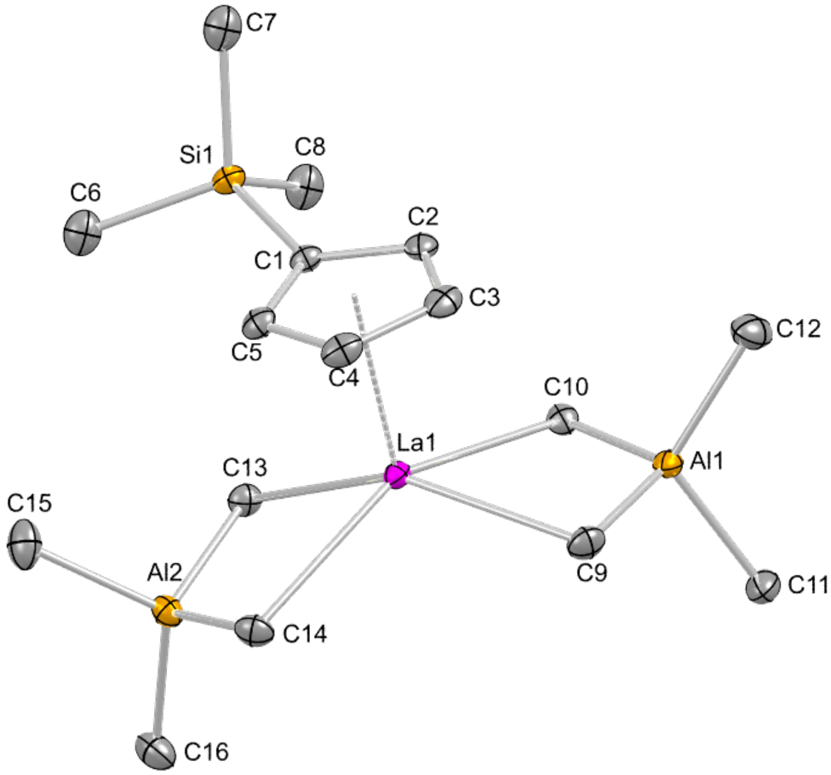


**Figure S20.** Crystal structure of Cp'La($AlMe_4$)$_2$ (**1b**) with atomic displacement parameters set at the 50% probability level. Hydrogen atoms are omitted for clarity. Selected interatomic distances [Å] and angles [°]: La1–C1 2.821(3); La1–C2 2.806(3); La1–C3 2.784(3); La1–C4 2.759(3); La1–C5 2.774(3); La1···Ct1 2.514; La1–C9 2.759(4); La1–C10 2.738(3); La1···Al1 3.258(1); La1–C13 2.710(3); La1–C14 2.752(4); La1···Al2 3.277(1); La1···C11' 3.267(4); C1–C2 1.428(4); C2–C3 1.410(4); C3–C4 1.418(5); C4–C5 1.409(4); C1–C5 1.426(4); C1–Si1 1.876(3); C1–C2–C3 109.3(3); C2–C3–C4 107.9(3); C3–C4–C5 107.4(3); C4–C5–C1 109.7(1); C5–C1–C2 105.6(2); Si1–C1–La1 126.58(2); C9–La1–C10 78.0(1); C13–La1–C14 78.1(1); C9–Al1–C10 113.10(2); C11–Al1–C12 114.51(2); C13–Al2–C14 112.16(2); C15–Al2–C16 115.64(2).

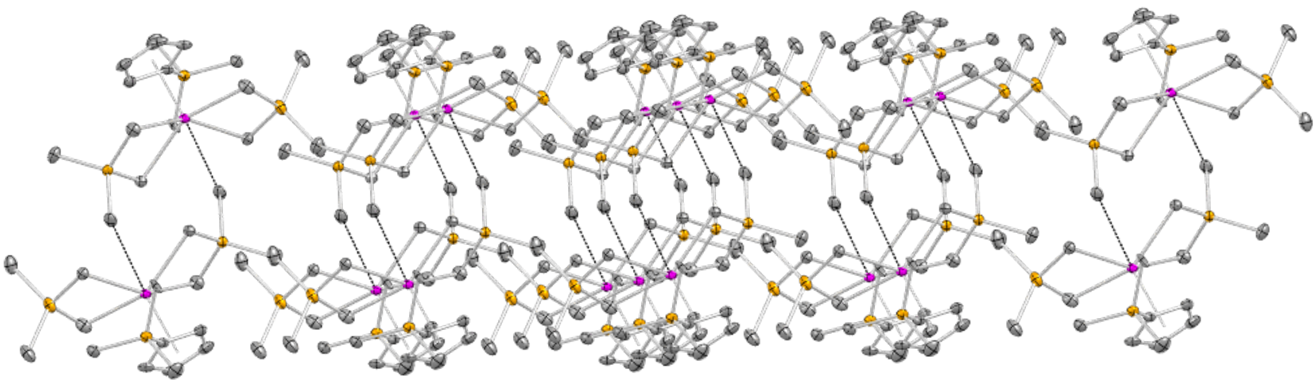

**Figure S21.** Crystal packing of **1b**. Each lanthanum metal center interacts with a methyl group of a neighboring molecule, resulting in a dimeric arrangement.

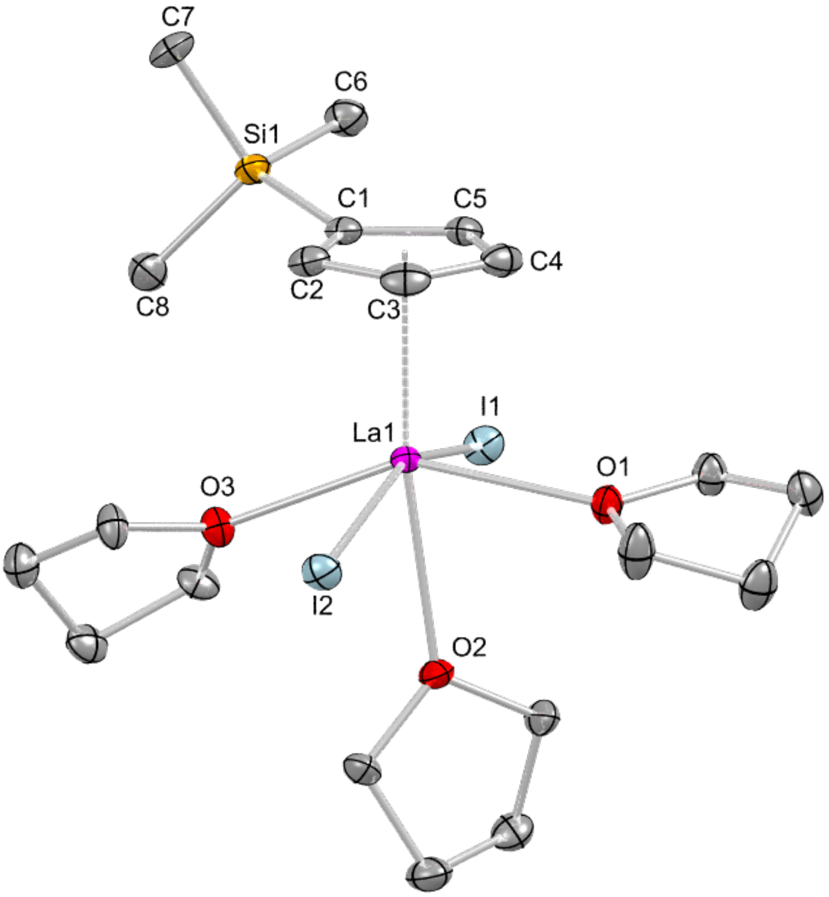


**Figure S22.** Crystal structure of [Cp'La$I_2$(thf)$_3$] with atomic displacement parameters set at the 50% probability level. Hydrogen atoms are omitted for clarity. Selected interatomic distances [Å] and angles [°]: La1–C1 2.821(4); La1–C2 2.811(4); La1–C3 2.808(5); La1–C4 2.813(5); La1–C5 2.804(4); La1···Ct1 2.542; La1–I1 3.1909(8); La1–I2 3.2421(8); La1–O1 2.513(3); La1–O2 2.607(3); La1–O3 2.532(3); C1–C2 1.431(7); C2–C3 1.406(7); C3–C4 1.408(7); C4–C5 1.402(6); C1–C5 1.423(6); C1–Si1 1.867(5); C1–C2–C3 109.0(4); C2–C3–C4 108.1(4); C3–C4–C5 107.8(4); C4–C5–C1 109.6(4); C5–C1–C2 105.5(4); Si1–C1–La1 126.6(2); I1–La1–I2 158.590(2); I1–La1–O1 87.70(8); I1–La1–O2 80.93(8); I1–La1–O3 86.16(8); O1–La1–O2 72.65(2); O1–La1–O3 148.27(2); O2–La1–O3 75.65(2); Ct1···La1–I1 101.69; Ct1···La1–I2 99.70; Ct1···La1–O1 98.43; Ct1···La1–O2 170.70; Ct1···La1–O3 113.29.

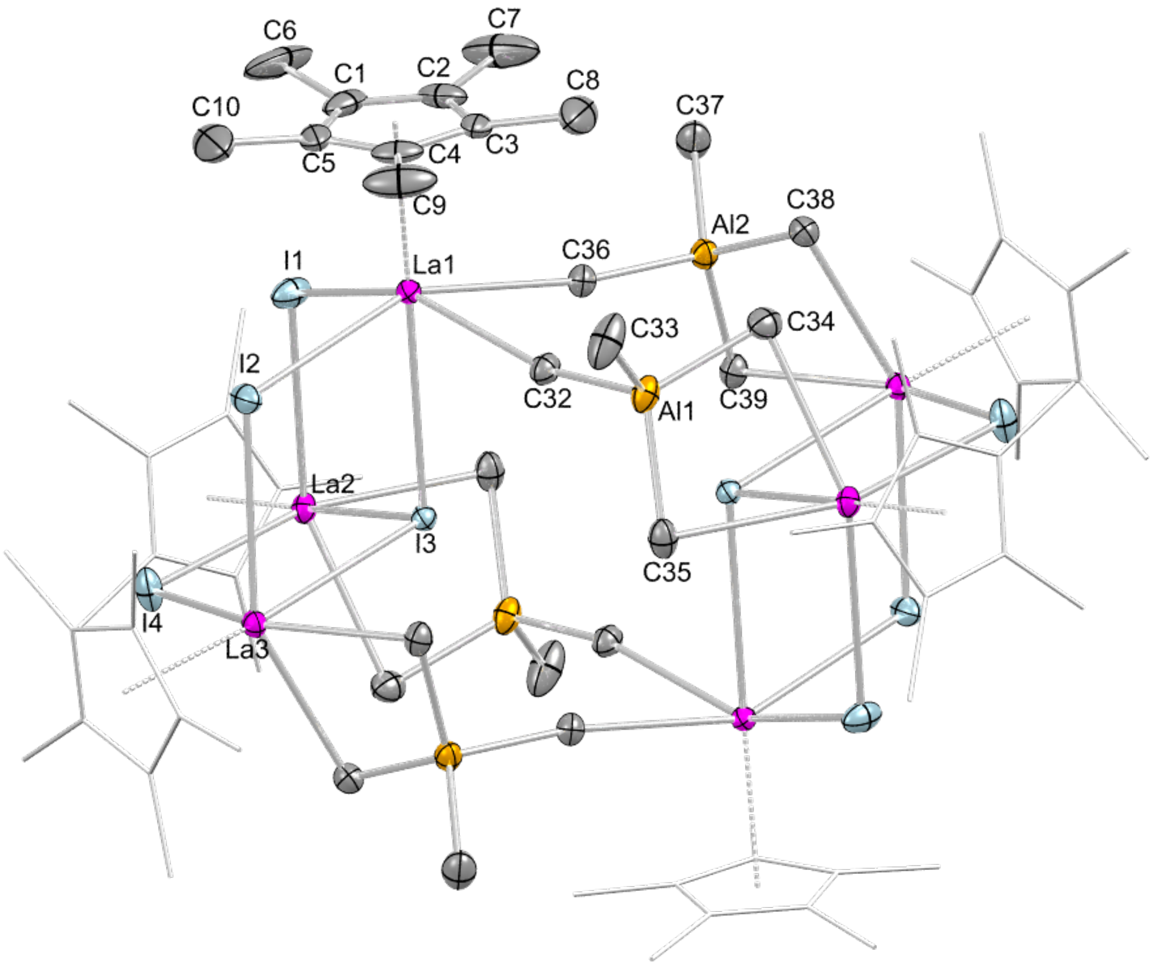


**Figure S23.** Crystal structure of [$Cp^*{}_6La_6I_8(AlMe_4)_4$] (**2**) with atomic displacement parameters set at the 50% probability level. Hydrogen atoms are omitted for clarity. Selected interatomic distances [Å] and angles [°]: La1–C1 2.797(4); La1–C2 2.794(4); La1–C3 2.758(4); La1–C4 2.748(4); La1–C5 2.769(4); La1···Ct1 2.501; La1–C32 2.966(3); La1–C36 2.954(4); La1–I1 3.2096(4); La1–I2 3.2323(5); La1–I3 3.3455(4); La2–I1 3.2605(4); La2–I3 3.3981(4); La2–I4 3.2379(4); La2–C34 2.771(4); La2–C35 2.777(4); La2···Al1 3.3159(2); C1–C2 1.383(6); C2–C3 1.404(6); C3–C4 1.419(6); C4–C5 1.416(6); C1–C5 1.395(7); C1–C6 1.525(6); C1–C2–C3 108.3(4); C2–C3–C4 107.9(4); C3–C4–C5 106.8(4); C4–C5–C1 108.0(4); C5–C1–C2 109.0(4); C32–La1–C36 82.08(1); C34–La2–C35 76.06(2); C34–Al1–C35 112.94(2); C38–La3–C39 76.13(2); C38–Al2–C39 112.65(2); C32–La1–I2 86.05(7); C36–La1–I1 86.81(8); I1–La1–I2 94.909(2); I1–La1–I3 76.760(2); I2–La1–I3 76.072(2); La1–I1–La2 106.936(2); La1–I3–La2 100.876(2); La1–I2–La3 108.047(2); La1–I3–La3 101.265(2); La2–I3–La3 100.126(2); La2–I4–La3 108.169(2).

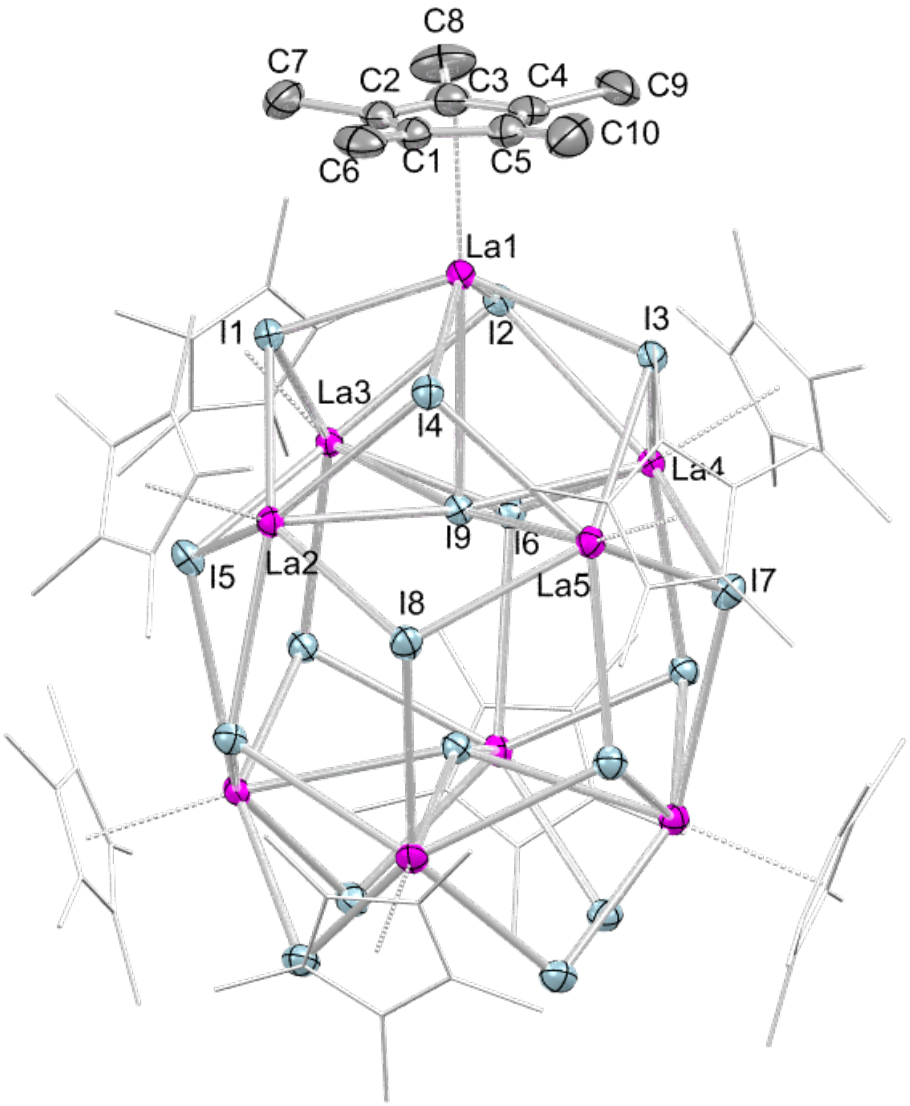


**Figure S24.** Crystal structure of [Cp*LaI$_2$]$_9$ **(3)** with atomic displacement parameters set at the 50% probability level. Hydrogen atoms are omitted for clarity. The Cp* ligands (except for one) are represented by a wireframe model for improved visualization. Selected interatomic distances [Å] and angles [°]: La1–C1 2.76(2); La1–C2 2.76(2); La1–C3 2.76(2); La1–C4 2.73(2); La1–C5 2.74(2); La1···Ct1 2.456; La1–I1 3.1894(2); La1–I2 3.2012(2); La1–I3 3.2161(2); La1–I4 3.2170(2); La2–I1 3.4478(2); La2–I4 3.4631(2); La2–I5 3.3423(2); La2–I8 3.2991(2); La2–I9 3.5294(2); C1–C2 1.42(3); C2–C3 1.42(3); C3–C4 1.46(4); C4–C5 1.39(3); C1–C5 1.44(3); C1–C6 1.50(3); C1–C2–C3 109(2); C2–C3–C4 106(2); C3–C4–C5 109(2); C4–C5–C1 108(2); C5–C1–C2 108(2); I1–La1–I2 83.45(4); I1–La1–I3 138.90(5); I1–La1–I9 69.45; I1–La2–I4 76.50(4); I1–La2–I5 68.27(4); I1–La2–I8 140.17(4); I1–La2–I9 69.14(3); I5–La2–I8 129.16(5); I5–La2–I9 78.56(4); La1–I1–La2 95.66(4); La1–I4–La2 94.86(4); La1–I9–La2 85.49; La2–I1–La3 93.90(4); La2–I5–La3 98.57(4); La2–I9–La3 90.34(4); La2–I9–La4 171.11(4).

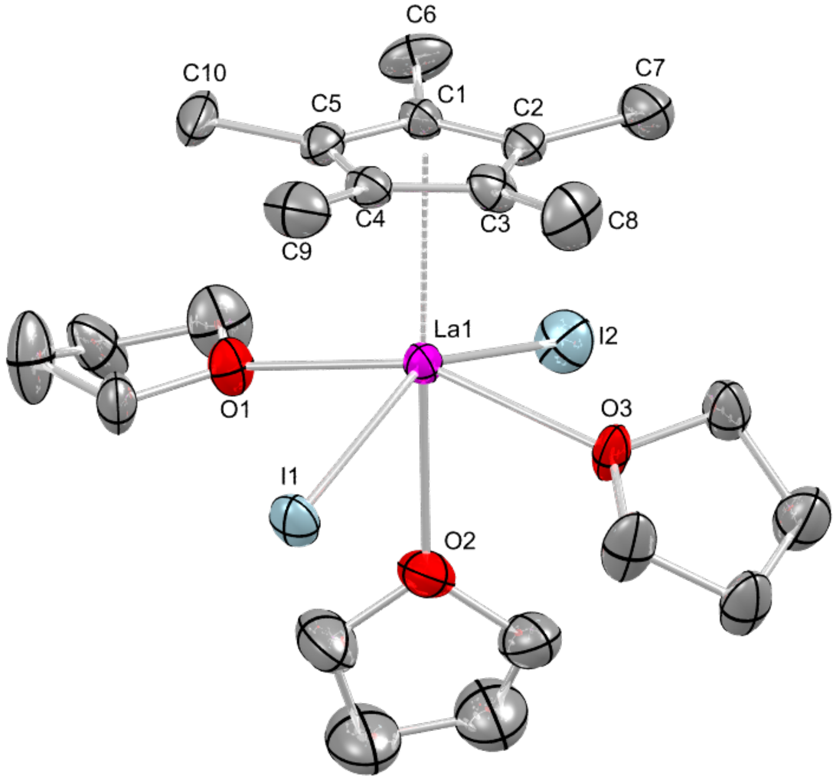


**Figure S25.** Crystal structure of [Cp*La$I_2$(thf)$_3$] with atomic displacement parameters set at the 50% probability level. Hydrogen atoms are omitted for clarity. Selected interatomic distances [Å] and angles [°]: La1–C1 2.795(5); La1–C2 2.804(6); La1–C3 2.815(6); La1–C4 2.820(6); La1–C5 2.820(5); La1···Ct1 2.544; La1–I1 3.1974(9); La1–I2 3.2452(7); La1–O1 2.533(4); La1–O2 2.626(7); La1–O3 2.546(4); C1–C2 1.409(7); C2–C3 1.421(7); C3–C4 1.409(8); C4–C5 1.438(7); C1–C5 1.405(4); C1–C6 1.516(7); C1–C2–C3 108.7(5); C2–C3–C4 106.7(5); C3–C4–C5 109.2(5); C4–C5–C1 106.4(5); C5–C1–C2 109.0(5); I1–La1–I2 150.084(2); I1–La1–O1 86.76(9); I1–La1–O2 76.8(2); I1–La1–O3 85.15(9); O1–La1–O2 78.27(2); O1–La1–O3 154.54(2); O2–La1–O3 76.39(2); Ct1···La1–I1 105.64; Ct1···La1–I2 104.14; Ct1···La1–O1 102.14; Ct1···La1–O2 177.54; Ct1···La1–O3 103.28.

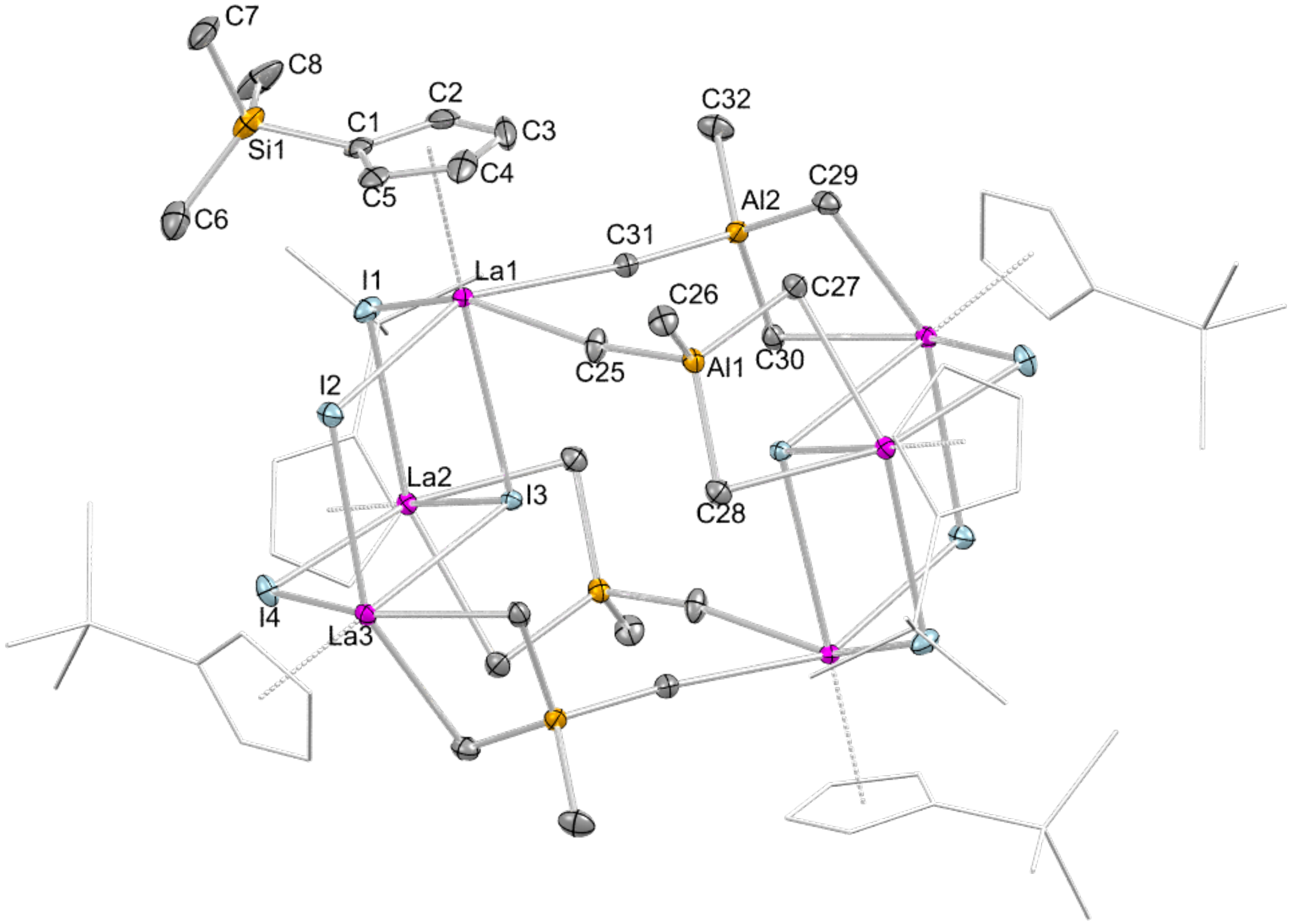


**Figure S26.** Crystal structure of [$Cp'_6La_6I_8(AlMe_4)_4$] (**4a**) with atomic displacement parameters set at the 50% probability level. Hydrogen atoms are omitted for clarity. The Cp' ligands (except for one) are represented by a wireframe model for improved visualization. Selected interatomic distances [Å] and angles [°]: La1–C1 2.793(5); La1–C2 2.742(6); La1–C3 2.711(6); La1–C4 2.760(6); La1–C5 2.785(5); La1···Ct1 2.484; La1–C25 2.920(6); La1–C31 2.996(5); La1–I1 3.2163(5); La1–I2 3.1893(5); La1–I3 3.3435(4); La2–I1 3.2231(5); La2–I3 3.3685(5); La2–I4 3.2211(5); La2–C27 2.818(6); La2–C28 2.729(6); La2···Al1 3.2865(2); C1–C2 1.436(8); C2–C3 1.428(1); C3–C4 1.366(1); C4–C5 1.395(8); C1–C5 1.424(8); C1–Si1 1.869(6); C1–C2–C3 107.9(6); C2–C3–C4 108.0(5); C3–C4–C5 109.6(6); C4–C5–C1 108.9(6); C5–C1–C2 105.6(5); Si1–C1–La1 129.4(3); C25–La1–C31 79.8(2); C27–La2–C28 76.12(2); C27–Al1–C28 114.0(2); C29–La3–C30 76.09(2); C29–Al2–C30 113.1(2); C25–La1–I2 86.6(1); C31–La1–I1 84.7(1); I1–La1–I2 99.078(2); I1–La1–I3 76.899(2); I2–La1–I3 77.660(2); La1–I1–La2 106.326(2); La1–I3–La2 100.318(2); La1–I2–La3 106.670(2); La1–I3–La3 99.039(2); La2–I3–La3 99.962(2); La2–I4–La3 106.822(2).

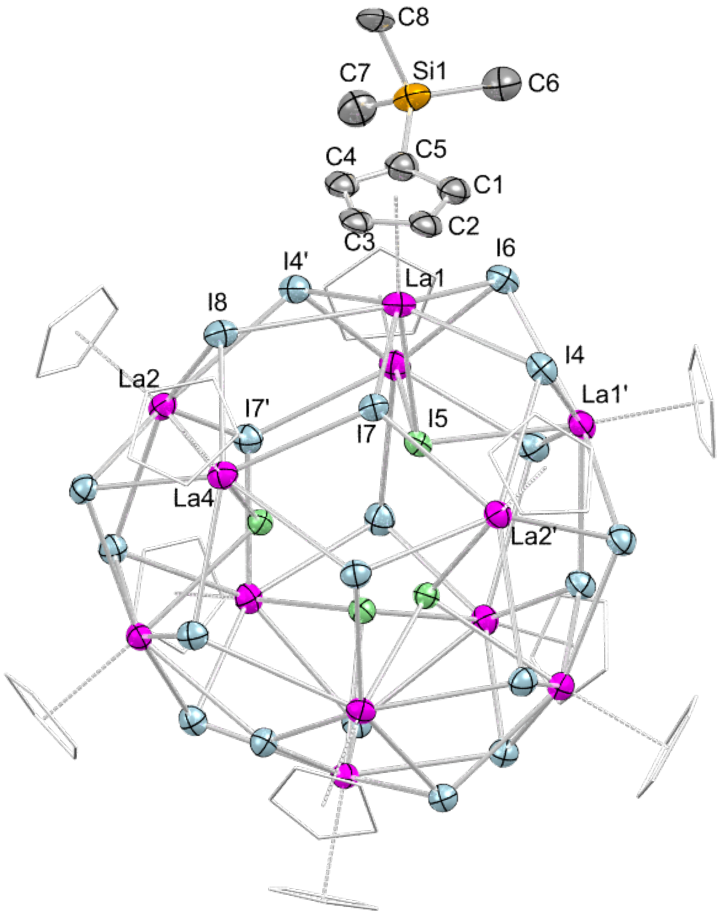


**Figure S27.** Crystal structure of $[Cp'LaI_2]_{12}$ (**5a**) with atomic displacement parameters set at the 50% probability level. Hydrogen atoms are omitted for clarity. The Cp' ligands (except for one) are represented by a wireframe model for improved visualization. Selected interatomic distances [Å] and angles [°]: La1–C1 2.71(4); La1–C2 2.67(5); La1–C3 2.70(4); La1–C4 2.76(3); La1–C5 2.75(4); La1···Ct1 2.453; La1–I4 3.2766(2); La1–I4' 3.2114(2); La1–I5 3.2811(2); La1–I6 3.3663(2); La1–I7 3.2669(2); La1–I8 3.6913(2); C1–C2 1.37(2); C2–C3 1.38(2); C3–C4 1.37(2); C4–C5 1.38(2); C1–C5 1.38(2); C5–Si1 1.90(3); C1–C2–C3 108.1(2); C2–C3–C4 107.9(2); C3–C4–C5 108.1(2); C4–C5–C1 107.9(2); C5–C1–C2 108.0(2); Si1–C5–La1 131.8(2); I4–La1–I6 76.70; I4'-La1–I6 77.57; I4–La1–I7 69.55(4); I4'–La1–I8 65.44(4); I7–La1–I8 64.59(4); La1–I4–La1' 93.78(5); La1–I5–La1' 92.41(4); La1–I6–La1' 89.43(4); La1–I4–La2' 105.17(4); La1–I8–La2 102.62(4); La1–I7–La4 114.70(4); La1–I8–La4 104.40(4).

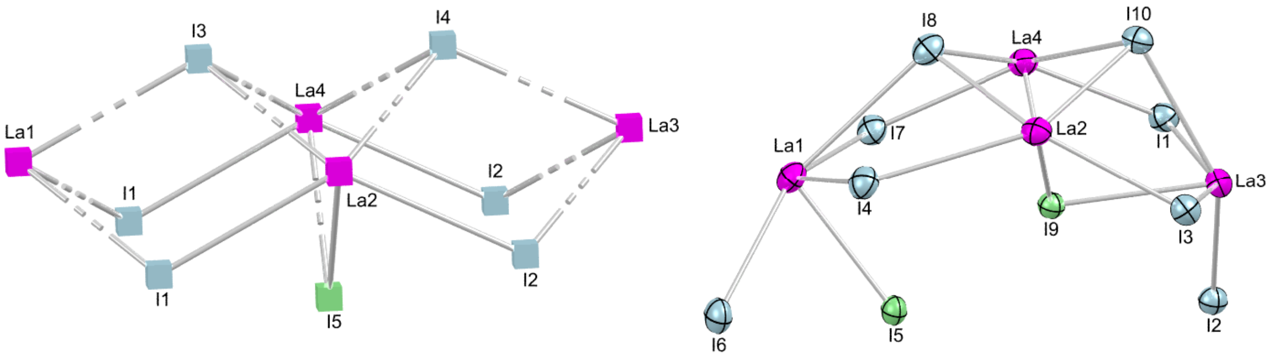


**Figure S28.** Comparison of the solid-state structure of $LaI_3$ (adapted from Zachariasen,[11] left) with the asymmetric unit of **5a** (right).

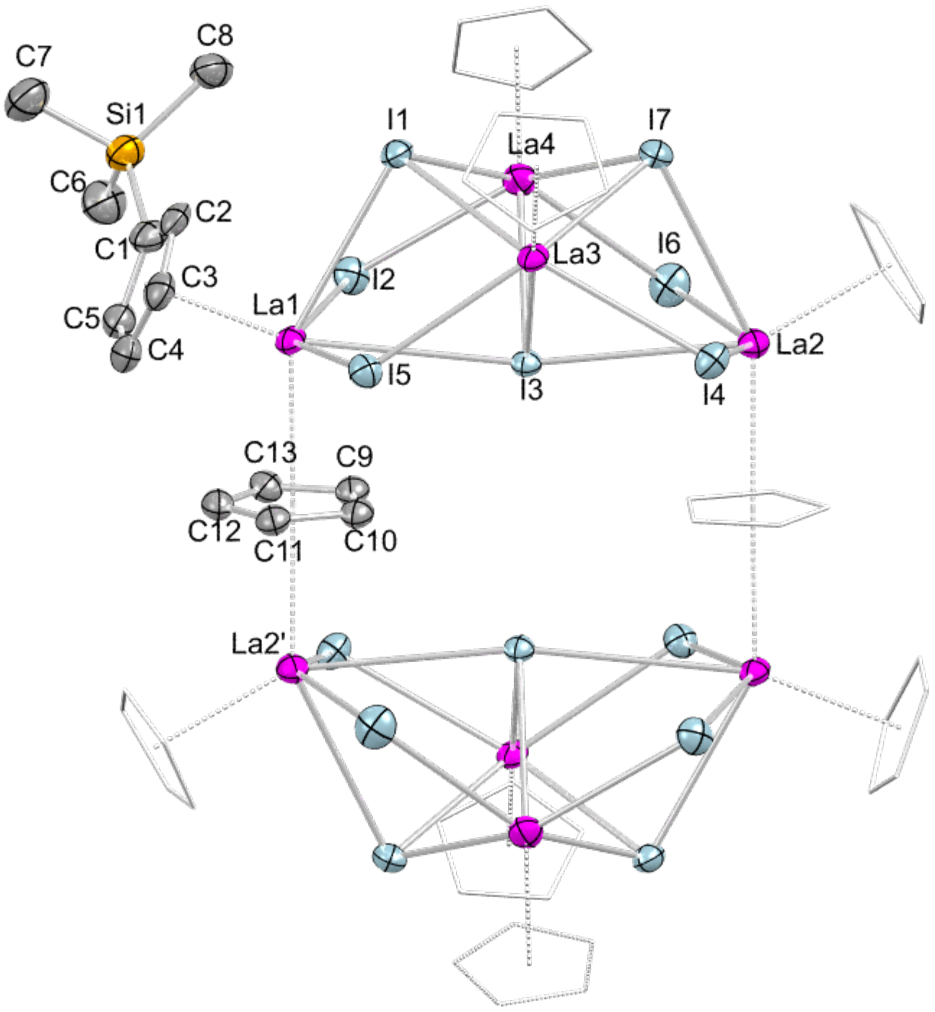


**Figure S29.** Crystal structure of [($\mu$-Cp)$_2$Cp'$_8$La$_8$I$_{14}$] (**6**) with atomic displacement parameters set at the 50% probability level. Hydrogen atoms are omitted for clarity. The Cp' and Cp ligands (except for one) are represented by a wireframe model for improved visualization.

**Table S2.** Selected interatomic distances and angles for **6**.

| **Bond lengths [Å]** | | **Bond angles [°]** | |
|---|---|---|---|
| La1–C1 | 2.798(1) | C1–C2–C3 | 109.3(8) |
| La1–C2 | 2.780(8) | C2–C3–C4 | 107.7(9) |
| La1–C3 | 2.785(9) | C3–C4–C5 | 108.3(9) |
| La1–C4 | 2.746(9) | C4–C5–C1 | 108.5(8) |
| La1–C5 | 2.750(9) | C5–C1–C2 | 106.2(9) |
| La1···Ct(Cp') | 2.509 | Si1-C1-La1 | 132.1(5) |
| La1–C9 | 2.951(8) | C9–C10–C11 | 107.7(8) |
| La1–C10 | 2.915(8) | C10–C11–C12 | 107.8(8) |
| La1–C11 | 2.874(8) | C11–C12–C13 | 108.3(8) |
| La1–C12 | 2.912(8) | C12–C13–C9 | 108.6(8) |
| La1–C13 | 2.954(8) | C13–C9–C10 | 107.5(8) |
| La1···Ct($\mu$-Cp) | 2.668 | Ct(Cp')···La1···Ct($\mu$-Cp) | 114.19 |

| | | | |
|---|---|---|---|
| C1–C2 | 1.406(2) | La1···Ct($\mu$-Cp)···La2' | 175.11 |
| C2–C3 | 1.394(2) | La1–I1–La3 | 90.58(2) |
| C3–C4 | 1.384(2) | La1–I5–La3 | 95.60(2) |
| C4–C5 | 1.403(2) | La1–I1–La4 | 91.10(4) |
| C1–C5 | 1.406(2) | La1–I2–La4 | 95.80(4) |
| C1–Si1 | 1.865(1) | La2–I3–La3 | 88.08(2) |
| C9–C10 | 1.414(2) | La2–I4–La3 | 95.00(4) |
| C10–C11 | 1.414(2) | La2–I6–La4 | 94.03(3) |
| C11–C12 | 1.399(2) | La2–I7–La4 | 89.25(3) |
| C12–C13 | 1.396(2) | La3–I1–La4 | 91.17(2) |
| C9–C13 | 1.400(2) | La3–I3–La4 | 90.26(3) |
| La1–I1 | 3.4481(2) | I1–La1–I2 | 76.99(2) |
| La1–I2 | 3.2616(2) | I2–La1–I5 | 140.82(2) |
| La1–I3 | 3.734(1) | I3–La2–I4 | 75.22(3) |
| La1–I5 | 3.2727(2) | I3–La2–I6 | 70.79(3) |
| La2–I3 | 3.5895(2) | I1–La3–I3 | 74.14(3) |
| La2–I4 | 3.2485(2) | I1–La3–I4 | 146.42(2) |
| La2–I6 | 3.2924(2) | I1–La3–I5 | 79.27(2) |
| La2–I7 | 3.4739(2) | I1–La3–I7 | 76.35(2) |
| | | I3–La3–I4 | 79.35(3) |

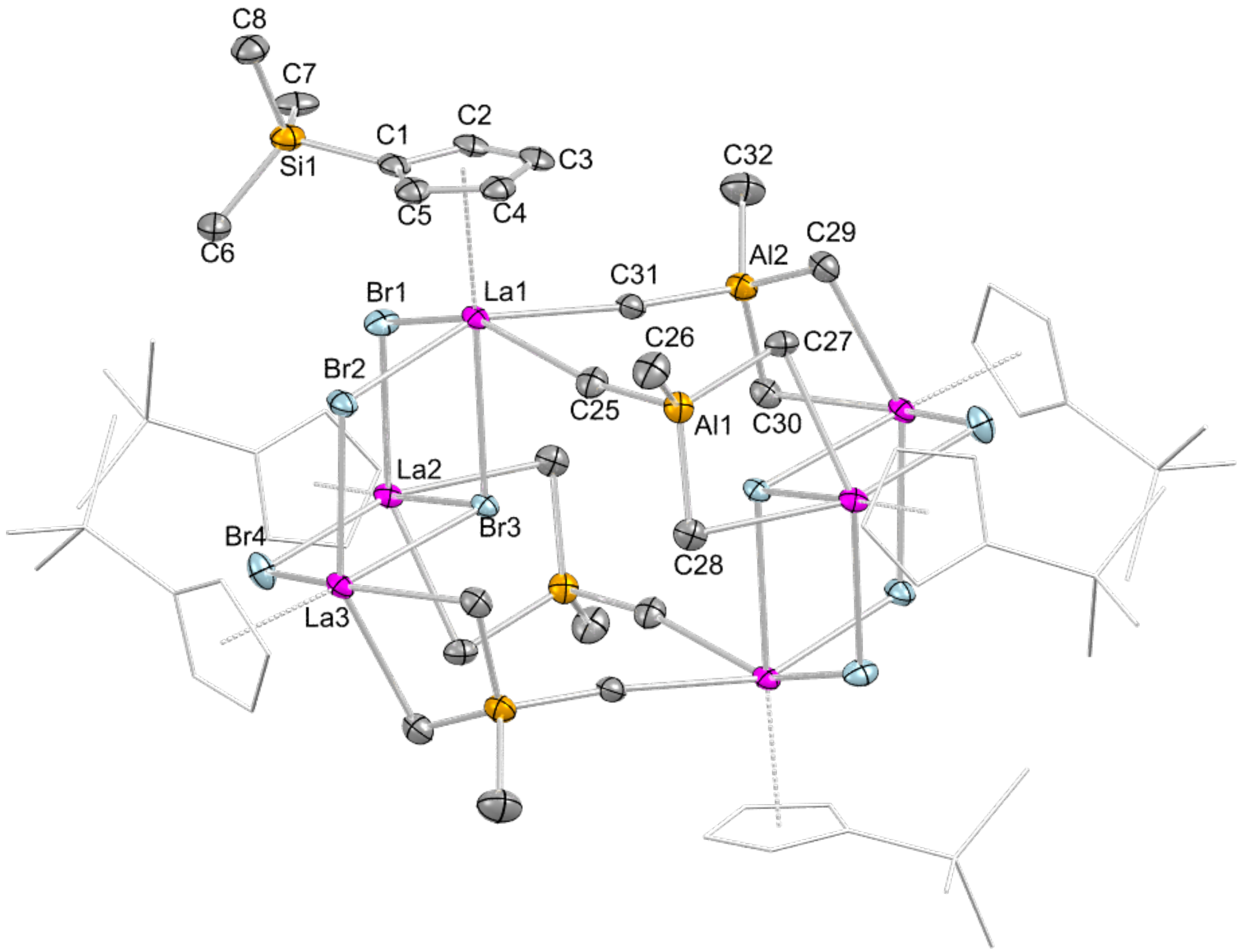


**Figure S30.** Crystal structure of [$Cp'_6La_6Br_8(AlMe_4)_4$] (**4b**) with atomic displacement parameters set at the 50% probability level. Hydrogen atoms are omitted for clarity. The Cp' ligands (except for one) are represented by a wireframe model for improved visualization. Selected interatomic distances [Å] and angles [°]: La1–C1 2.780(2); La1–C2 2.775(2); La1–C3 2.794(2); La1–C4 2.772(2); La1–C5 2.777(2); La1···Ct1 2.505; La1–C25 2.961(2); La1–C31 2.961(5); La1–Br1 2.9740(2); La1–Br2 2.9681(2); La1–Br3 3.1473(2); La2–Br1 2.9953(2); La2–Br3 3.1917(2); La2–Br4 2.9967(2); La2–C27 2.763(2); La2–C28 2.773(2); La2···Al1 3.2865(2); C1–C2 1.383(2); C2–C3 1.398(2); C3–C4 1.444(2); C4–C5 1.385(2); C1–C5 1.445(2); C1–Si1 1.873(2); C1–C2–C3 113.0(2); C2–C3–C4 105.0(2); C3–C4–C5 107.8(2); C4–C5–C1 109.9(2); C5–C1–C2 104.2(2); Si1–C1–La1 127.4(5); C25–La1–C31 82.3(3); C27–La2–C28 76.8(4); C27–Al1–C28 114.5(5); C29–La3–C30 76.7(3); C29–Al2–C30 113.6(5); C25–La1–Br2 86.0(2); C31–La1–Br1 87.7(2); Br1–La1–Br2 94.10(4); Br1–La1–Br3 75.34(3); Br2–La1–Br3 75.11(3); La1–Br1–La2 109.59(4); La1–Br3–La2 100.60(4); La1–Br2–La3 109.91(4); La1–Br3–La3 100.89(3); La2–Br3–La3 98.79(3); La2–Br4–La3 107.77(4).

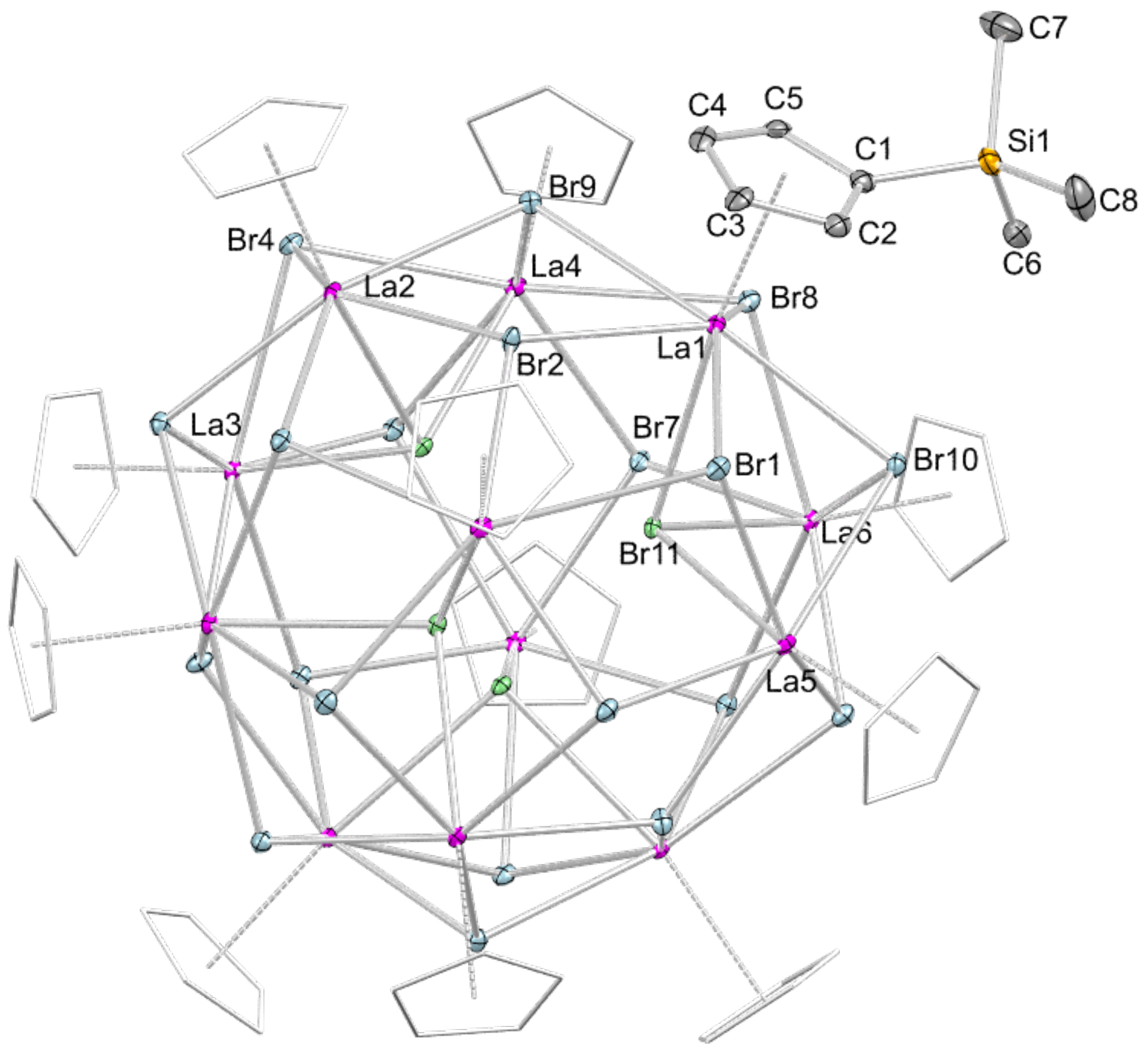


**Figure S31.** Crystal structure of [Cp'LaBr$_2$]$_{12}$ (**5b**) with atomic displacement parameters set at the 50% probability level. Hydrogen atoms are omitted for clarity. The Cp' ligands (except for one) are represented by a wireframe model for improved visualization. Selected interatomic distances [Å] and angles [°]: La1–C1 2.827(3); La1–C2 2.761(4); La1–C3 2.720(3); La1–C4 2.736(3); La1–C5 2.781(3); La1···Ct1 2.489; La1–Br1 3.1284(4); La1–Br2 3.1013(4); La1–Br8 3.0178 (4); La1–Br9 3.3451(4); La1–Br10 3.1461(4); La1–Br11 3.0972(4); C1–C2 1.425(5); C2–C3 1.424(5); C3–C4 1.405(5); C4–C5 1.411(6); C1–C5 1.425(5); C1–Si1 1.876(4); C1–C2–C3 109.1(3); C2–C3–C4 107.4(3); C3–C4–C5 108.4(3); C4–C5–C1 109.1(3); C5–C1–C2 106.0(3); Si1–C1–La1 133.00(2); Br1–La1–Br2 67.673(1); Br1–La1–Br10 75.057(1); Br2–La1–Br9 63.476(9); Br8–La1–Br9 68.432(1); Br8–La1–Br10 77.826(1); La1–Br2–La2 115.564(2); La1–Br8–La4 102.820(2); La1–Br9–La4 105.045(2); La1–Br1–La5 95.033(2); La1–Br10–La5 91.399(2); La1–Br11–La5 93.014(2); La1–Br8–La6 94.334(1); La1–Br10–La6 90.370(1); La1–Br11–La6 93.068(2).

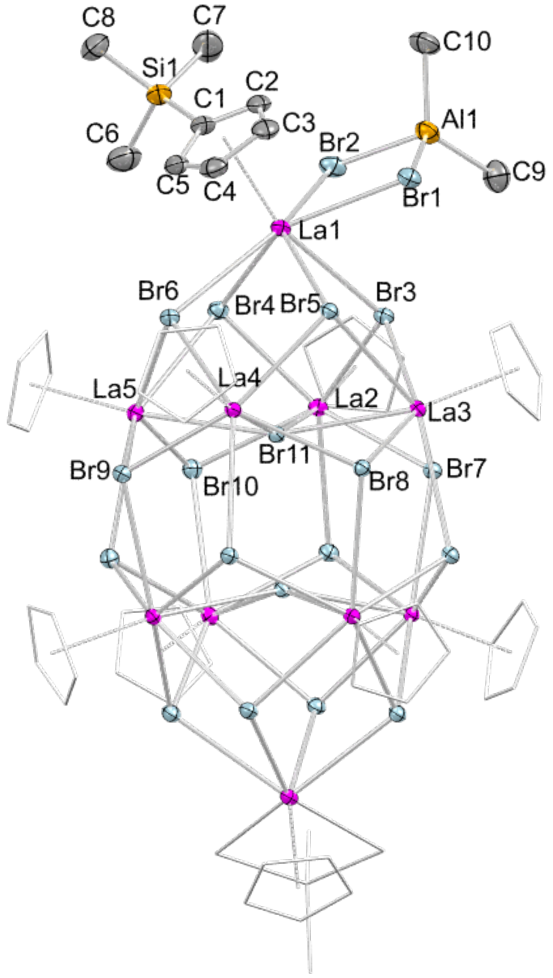


**Figure S32.** Crystal structure of [$Cp_5$'$La_5Br_{11}$($AlMe_2$)$]_2$ (**7**) with atomic displacement parameters set at the 50% probability level. Hydrogen atoms are omitted for clarity. The Cp' ligands (except for one) are represented by a wireframe model for improved visualization. Selected interatomic distances [Å] and angles [°]: La1–C1 2.803(4); La1–C2 2.764(4); La1–C3 2.757(4); La1–C4 2.753(4); La1–C5 2.766(4); La1···Ct1 2.494; La1–Br1 3.0945(4); La1–Br2 3.0595(5); La1···Al1 4.056; La1–Br3 3.2027(4); La1–Br4 3.1878(4); La1–Br5 3.1450(4); La1–Br6 3.3167(4); La2–Br3 3.0571(4); La2–Br4 3.1344(4); La2–Br7 3.1024(4); La2–Br10 3.0678(4); La2–Br11 3.2738(4); C1–C2 1.420(6); C2–C3 1.403(7); C3–C4 1.415(6); C4–C5 1.402(7); C1–C5 1.424(6); C1–Si1 1.877(5); Al1–Br1 2.4176(2); Al1–Br2 2.4494(2); Al1–C9 1.938(5); Al1–C10 1.936(5); C1–C2–C3 109.9(4); C2–C3–C4 107.6(4); C3–C4–C5 107.3(4); C4–C5–C1 110.1(4); C5–C1–C2 105.0(4); Si1–C1–La1 129.6(2); Br1–La1–Br2 73.474(2); Br1–Al1–Br2 98.28(5); La1–Br1–Al1 93.95(3); La1–Br2–Al1 94.18(3); Br1–La1–Br3 71.090(2); Br1–La1–Br4 139.239(2); Br1–La1–Br5 73.396(2); Br1–La1–Br6 140.703(2); Br3–La1–Br4 71.643(1); Br3–La1–Br5 73.381(1); Br3–La1–Br6 107.407(2)

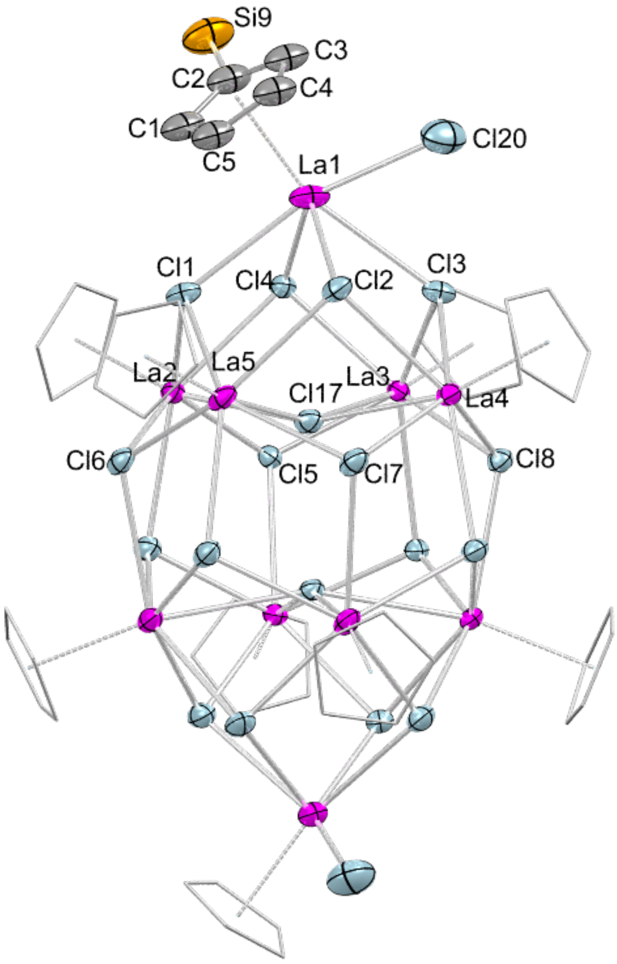


**Figure S33.** Connectivity of [Cp'LaCl$_2$]$_{10}$ (**8**) with atomic displacement parameters set at the 50% probability level. Hydrogen atoms are omitted for clarity. The Cp' ligands (except for one) are represented by a wireframe model for improved visualization. Due to disorder, the methyl groups of the Cp' ligands are omitted for clarity. Cell constants: a = 34.348(4), b = 16.020(2), 29.448(6), ß = 121.816(2), space group: *Cc*.

# IR Spectroscopy

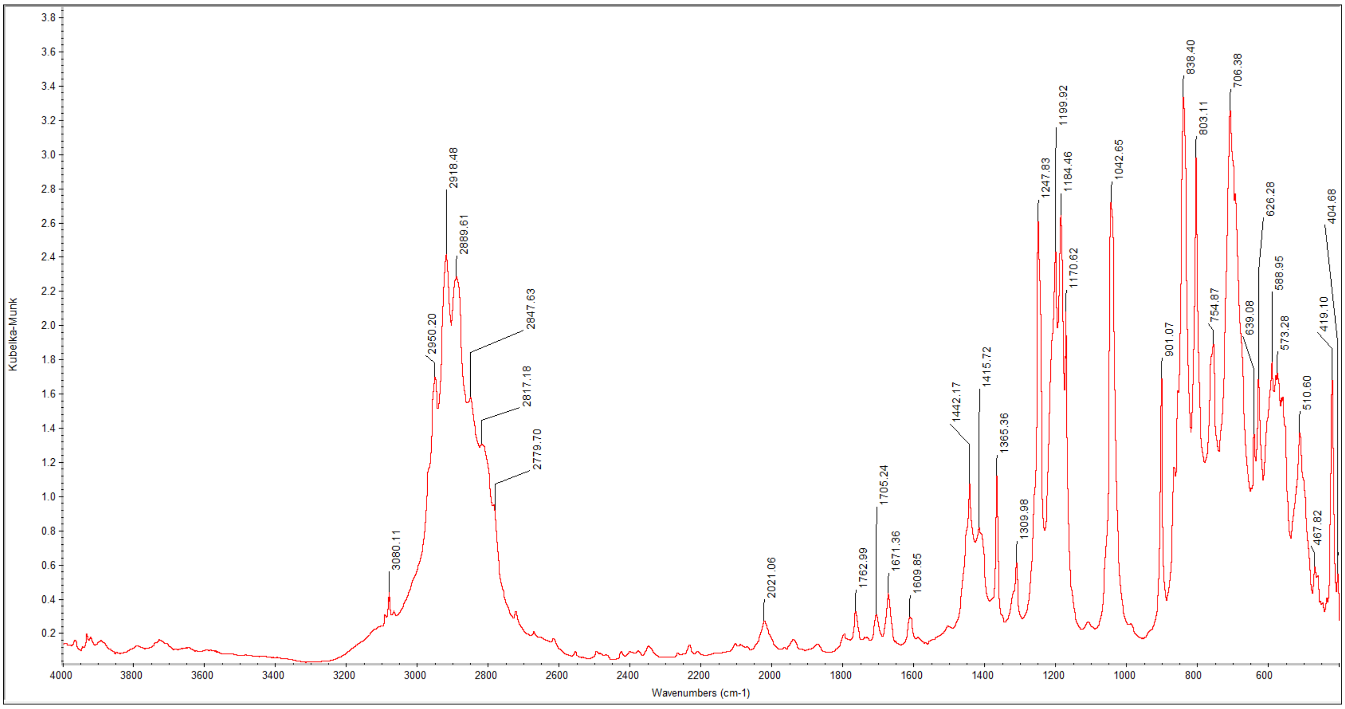


**Figure S34.** DRIFT spectrum of Cp'La($AlMe_4$)$_2$ (**1b**).

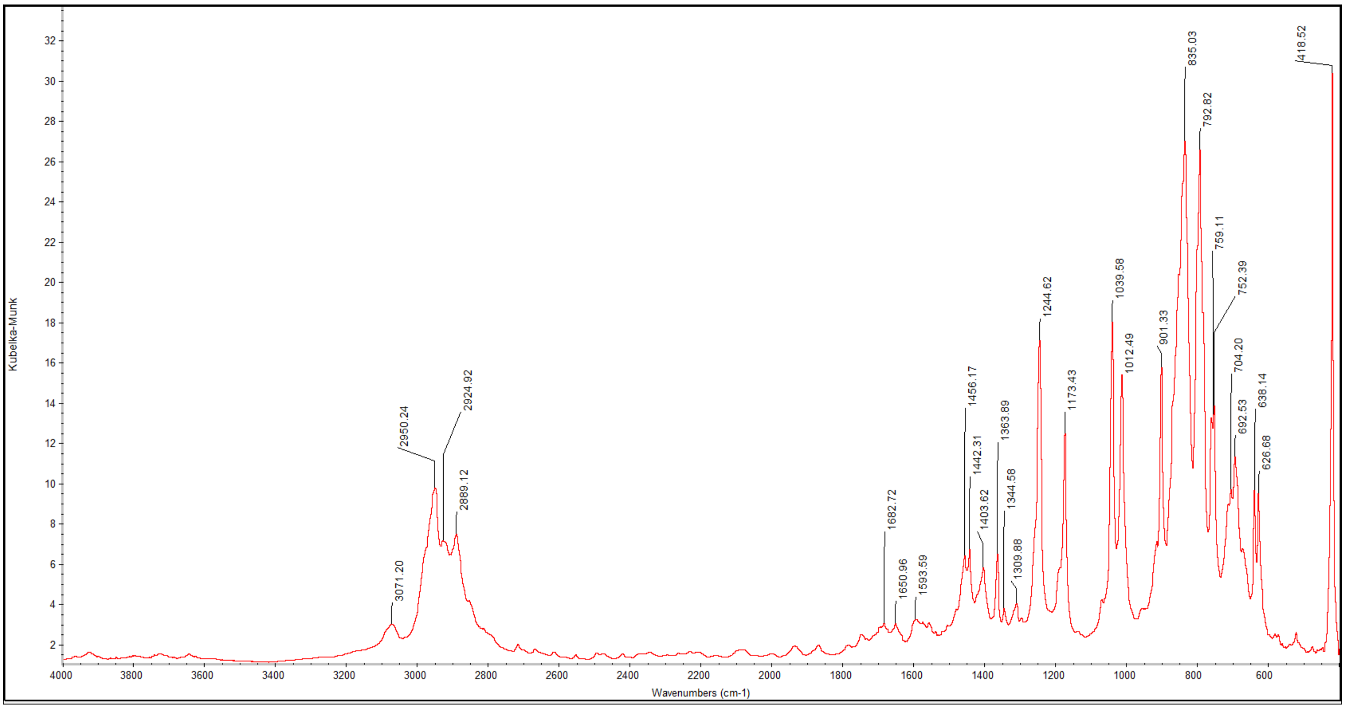


**Figure S35.** DRIFT spectrum of [Cp'La$I_2$(thf)$_3$].

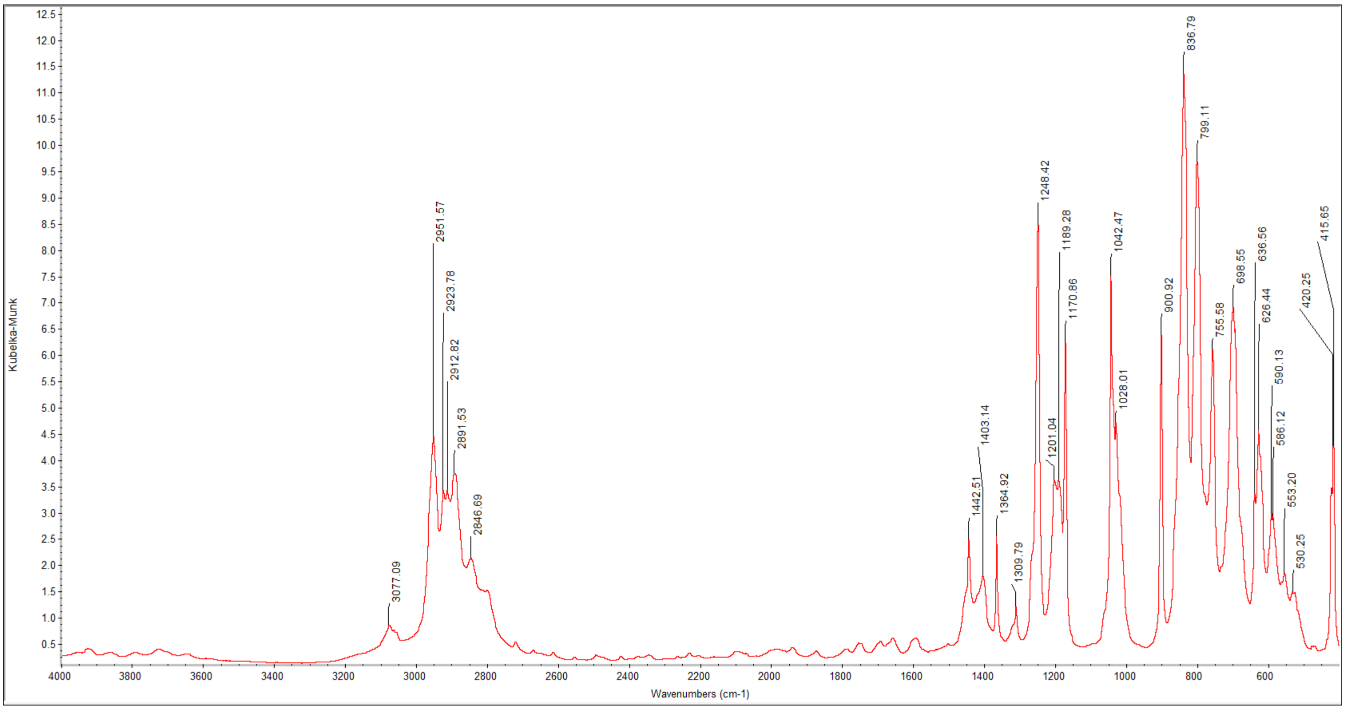


**Figure S36.** DRIFT spectrum of [Cp'$_6$La$_6$I$_8$(AlMe$_4$)$_4$] (**4a**).

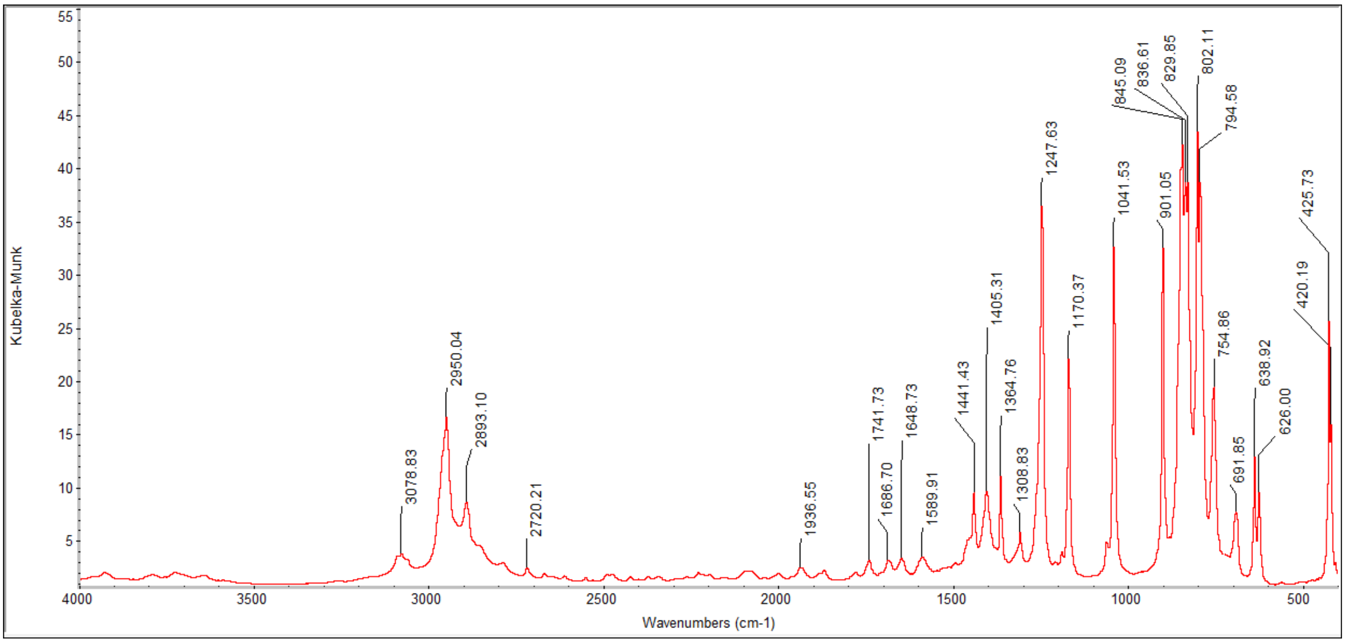


**Figure S37.** IR-DRIFT spectrum of [Cp'LaI$_2$]$_{12}$ (**5a**).

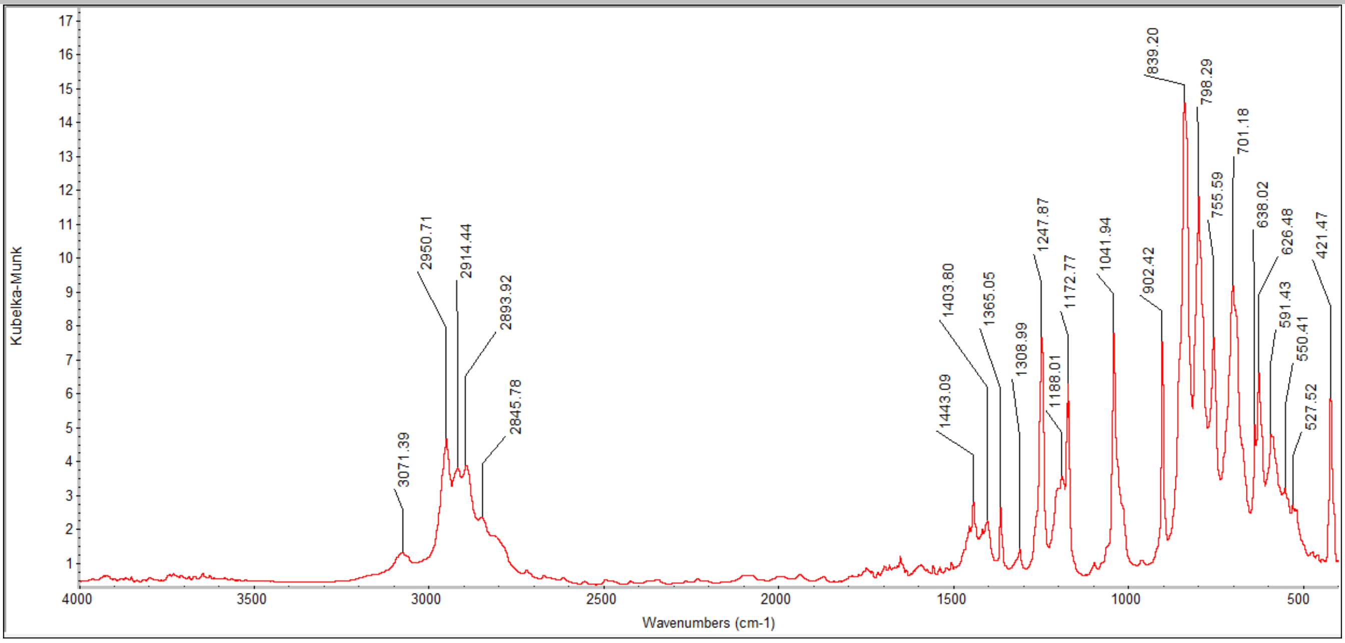


**Figure S38.** IR-DRIFT spectrum of [Cp'$_6$La$_6$Br$_8$(AlMe$_4$)$_4$] (**4b**).

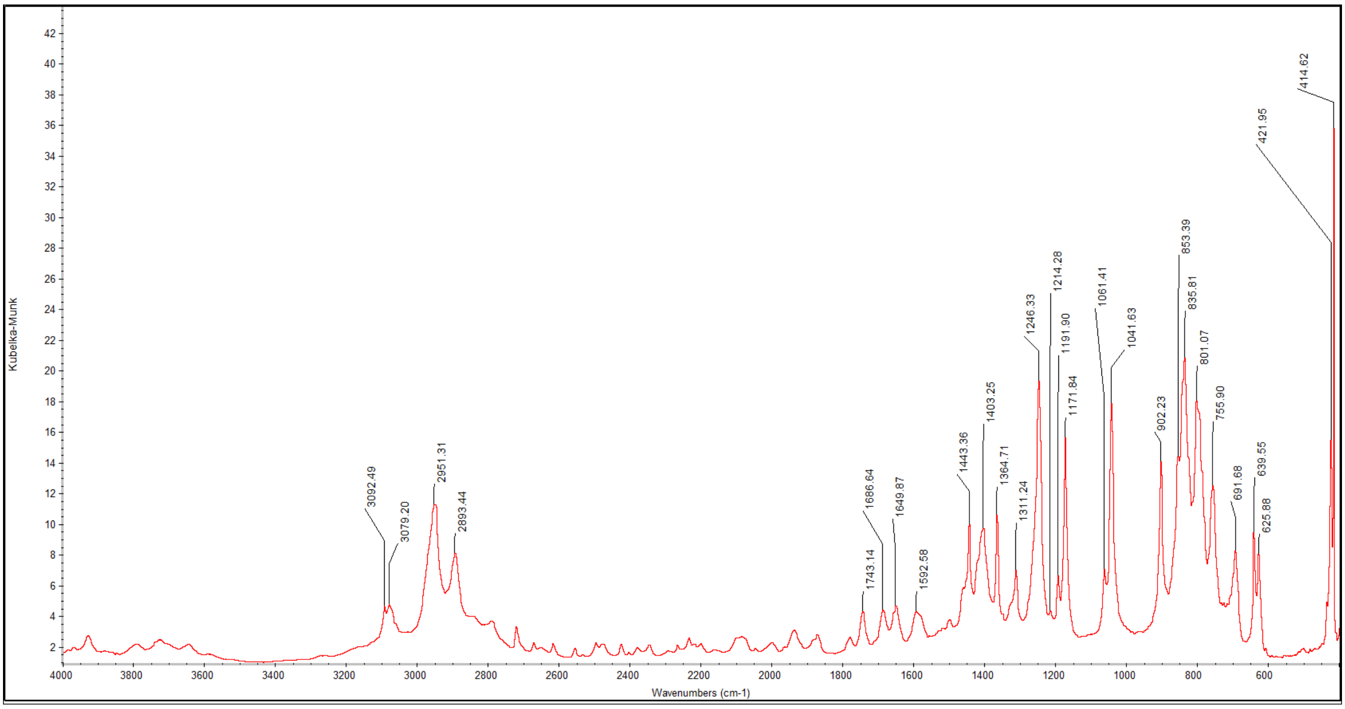


**Figure S39.** DRIFT spectrum of [Cp'LaBr$_2$]$_{12}$ (**5b**).

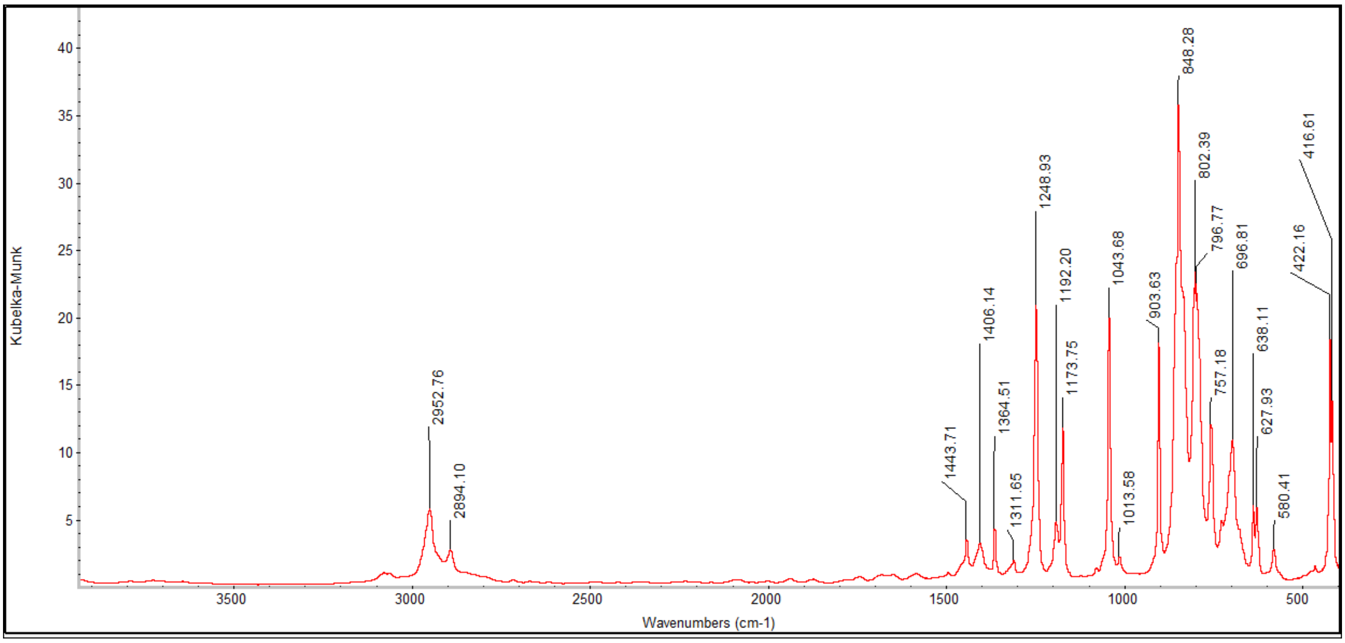


**Figure S40.** DRIFT spectrum of [Cp'LaCl$_2$]$_{10}$ (**8**).

# EDX Measurements

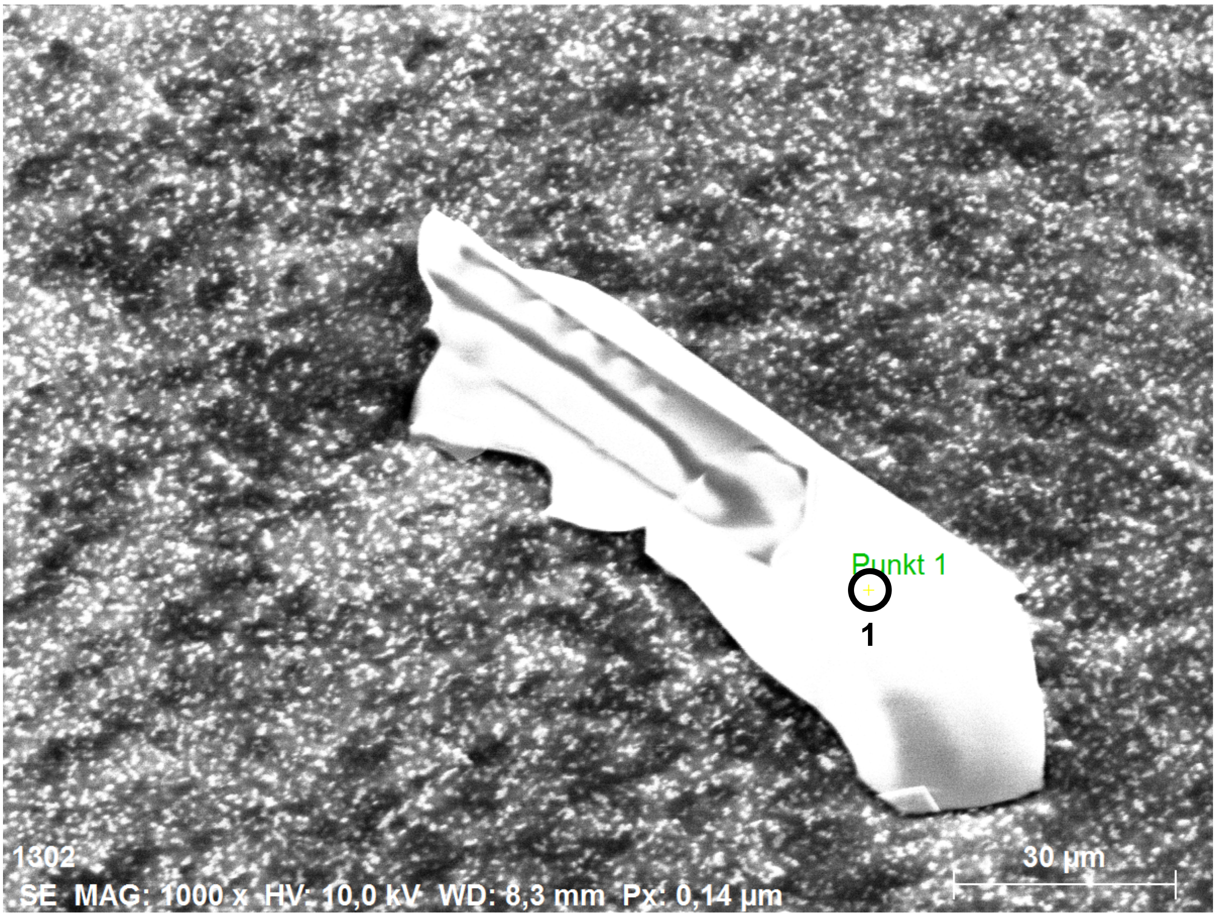


**Figure S41.** SEM image of crystalline [Cp'$_6$La$_6$I$_8$(AlMe$_4$)$_4$] (**4a**) in position 1, where EDX measurement was performed.

**Table S3.** Results of EDX measurement at position 1 (Fig. S41)

| Element | norm. wt. % | norm. atom % | 3 σ | norm. wt. % calc. | norm. atom % calc. |
|---|---|---|---|---|---|
| Al | 5.47 | 17.77 | 0.43 | 5.09 | 16.67 |
| Si | 8.01 | 24.99 | 0.56 | 7.93 | 25.00 |
| La | 40.75 | 25.68 | 2.16 | 39.22 | 25.00 |
| I | 45.75 | 31.56 | 2.29 | 47.77 | 33.33 |

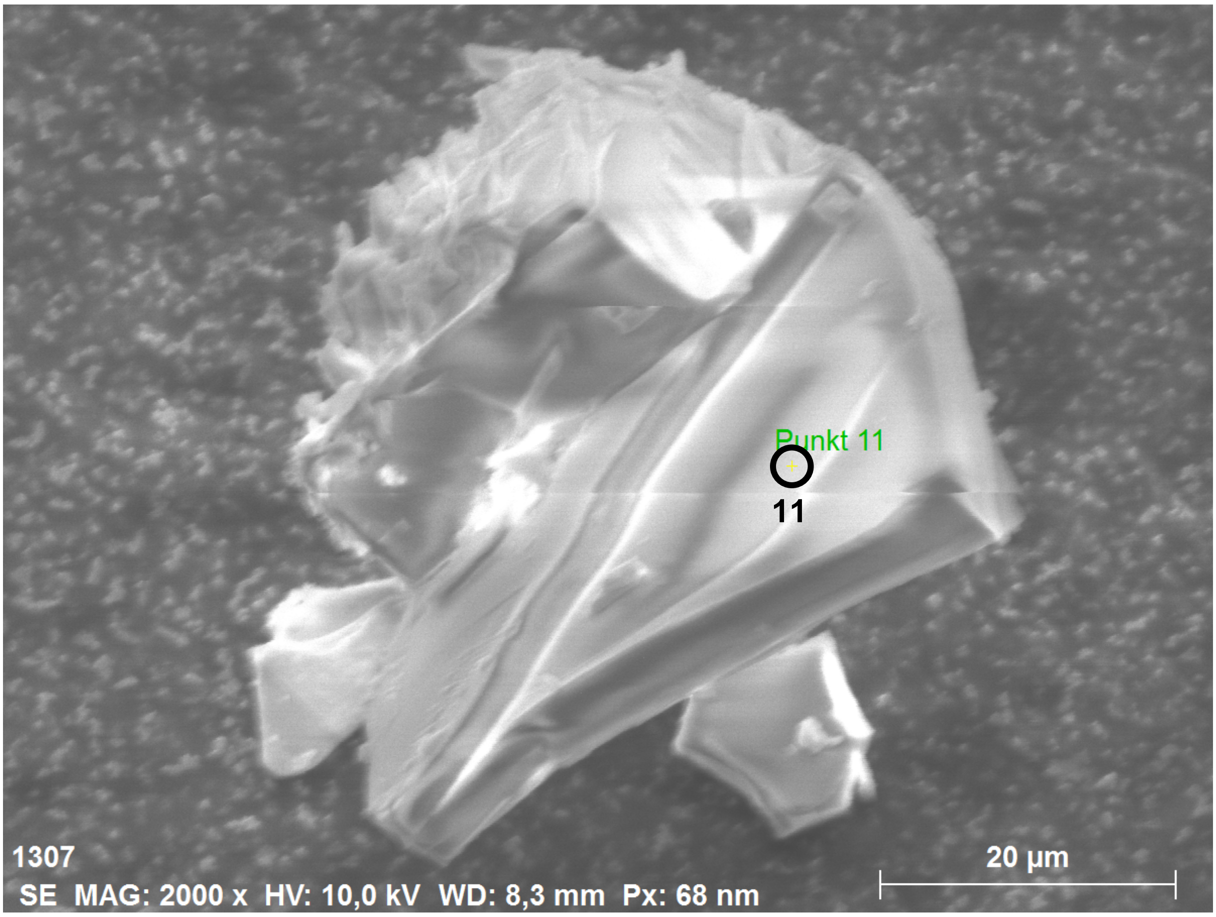


**Figure S42.** SEM image of crystalline [$Cp'_6La_6I_8(AlMe_4)_4$] (**4a**) in position 11, where EDX measurement was performed.

**Table S4.** Results of EDX measurement at position 11 (Fig. S42)

| Element | norm. wt. % | norm. atom % | 3 σ | norm. wt. % calc. | norm. atom % calc. |
|---|---|---|---|---|---|
| Al | 5.28 | 17.45 | 0.41 | 5.09 | 16.67 |
| Si | 7.45 | 23.65 | 0.52 | 7.93 | 25.00 |
| La | 39.86 | 25.59 | 2.07 | 39.22 | 25.00 |
| I | 47.39 | 33.30 | 2.31 | 47.77 | 33.33 |

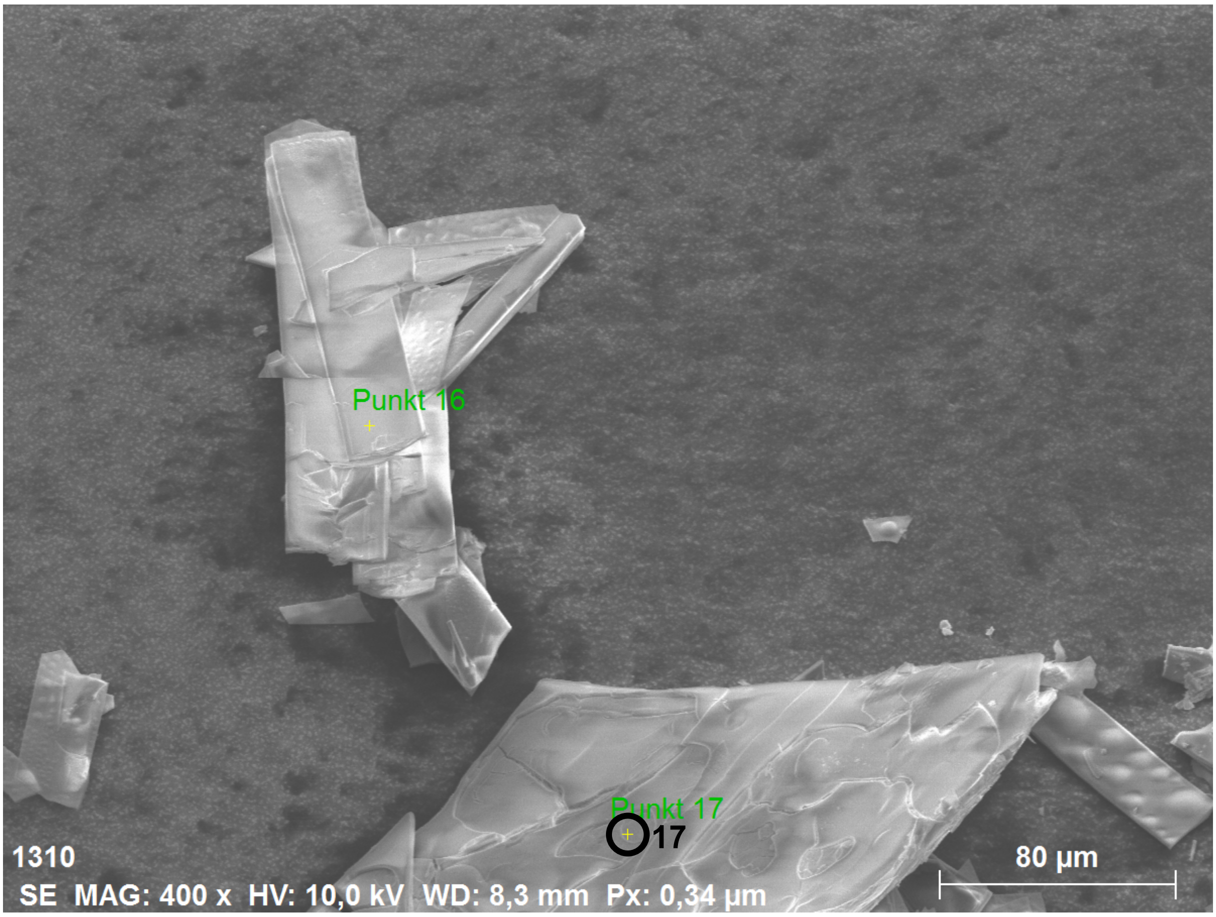


**Figure S43.** SEM image of crystalline [Cp'$_6$La$_6$I$_8$(AlMe$_4$)$_4$] (**4a**) in position 17, where EDX measurement was performed.

**Table S5.** Results of EDX measurement at position 17 (Fig. S43)

| Element | norm. wt. % | norm. atom % | 3 σ | norm. wt. % calc. | norm. atom % calc. |
|---|---|---|---|---|---|
| Al | 5.33 | 17.38 | 0.43 | 5.09 | 16.67 |
| Si | 7.98 | 24.99 | 0.57 | 7.93 | 25.00 |
| La | 40.74 | 25.79 | 2.21 | 39.22 | 25.00 |
| I | 45.95 | 31.84 | 2.35 | 47.77 | 33.33 |

**Table S6.** Averaged results of all EDX measurements for **2a** (6 measurements)

| Element | norm. wt. % | norm. atom % | 3 σ | norm. wt. % calc. | norm. atom % calc. |
|---|---|---|---|---|---|
| Al | 5.23 | 17.17 | 0.41 | 5.09 | 16.67 |
| Si | 7.77 | 24.52 | 0.54 | 7.93 | 25.00 |
| La | 40.77 | 26.02 | 2.16 | 39.22 | 25.00 |
| I | 46.24 | 32.30 | 2.31 | 47.77 | 33.33 |

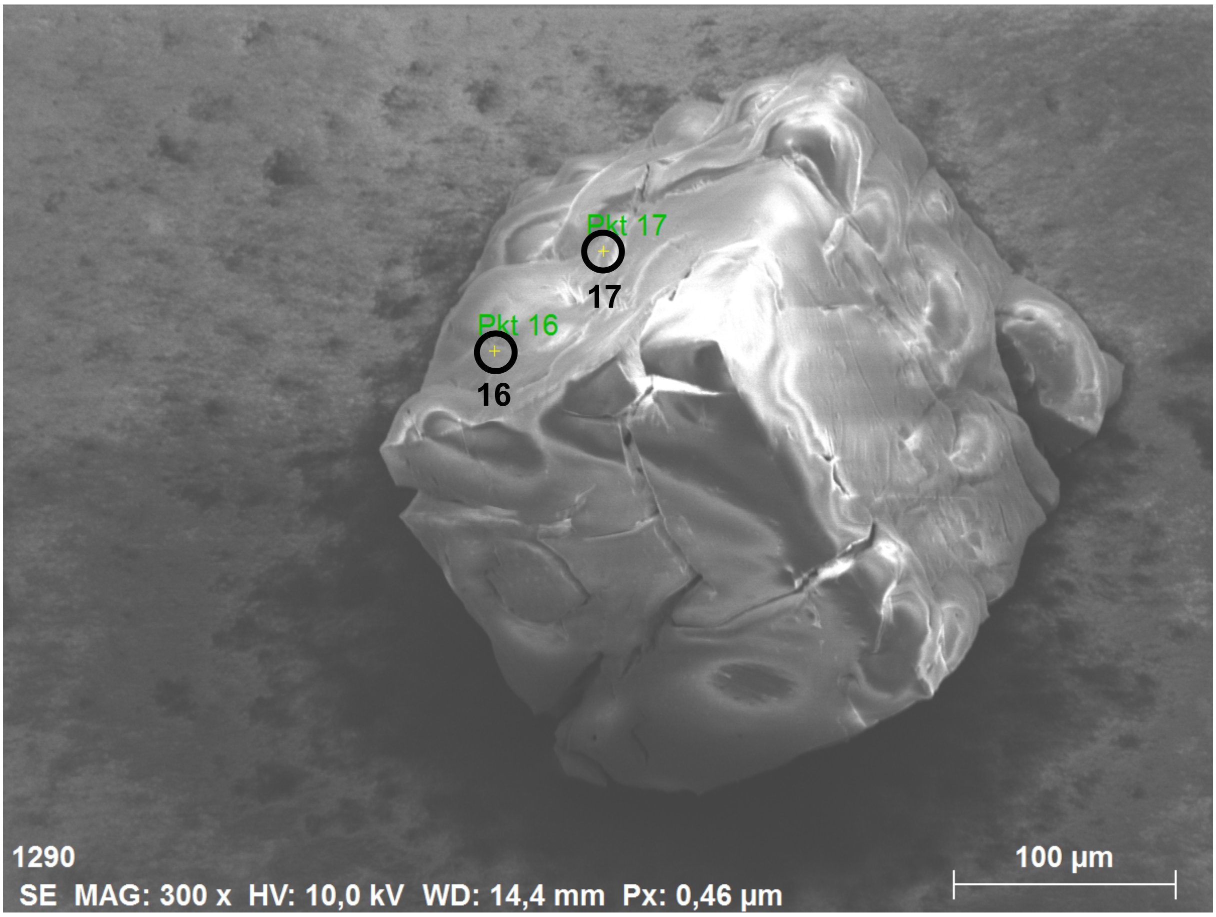


**Figure S44.** SEM image of crystalline $[Cp'LaI_2]_{12}$ (**5a**) in positions 16 and 17, where EDX measurements were performed.

**Table S7.** Results of EDX measurement at position 16 (Fig. S44)

| Element | norm. wt. % | norm. atom % | 3 σ | norm. wt. % calc. | norm. atom % calc. |
|---|---|---|---|---|---|
| Si | 6.54 | 24.62 | 0.46 | 6.67 | 25.00 |
| La | 33.39 | 25.39 | 2.72 | 33.01 | 25.00 |
| I | 60.06 | 49.99 | 3.28 | 60.31 | 50.00 |

**Table S8.** Results of EDX measurement at position 17 (Fig. S44)

| Element | norm. wt. % | norm. atom % | 3 σ | norm. wt. % calc. | norm. atom % calc. |
|---|---|---|---|---|---|
| Si | 6.59 | 24.74 | 0.48 | 6.67 | 25.00 |
| La | 32.59 | 24.73 | 2.68 | 33.01 | 25.00 |
| I | 60.82 | 50.52 | 3.12 | 60.31 | 50.00 |

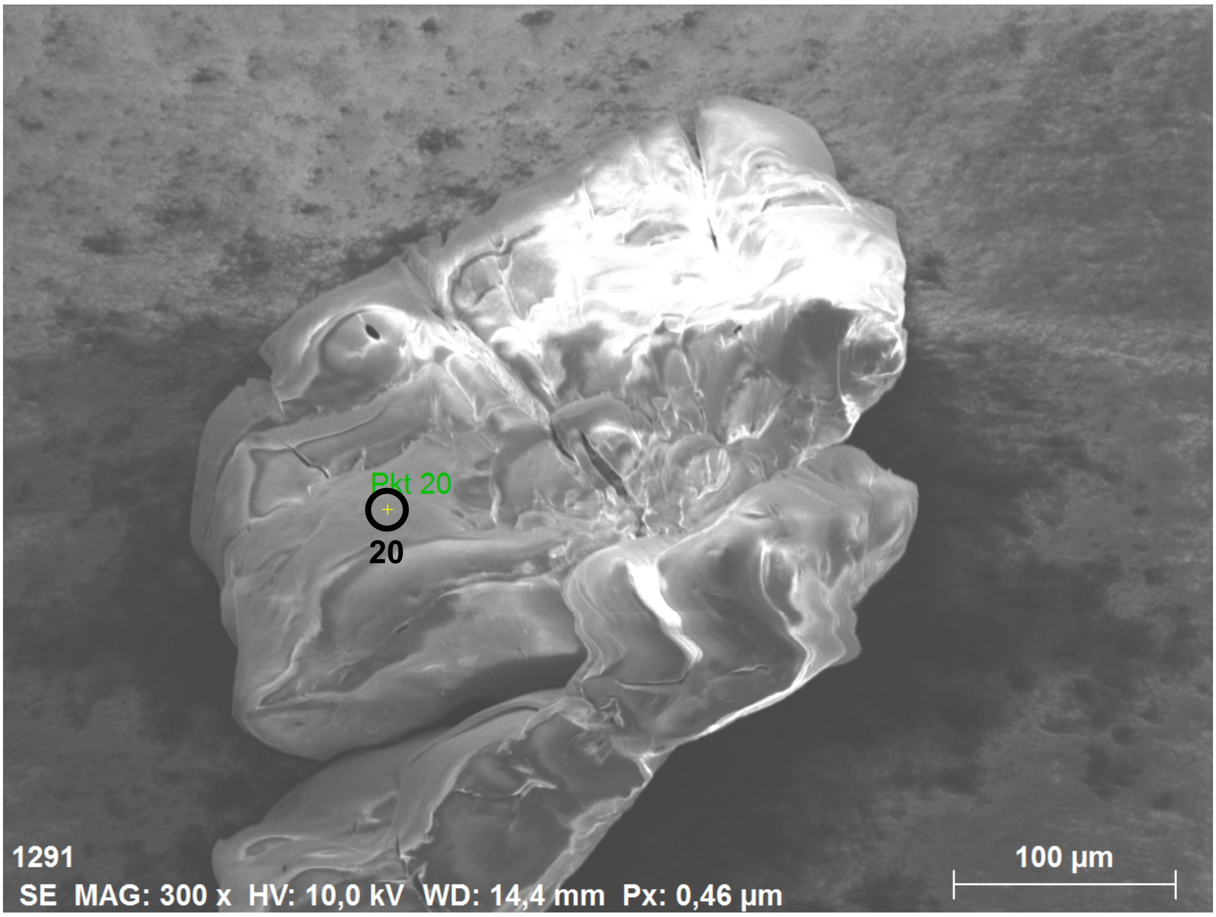


**Figure S45.** SEM image of crystalline [Cp'La$I_2$]$_{12}$ (**5a**) in position 20, where EDX measurement was performed.

**Table S9.** Results of EDX measurement at position 20 (Fig. S45)

| Element | norm. wt. % | norm. atom % | 3 σ | norm. wt. % calc. | norm. atom % calc. |
|---|---|---|---|---|---|
| Si | 6.74 | 25.19 | 0.45 | 6.67 | 25.00 |
| La | 32.51 | 24.57 | 3.61 | 33.01 | 25.00 |
| I | 60.74 | 50.24 | 4.04 | 60.31 | 50.00 |

**Table S10.** Averaged results of all EDX measurements for **2b** (12 measurements)

| Element | norm. wt. % | norm. atom % | 3 σ | norm. wt. % calc. | norm. atom % calc. |
|---|---|---|---|---|---|
| Si | 6.41 | 24.19 | 0.41 | 6.67 | 25.00 |
| La | 33.27 | 25.41 | 2.65 | 33.01 | 25.00 |
| I | 60.32 | 50.40 | 2.98 | 60.31 | 50.00 |

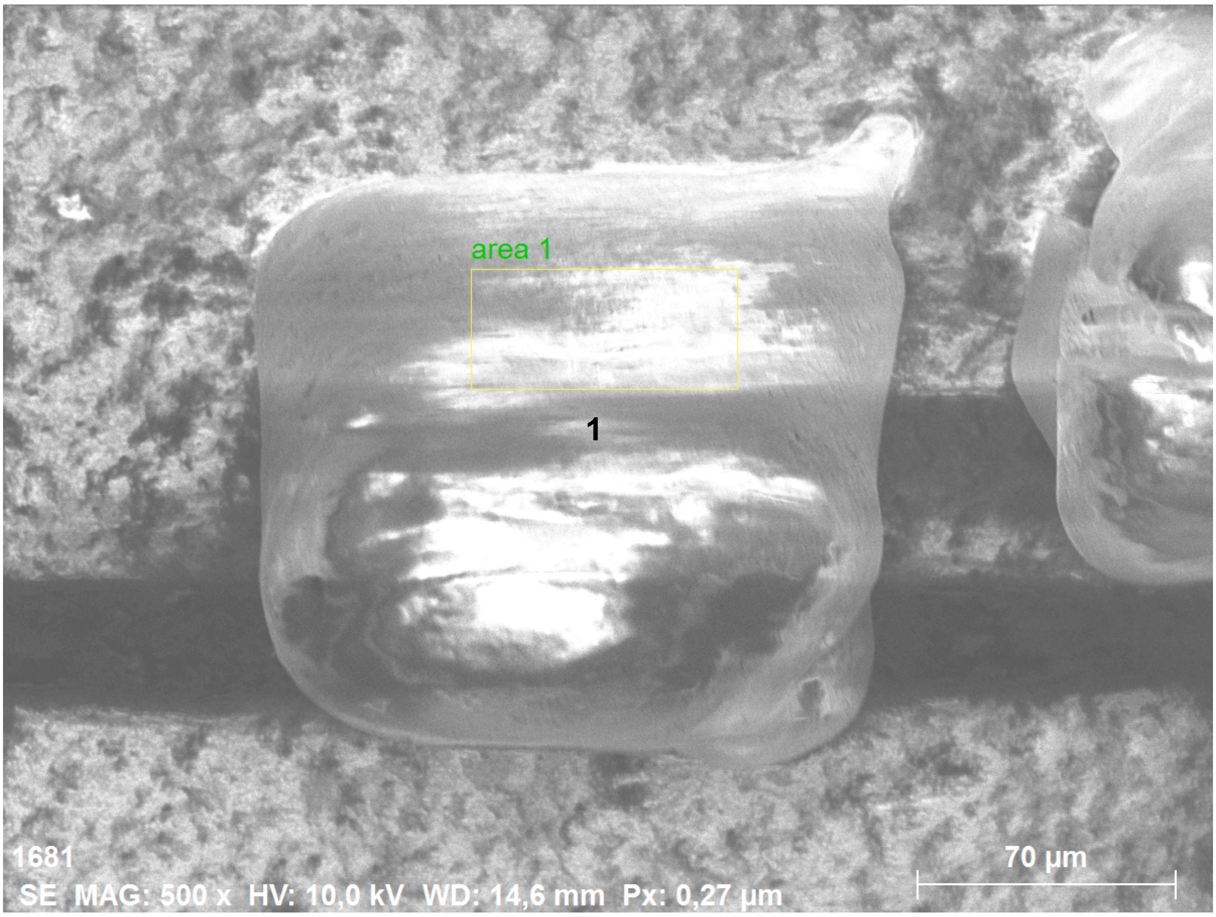


**Figure S46.** SEM image of crystalline [($\mu$-Cp)$_2$Cp'$_8$La$_8$I$_{14}$] (**6**) of area 1, where EDX measurement was performed.

**Table S11.** Results of EDX measurement of area 1 (Fig. S46)

| Element | norm. wt. % | norm. atom % | 3 σ | norm. wt. % calc. | norm. atom % calc. |
|---|---|---|---|---|---|
| Si | 7.05 | 26.17 | 0.45 | 7.22 | 26.67 |
| La | 35.67 | 26.77 | 1.48 | 35.70 | 26.67 |
| I | 57.28 | 47.05 | 2.13 | 57.08 | 46.67 |

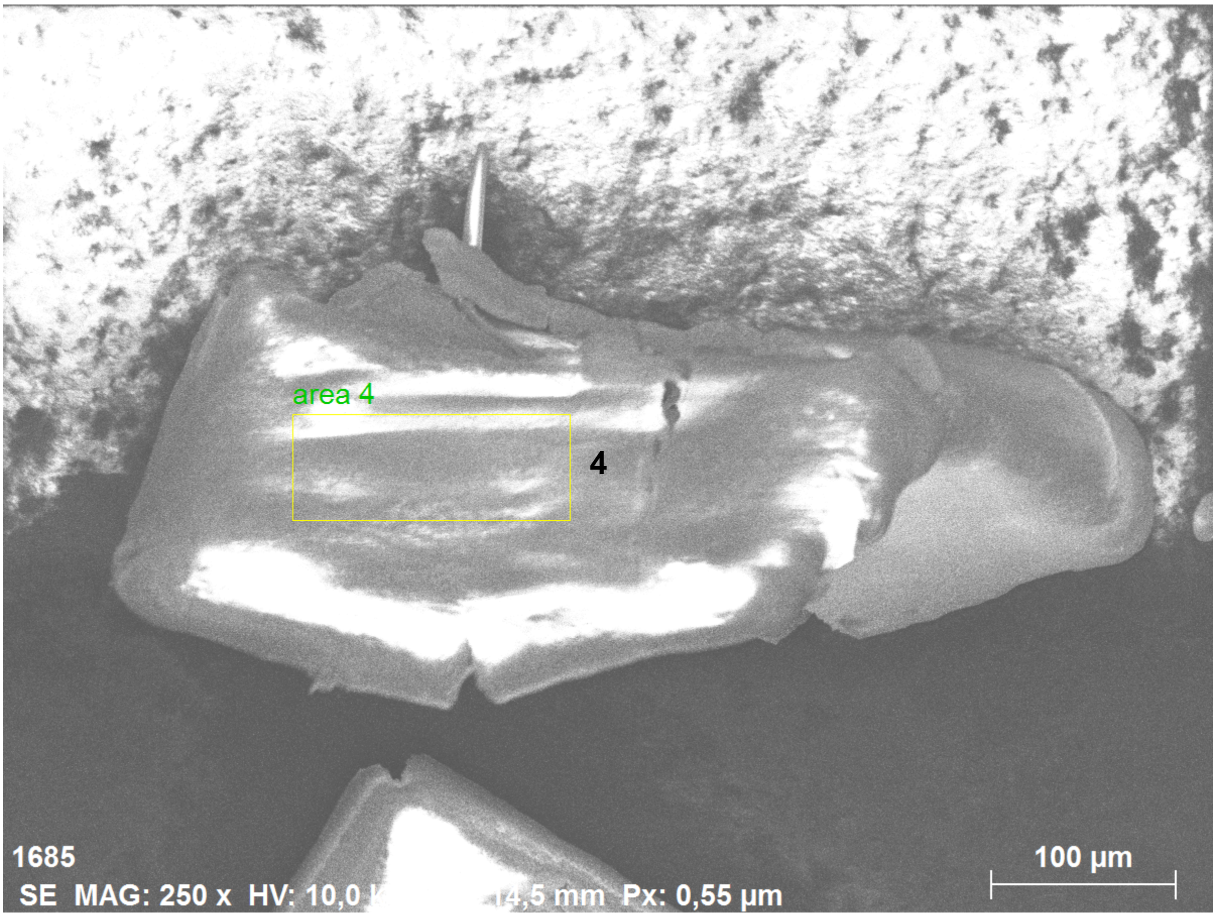


**Figure S47.** SEM image of crystalline [($\mu$-Cp)$_2$Cp'$_8$La$_8$I$_{14}$] (**6**) of area 4, where EDX measurement was performed.

**Table S12.** Results of EDX measurement of area 4 (Fig. S47)

| **Element** | **norm. wt. %** | **norm. atom %** | **3 σ** | **norm. wt. % calc.** | **norm. atom % calc.** |
|---|---|---|---|---|---|
| Si | 7.15 | 26.45 | 0.43 | 7.22 | 26.67 |
| La | 35.65 | 26.68 | 1.55 | 35.70 | 26.67 |
| I | 57.21 | 46.86 | 2.24 | 57.08 | 46.67 |

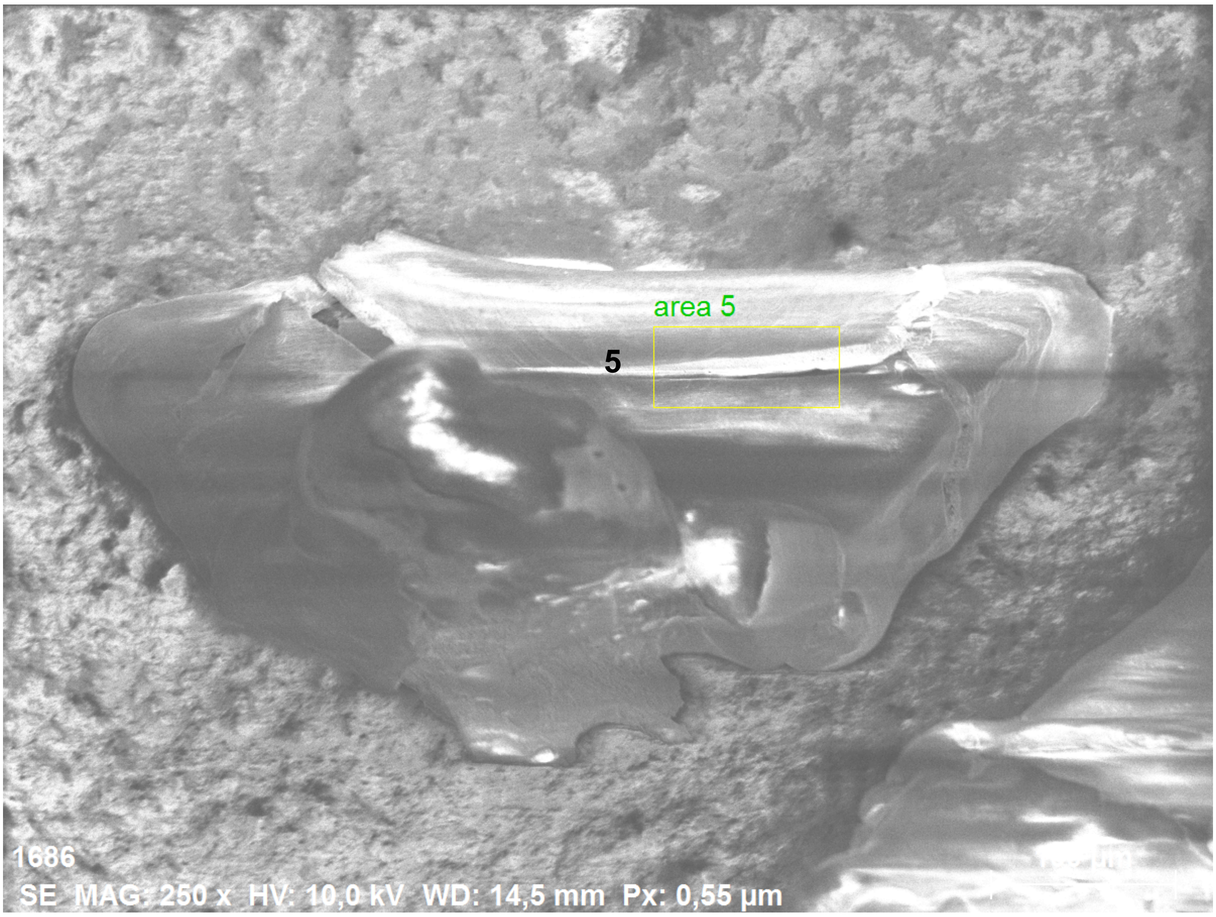


**Figure S48.** SEM image of crystalline [($\mu$-Cp)$_2$Cp'$_8$La$_8$I$_{14}$] (**6**) of area 5, where EDX measurement was performed.

**Table S13.** Results of EDX measurement of area 5 (Fig. S48).

| Element | norm. wt. % | norm. atom % | 3 σ | norm. wt. % calc. | norm. atom % calc. |
|---|---|---|---|---|---|
| Si | 7.11 | 26.34 | 0.46 | 7.22 | 26.67 |
| La | 35.54 | 26.63 | 1.60 | 35.70 | 26.67 |
| I | 57.35 | 47.03 | 2.32 | 57.08 | 46.67 |

**Table S14.** Averaged results of all EDX measurements for **2c** (5 measurements)

| Element | norm. wt. % | norm. atom % | 3 σ | norm. wt. % calc. | norm. atom % calc. |
|---|---|---|---|---|---|
| Si | 7.07 | 26.22 | 0.46 | 7.22 | 26.67 |
| La | 35.54 | 26.66 | 1.62 | 35.70 | 26.67 |
| I | 57.39 | 47.12 | 2.36 | 57.08 | 46.67 |

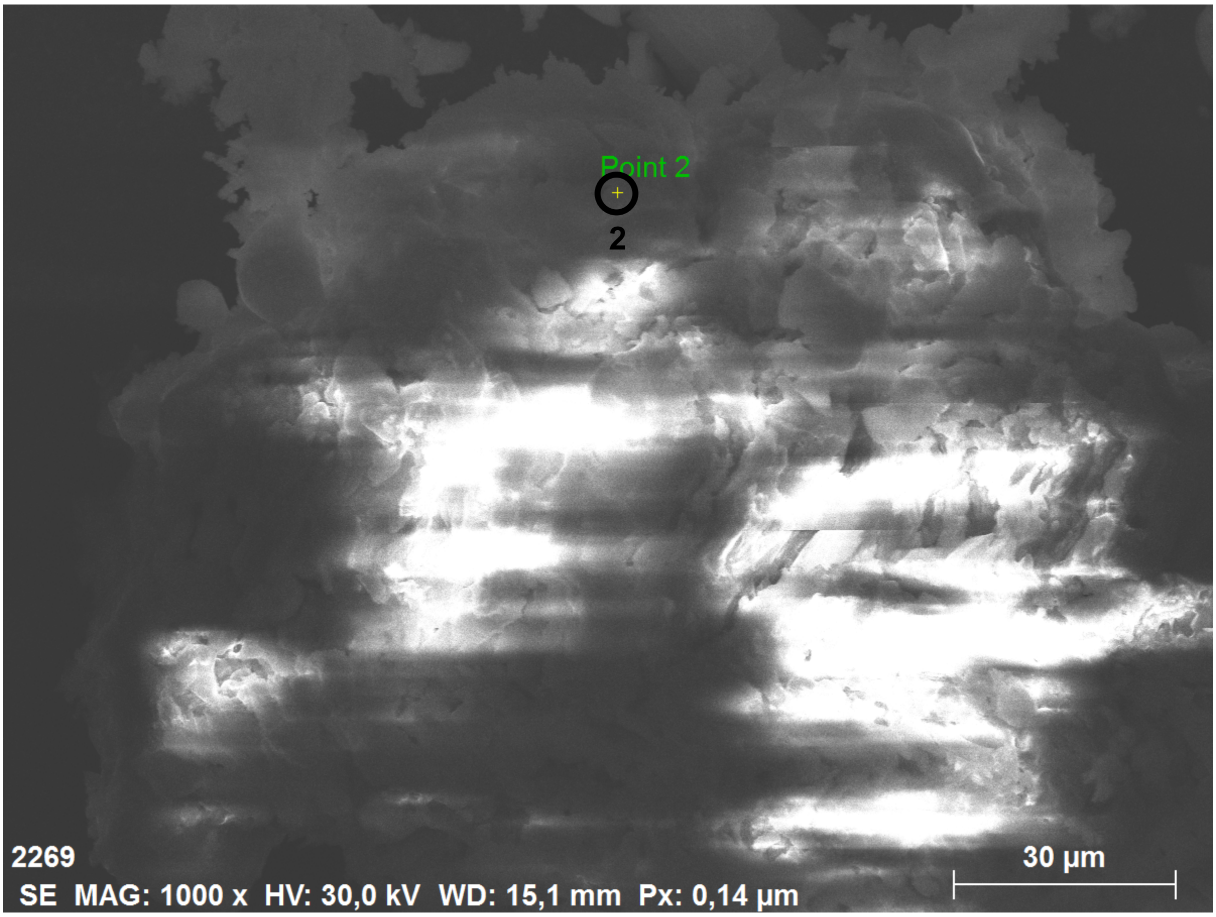


**Figure S49.** SEM image of crystalline [$Cp'_6La_6Br_8(AlMe_4)_4$] (**4b**) in position 2, where EDX measurement was performed.

**Table S15.** Results of EDX measurement at position 2 (Fig. S49)

| Element | norm. wt. % | norm. atom % | 3 σ | norm. wt. % calc. | norm. atom % calc. |
|---|---|---|---|---|---|
| Si | 8.40 | 25.58 | 1.04 | 9.63 | 25.00 |
| La | 54.29 | 33.42 | 3.74 | 47.65 | 25.00 |

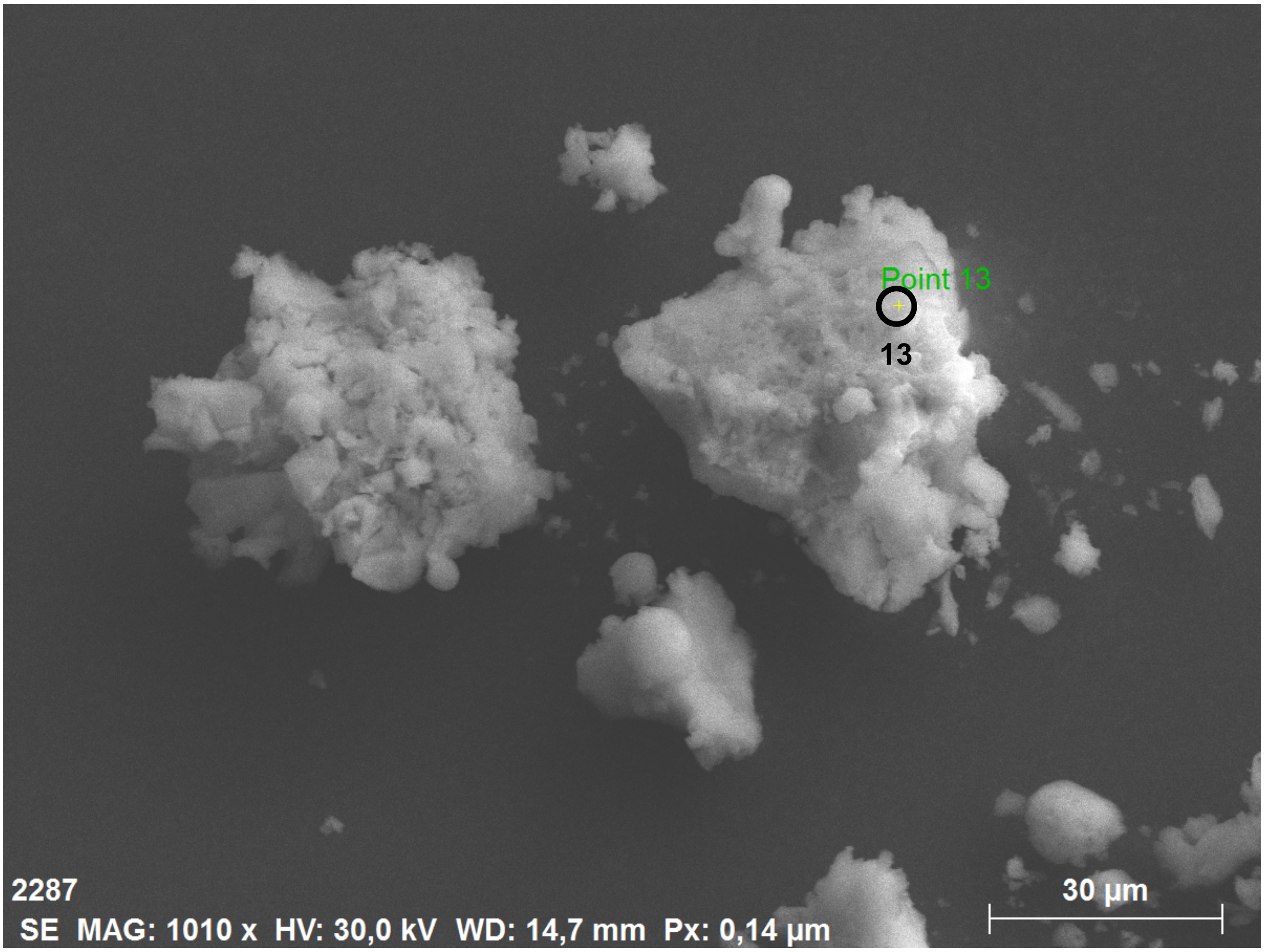


**Figure S50.** SEM image of crystalline [$Cp'_6La_6Br_8(AlMe_4)_4$] (**4b**) in position 13, where EDX measurement was performed.

**Table S16.** Results of EDX measurement at position 13 (Fig. S50)

| Element | norm. wt. % | norm. atom % | 3 σ | norm. wt. % calc. | norm. atom % calc. |
|---|---|---|---|---|---|
| Si | 7.89 | 24.03 | 1.04 | 9.63 | 25.00 |
| La | 49.85 | 30.71 | 3.74 | 47.65 | 25.00 |

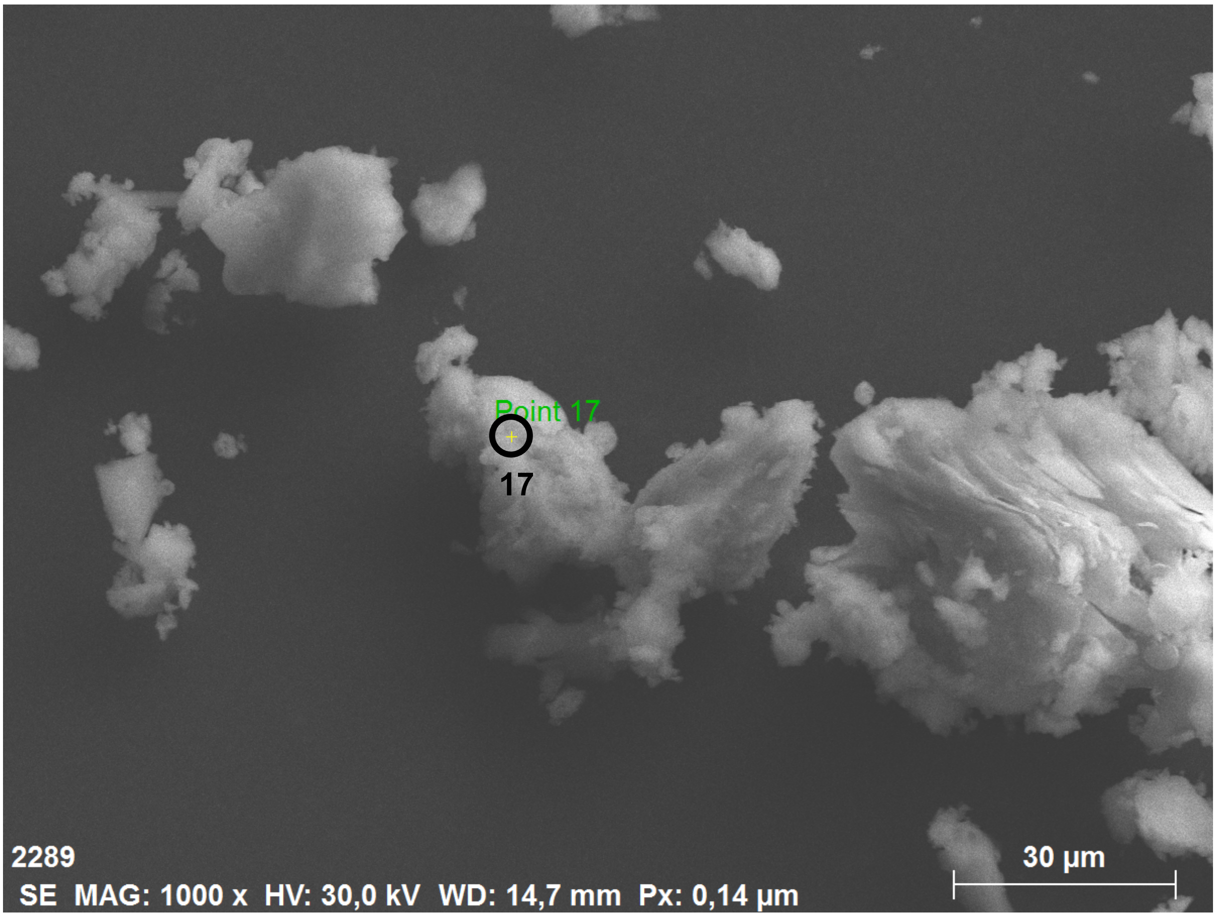


**Figure S51.** SEM image of crystalline [$Cp'_6La_6Br_8(AlMe_4)_4$] (**4b**) in position 17, where EDX measurement was performed.

**Table S17.** Results of EDX measurement at position 17 (Fig. S51)

| Element | norm. wt. % | norm. atom % | 3 σ | norm. wt. % calc. | norm. atom % calc. |
|---|---|---|---|---|---|
| Si | 7.56 | 22.86 | 1.04 | 9.63 | 25.00 |
| La | 46.70 | 30.71 | 3.56 | 47.65 | 25.00 |

**Table S18.** Averaged results of all EDX measurements for **3a** (18 measurements)

| Element | norm. wt. % | norm. atom % | 3 σ | norm. wt. % calc. | norm. atom % calc. |
|---|---|---|---|---|---|
| Si | 6.99 | 21.85 | 0.90 | 9.63 | 25.00 |
| La | 48.76 | 32.64 | 3.62 | 47.65 | 25.00 |

The ratios for aluminum and bromine could not be determined reliably due to overlap of the respective signals.[11]

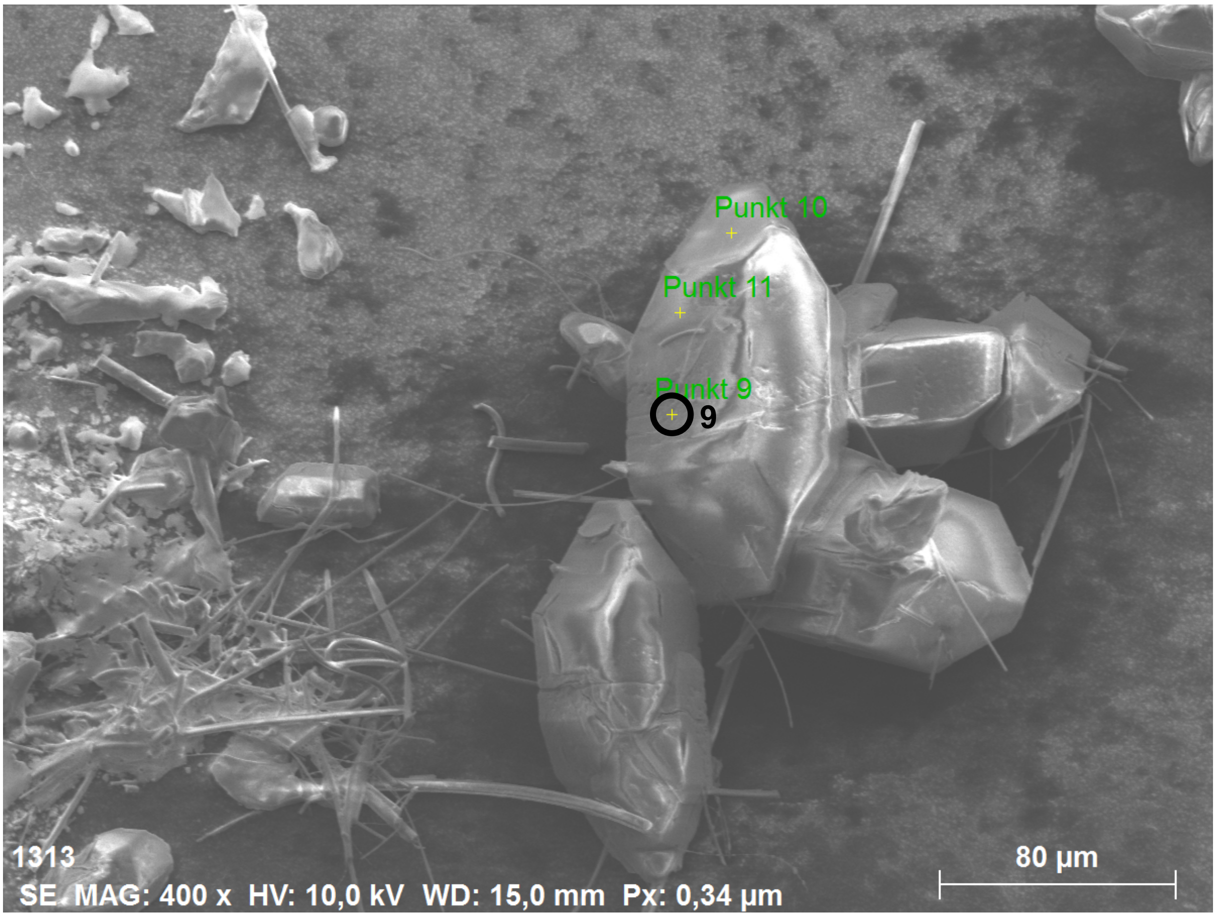


**Figure S52.** SEM image of crystalline [Cp'$LaBr_2$]$_{12}$ (**5b**) in position 9, where EDX measurement was performed.

**Table S19.** Results of EDX measurement at position 9 (Fig. S52)

| Element | norm. wt. % | norm. atom % | 3 σ | norm. wt. % calc. | norm. atom % calc. |
|---|---|---|---|---|---|
| Si | 8.75 | 25.46 | 0.61 | 8.59 | 25.00 |
| La | 43.19 | 25.41 | 2.38 | 42.50 | 25.00 |
| Br | 48.05 | 49.14 | 3.16 | 48.90 | 50.00 |

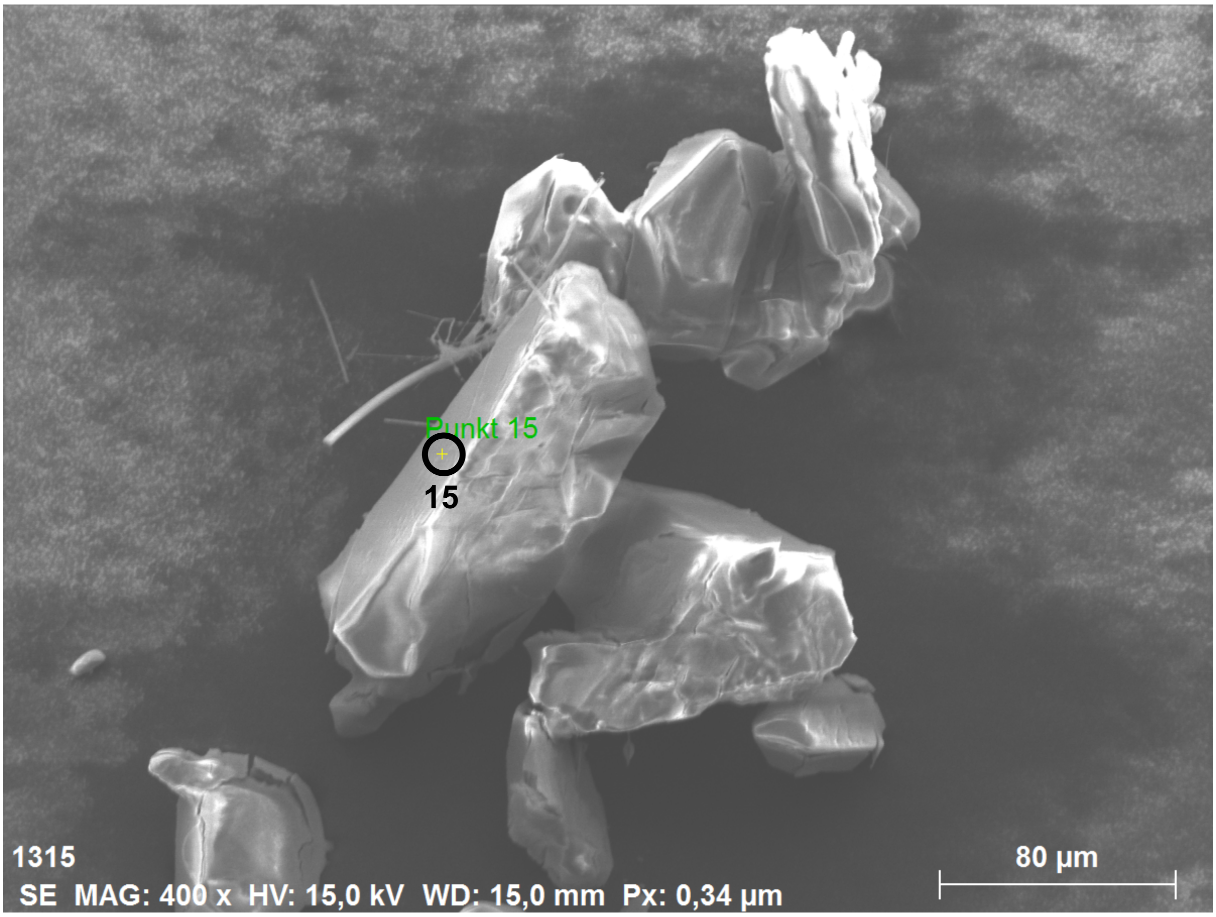


**Figure S53.** SEM image of crystalline [Cp'LaBr$_2$]$_{12}$ (**5b**) in position 15, where EDX measurement was performed.

**Table S20.** Results of EDX measurement at position 15 (Fig. S53)

| Element | norm. wt. % | norm. atom % | 3 σ | norm. wt. % calc. | norm. atom % calc. |
|---|---|---|---|---|---|
| Si | 8.24 | 24.07 | 0.73 | 8.59 | 25.00 |
| La | 41.91 | 24.75 | 2.36 | 42.50 | 25.00 |
| Br | 49.85 | 51.18 | 4.45 | 48.90 | 50.00 |

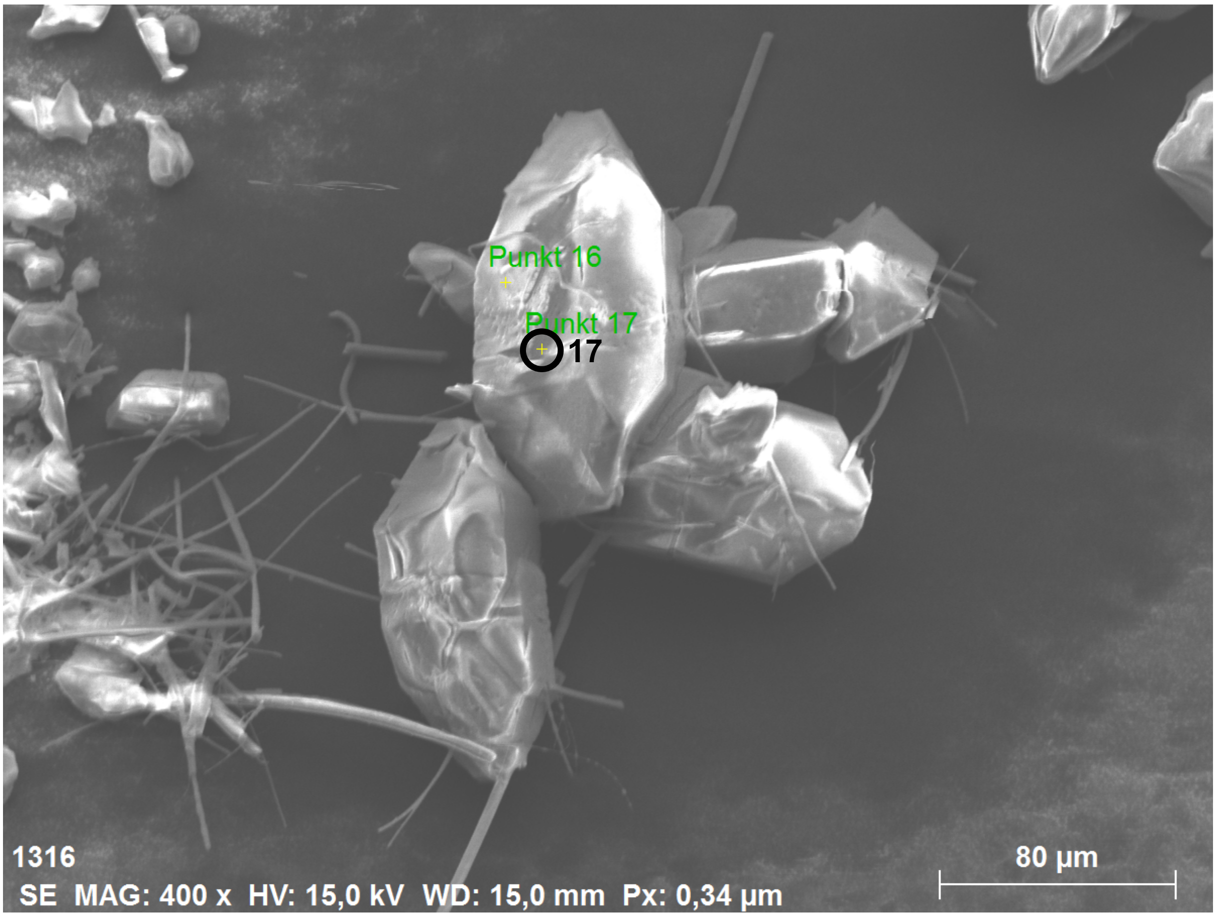


**Figure S54.** SEM image of crystalline [Cp'LaBr$_2$]$_{12}$ (**5b**) in position 17, where EDX measurement was performed.

**Table S21.** Results of EDX measurement at position 17 (Fig. S54)

| Element | norm. wt. % | norm. atom % | 3 σ | norm. wt. % calc. | norm. atom % calc. |
|---|---|---|---|---|---|
| Si | 8.24 | 24.07 | 0.58 | 8.59 | 25.00 |
| La | 41.91 | 24.75 | 1.85 | 42.50 | 25.00 |
| Br | 49.85 | 51.18 | 3.35 | 48.90 | 50.00 |

**Table S22.** Averaged results of all EDX measurements for **3b** (11 measurements)

| Element | norm. wt. % | norm. atom % | 3 σ | norm. wt. % calc. | norm. atom % calc. |
|---|---|---|---|---|---|
| Si | 8.27 | 24.24 | 0.57 | 8.59 | 25.00 |
| La | 42.78 | 25.35 | 2.13 | 42.50 | 25.00 |
| Br | 49.35 | 50.41 | 3.16 | 48.90 | 50.00 |

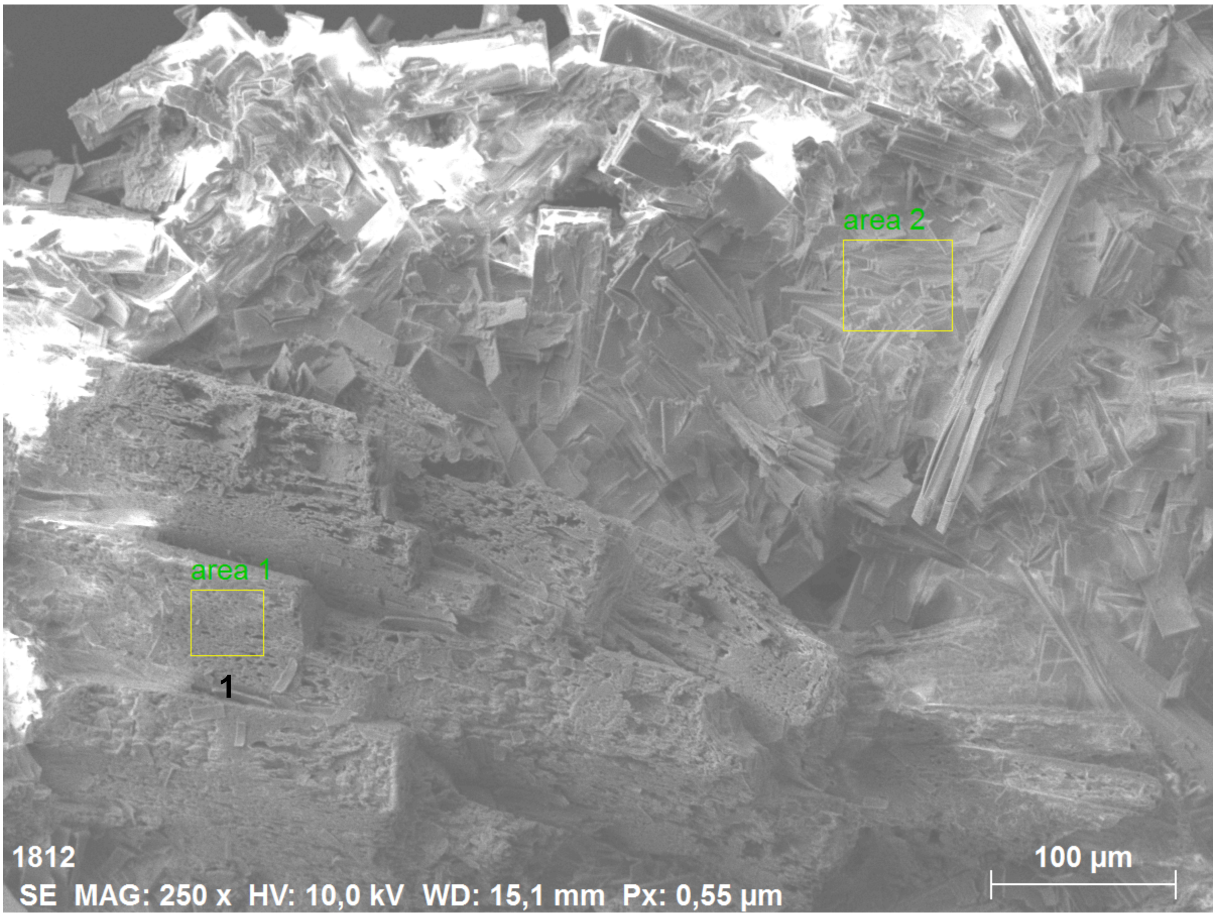


**Figure S55.** SEM image of crystalline [$Cp'_{10}La_{10}Br_{22}(AlMe_2)_2$] (**7**) of area 1, where EDX measurement was performed.

**Table S23.** Results of EDX measurement of area 1 (Fig. S55)

| Element | norm. wt. % | norm. atom % | 3 σ | norm. wt. % calc. | norm. atom % calc. |
|---|---|---|---|---|---|
| Si | 7.59 | 22.23 | 0.74 | 8.07 | 22.73 |
| La | 39.64 | 23.47 | 3.17 | 39.90 | 22.73 |

The values for area 2 could not be determined due to shadowing effects caused by the morphology of the crystal.

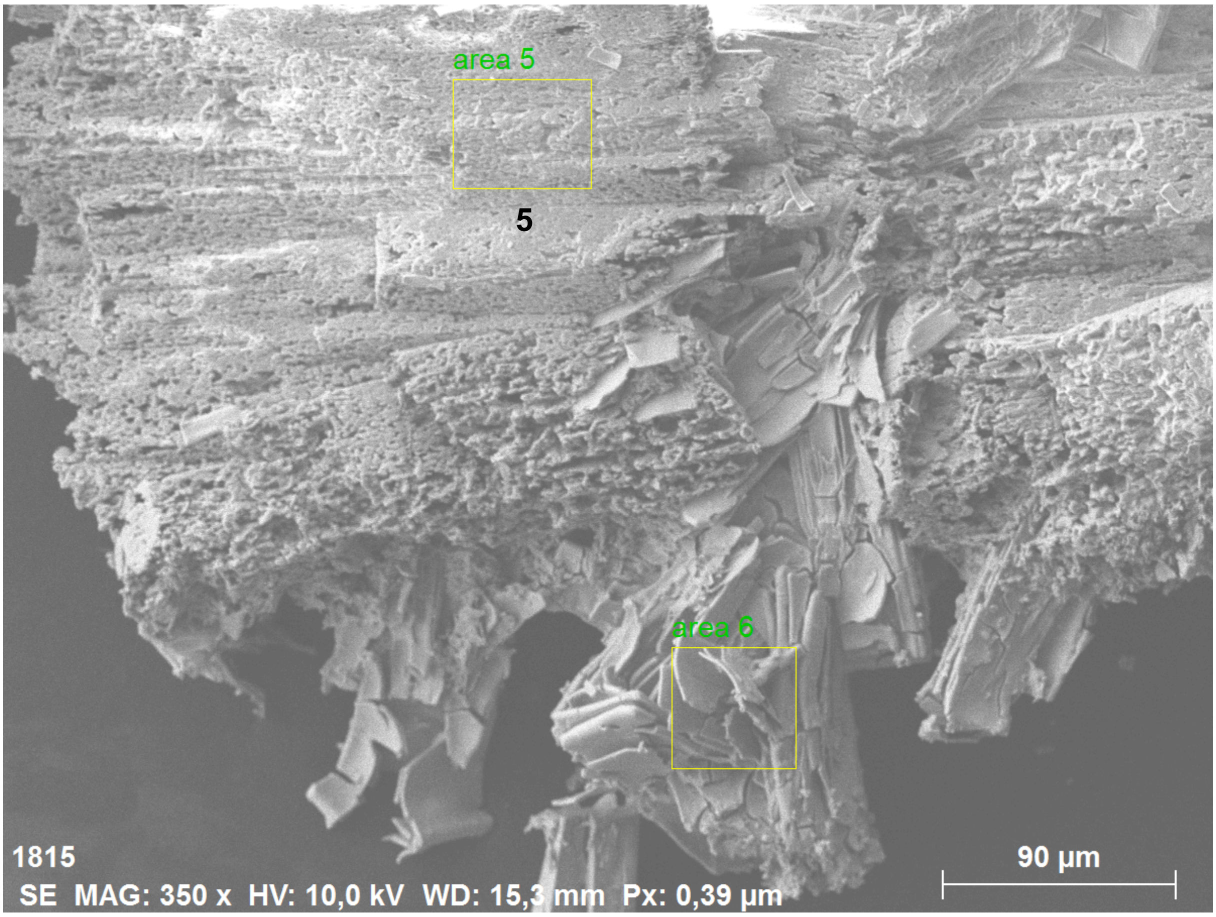


**Figure S56.** SEM image of crystalline [$Cp'_{10}La_{10}Br_{22}(AlMe_2)_2$] (**7**) of area 5, where EDX measurement was performed.

**Table S24.** Results of EDX measurement of area 5 (Fig. S56)

| Element | norm. wt. % | norm. atom % | 3 σ | norm. wt. % calc. | norm. atom % calc. |
|---|---|---|---|---|---|
| Si | 7.76 | 22.62 | 0.75 | 8.07 | 22.73 |
| La | 39.40 | 23.23 | 3.13 | 39.90 | 22.73 |

The values for area 6 could not be determined due to shadowing effects caused by the morphology of the crystal.

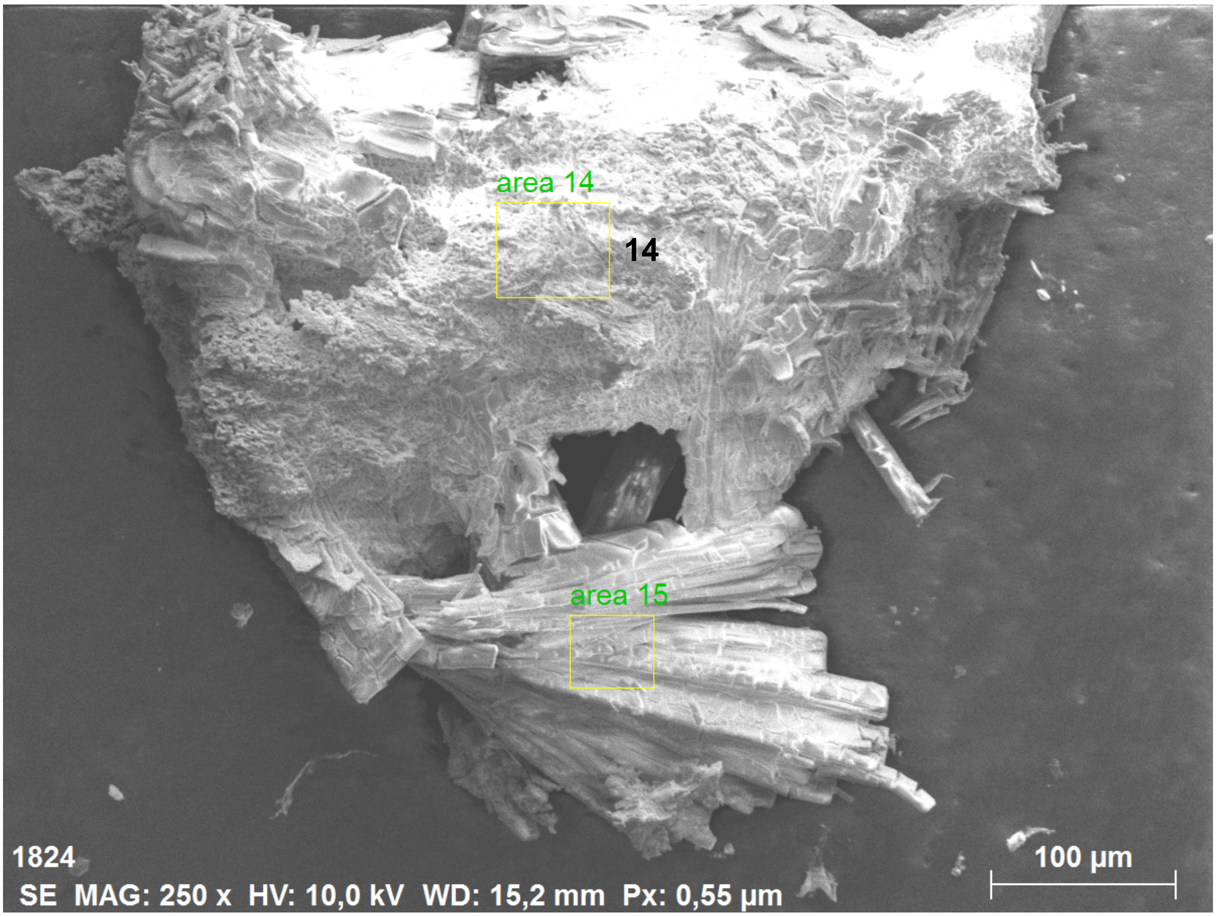


**Figure S57.** SEM image of crystalline [$Cp'_{10}La_{10}Br_{22}(AlMe_2)_2$] (**7**) of area 14, where EDX measurement was performed.

**Table S25.** Results of EDX measurement of area 14 (Fig. S57)

| Element | norm. wt. % | norm. atom % | 3 σ | norm. wt. % calc. | norm. atom % calc. |
|---|---|---|---|---|---|
| Si | 8.09 | 23.39 | 0.89 | 8.07 | 22.73 |
| La | 38.96 | 22.78 | 3.49 | 39.90 | 22.73 |

The values for area 15 could not be determined due to shadowing effects caused by the morphology of the crystal.

**Table S26.** Averaged results of all EDX measurements for **3c** (5 measurements)

| Element | norm. wt. % | norm. atom % | 3 σ | norm. wt. % calc. | norm. atom % calc. |
|---|---|---|---|---|---|
| Si | 7.64 | 22.26 | 0.83 | 8.07 | 22.73 |
| La | 38.84 | 22.89 | 3.40 | 39.90 | 22.73 |

The ratios for aluminum and bromine could not be determined reliably due to overlap of the respective signals.[11]

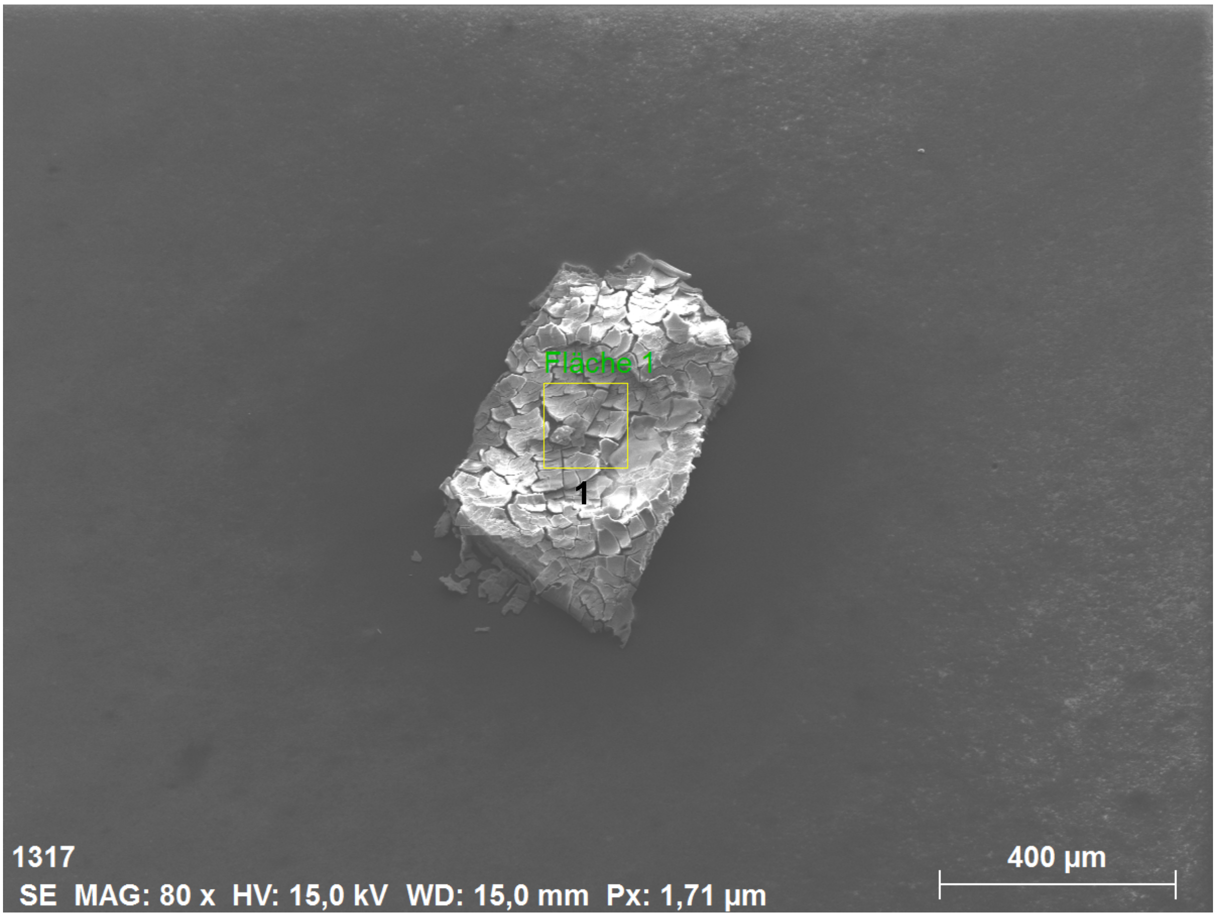


**Figure S58.** SEM image of crystalline [Cp'$La_2Cl_2$]$_{10}$ (**8**) of area 1, where EDX measurement was performed.

**Table S27.** Results of EDX measurement of area 1 (Fig. S58)

| Element | norm. wt. % | norm. atom % | 3 σ | norm. wt. % calc. | norm. atom % calc. |
|---|---|---|---|---|---|
| Si | 11.33 | 24.06 | 0.72 | 11.81 | 25.00 |
| La | 58.47 | 25.11 | 2.34 | 58.39 | 25.00 |
| Cl | 30.20 | 50.82 | 1.43 | 29.80 | 50.00 |

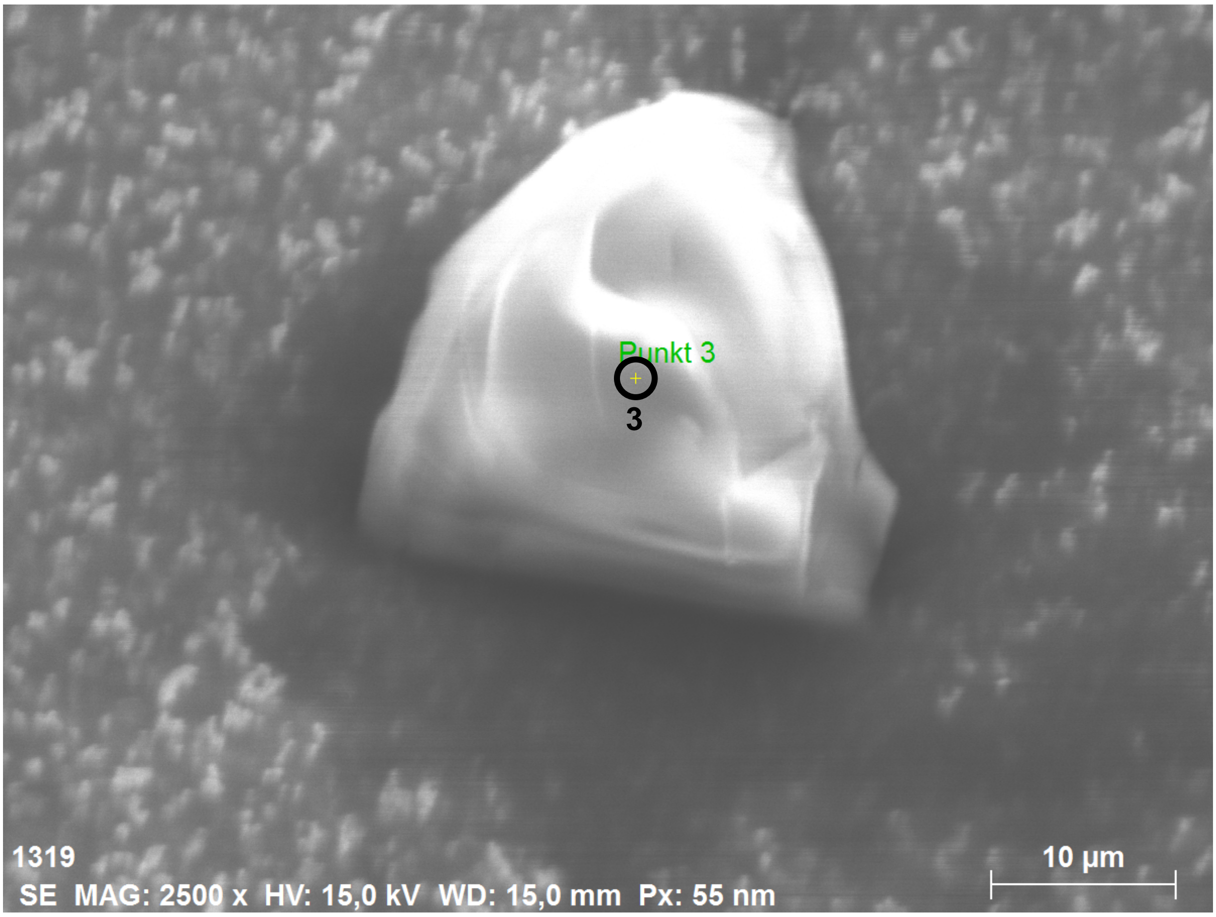


**Figure S59.** SEM image of crystalline $[Cp'La_2Cl_2]_{10}$ (**8**) in position 3, where EDX measurement was performed.

**Table S28.** Results of EDX measurement at position 3 (Fig. S59)

| Element | norm. wt. % | norm. atom % | 3 σ | norm. wt. % calc. | norm. atom % calc. |
|---|---|---|---|---|---|
| Si | 11.60 | 24.37 | 1.10 | 11.81 | 25.00 |
| La | 57.68 | 24.50 | 3.61 | 58.39 | 25.00 |
| Cl | 30.72 | 51.13 | 2.25 | 29.80 | 50.00 |

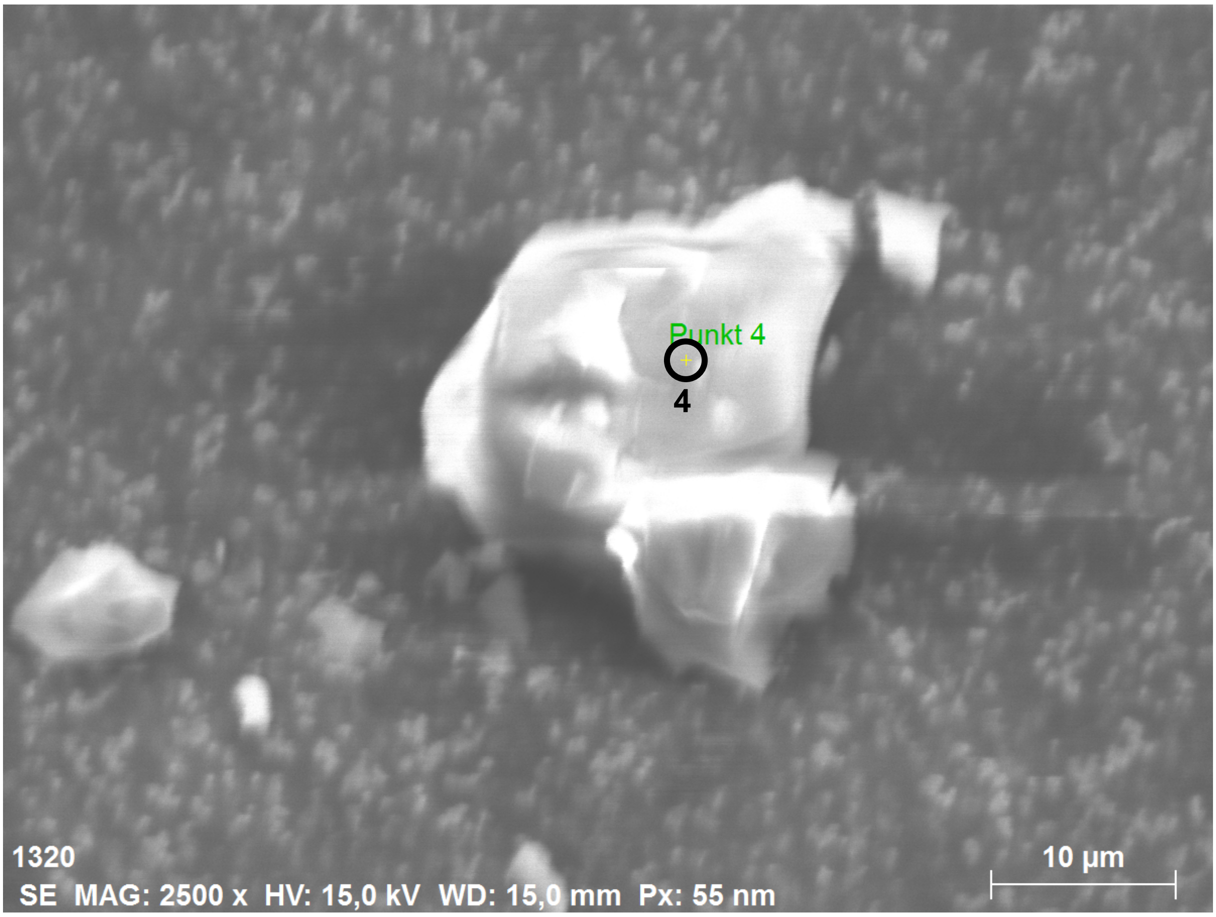


**Figure S60.** SEM image of crystalline $[Cp'La_2Cl_2]_{10}$ (**8**) in position 4, where EDX measurement was performed.

**Table S29.** Results of EDX measurement at position 4 (Fig. S60)

| Element | norm. wt. % | norm. atom % | 3 σ | norm. wt. % calc. | norm. atom % calc. |
|---|---|---|---|---|---|
| Si | 11.54 | 24.53 | 1.01 | 11.81 | 25.00 |
| La | 58.62 | 25.20 | 3.34 | 58.39 | 25.00 |
| Cl | 29.84 | 50.27 | 2.00 | 29.80 | 50.00 |

**Table S30.** Averaged results of all EDX measurements for **4** (3 measurements)

| Element | norm. wt. % | norm. atom % | 3 σ | norm. wt. % calc. | norm. atom % calc. |
|---|---|---|---|---|---|
| Si | 11.49 | 24.32 | 0.94 | 11.81 | 25.00 |
| La | 58.26 | 24.94 | 3.13 | 58.39 | 25.00 |
| Cl | 30.25 | 50.74 | 1.89 | 29.80 | 50.00 |

## References

[1] a) U. Behrens, R. E. Dinnebier, S. Neander, F. Olbrich, *Organometallics* **2008**, *27*, 5398-5400; b) P. Jutzi, W. Leffers, B. Hampel, S. Pohl, W. Saak, *Angew. Chem. Int. Ed.* **1987**, *26*, 583-584; *Angew. Chem.* **1987**, *99*, 563-564.

[2] M. Zimmermann, N. Å. Frøystein, A. Fischbach, P. Sirsch, H. M. Dietrich, K. W. Törnroos, E. Herdtweck, R. Anwander, *Chem. Eur. J.* **2007**, *13*, 8784-8800.

[3] H. M. Dietrich, C. Zapilko, K. W. Törnroos, R. Anwander, *Organometallics* **2005**, *24*, 5767-5771.

[4] COSMO, v. 1.61, Bruker AXS Inc., Madison, WI, 2012.

[5] APEX 3, v. 2016.5-0; Bruker AXS Inc., Madison, WI, 2012.

[6] SAINT, v. 8.34A; Bruker AXS Inc., Madison, WI, 2010.

[7] L. Krause, R. Herbst-Irmer, G. M. Sheldrick, D. Stalke, *J. Appl. Cryst.* **2015**, *48*, 3-10.

[8] G. Sheldrick, *Acta Crystallogr., Sect. A* **2015**, *71*, 3-8.

[9] C. B. Hübschle, G. M. Sheldrick, B. Dittrich, *J. Appl. Cryst.* **2011**, *44*, 1281-1284.

[10] C. F. Macrae, I. J. Bruno, J. A. Chisholm, P. R. Edgington, P. McCabe, E. Pidcock, L. Rodriguez-Monge, R. Taylor, J. van de Streek, P. A. Wood, *J. Appl. Cryst.* **2008**, *41*, 466-470.

[11] W. H. Zachariasen, *Acta Cryst.* **1948**, *1*, 265-268.
